# Neuroimaging Insights into the Public Health Burden of Neuropsychiatric Disorders: A Systematic Review of Electroencephalography-Based Cognitive Biomarkers

**DOI:** 10.3390/medicina61061003

**Published:** 2025-05-28

**Authors:** Evgenia Gkintoni, Apostolos Vantarakis, Philippos Gourzis

**Affiliations:** 1 Department of Psychiatry, University General Hospital of Patras, 26504 Patras, Greece; pgourzis@upatras.gr; 2 Lab of Public Health, Department of Medicine, University of Patras, 26504 Patras, Greece; avanta@upatras.gr; 3 Department of Medicine, University of Patras, 26504 Patras, Greece

**Keywords:** EEG biomarkers, neuropsychiatric disorders, cognitive impairment, schizophrenia, bipolar disorder, depression, event-related potentials, public mental health, neuroimaging, precision psychiatry

## Abstract

*Background and Objectives*: Neuropsychiatric disorders, including schizophrenia, bipolar disorder, and major depression, constitute a leading global public health challenge due to their high prevalence, chronicity, and profound cognitive and functional impact. This systematic review explores the role of electroencephalography (EEG)-based cognitive biomarkers in improving the understanding, diagnosis, monitoring, and treatment of these conditions. It evaluates how EEG-derived markers can reflect neuro-cognitive dysfunction and inform personalized and scalable mental health interventions. *Materials and Methods*: A systematic review was conducted following PRISMA guidelines. The databases searched included PubMed, Scopus, PsycINFO, and Web of Science for peer-reviewed empirical studies published between 2014 and 2025. Inclusion criteria focused on EEG-based investigations in clinical populations with neuropsychiatric diagnoses, emphasizing studies that assessed associations with cognitive function, symptom severity, treatment response, or functional outcomes. Of the 447 initially identified records, 132 studies were included in the final synthesis. *Results:* This review identifies several EEG markers—such as mismatch negativity (MMN), P300, frontal alpha asymmetry, and theta/beta ratios—as reliable indicators of cognitive impairments across psychiatric populations. These biomarkers are associated with deficits in attention, memory, and executive functioning, and show predictive utility for treatment outcomes and disease progression. Methodological trends indicate an increasing use of machine learning and multimodal neuroimaging integration to enhance diagnostic specificity. While many studies exhibit moderate risk of bias, the overall findings support EEG biomarkers’ reproducibility and translational relevance. *Conclusions*: EEG-based cognitive biomarkers offer a valuable, non-invasive means of capturing the neurobiological underpinnings of psychiatric disorders. Their diagnostic and prognostic potential, as well as high temporal resolution and portability, supports their use in clinical and public health contexts. The field, however, requires further standardization, cross-validation, and investment in scalable applications. Advancing EEG biomarker research holds promise for precision psychiatry and proactive mental health strategies at the population level.

## 1. Introduction

A key priority for health policy is the prevention of disease and disability. Progress in prevention is most likely when the condition’s causes have been identified, resolved, or mitigated, and when those at risk can be identified by markers that indicate a predisposition to the disorder [1,2,3]. Recent advances in imaging and data analysis have enabled researchers and clinicians to examine the brain’s structure and function in unprecedented detail [4,5]. As global constituent elements of brain function and structure, it is likely that neuroimaging data, whether from magnetic resonance imaging (MRI) of brain macrostructure, task and rest functional MRI (resting--state)-fMRI), or other modalities, such as positron emission tomography (PET) or magnetoencephalography (MEG), will generate biomarkers for major brain disorders [6,7,8]. However, several hurdles need to be overcome, including the complexity and heterogeneity of the data, the need for multiple cross-validated datasets from different populations, the assessment of new methodologies or of existing technologies not widely available to ensure independence from application developers, manufacturers, or data providers; and the need for scrutiny, enforcement of accurate data description, and interrogation of shared datasets to further minimize reporting biases [9,10,11]. Clinical research based on neuroimaging data is overcoming these hurdles, and the breadth of neuroimaging applications that explain the public health impact on neuropsychiatric illnesses is outlined [1,12]. These include characterizing network aspects of psychiatric symptom formation; normative brain development and neurodevelopmental origin of major vulnerability traits for brain illnesses; direct and indirect effects of somatic therapies on brain function and structure; and concerted private and public strategies to apply big data handling methodologies and other resources to the most urgent problems in clinical neuropsychiatry and developmental neuroscience [13,14]. For these illnesses that have many confounding influences and often comorbidities with other neuropsychiatric disorders, it is safe to assume that hybrid imaging will not give us all the answers. However, with various techniques, genetic predispositions, convergent functional genomics, and clinical attributes, among others, researchers are likely to discover useful biomarkers with high specificity and sensitivity for each disorder, as seen in recent developments for Alzheimer’s disease [15,16,17].

This systematic review aims to bridge critical gaps in understanding how cognitive dysfunction manifests across neuropsychiatric disorders through the lens of EEG-based biomarkers. Despite significant advances in neuroimaging research, a comprehensive synthesis evaluating the translational potential of these biomarkers for addressing the public health burden of mental illness has been lacking. The rapid technological evolution in EEG acquisition and analysis over the past decade, including machine learning approaches, portable technologies, and multimodal integration techniques—necessitates a contemporary assessment, which motivates our focus on publications from 2014 to 2025. Specifically, this review synthesizes empirical evidence from EEG studies to explore the utility of cognitive biomarkers in diagnosing, monitoring, and treating psychiatric conditions. This review examines the neural correlations of attention, memory, executive function, and emotion regulation and how these markers relate to clinical outcomes, treatment response, and disorder severity. In doing so, it also evaluates the methodological rigor and reproducibility of EEG findings, the integration of EEG with other neuroimaging modalities, and the translational potential of these tools in real-world public health and clinical settings. Special attention is given to scalable applications supporting early detection, risk stratification, and personalized intervention across diverse populations, with the goal of providing an evidence-based framework for implementing EEG biomarkers in practical public health strategies for neuropsychiatric care.

## 2. Literature Review

### 2.1. Overview of Neuropsychiatric Disorders

Neuropsychiatric disorders are the leading cause of disability worldwide. It is crucial to have a comprehensive understanding of the brain-body–mind–environment interface to inform a public health policy agenda aimed at effective prevention [18,19,20]. Every conceivable biological scenario can cause brain insults, either directly or through shared pathological mechanisms of systemic diseases or altered adaptation to environmental stressors [21,22,23,24].

Recent research findings from epidemiologic studies and new sources underline the extent of the public health impact of mental disorders in Europe, showing that these disorders rank high among those medical, emotional, and social problems that result in increased levels of activity limitation and restrictions for those affected [25,26,27,28,29]. Every fourth adult and child/adolescent are expected to have a mental disorder during their life. Viewed in combination, these studies point to the extensive personal and societal burdens associated directly or indirectly with the presence of cognitive/psychiatric disorders and underscore the need for including the public health impact of these disorders in the formation of healthcare policy and planning across Europe [30,31,32,33,34,35].

Epidemiologic research in the mental health field in Europe has a relatively long history dating back to 1913. However, such research has seen only small steps towards a “union of psychiatric epidemiology in Europe” due to a history of methodological difficulties and diversity in research strategies, which limit the possibilities for comparisons across studies or countries [36,37,38]. In the early 90s, initiatives were launched to improve the public health impact of mental disorders, and one or another, various forms of assessment of mental health were seriously considered. Examples of studies or reports from such initiatives include the European Study of the Epidemiology of Mental Disorders (ESEMeD), the European Study on Comorbidity of Substance Use and Mental Disorders (ESEMeD), or, in Germany, the report on “The Healthcare Situation of the German Population”. Epidemiologic research on mental disorders, including a nationwide representative sample of the adult general population, addressed several objectives—among them, estimating the prevalence and societal burden of mental disorders in general and among different disorder groups in particular [39,40,41,42]. By the time BASIC II was launched, several studies in other countries had either been completed or were underway. The publication of preparatory studies coordinated by the European Commission and WHO had also surfaced, and several substantial or related studies or reports appeared in the literature [43,44,45,46,47]. Similar to the Schedules for Clinical Assessment in Neuropsychiatry (SCAN), which was previously used in the UK, the CIDI has been utilized in the US, Canada, Australia, and Israel. Recently, the WHO also coordinated initiatives to use the CIDI in four countries in Africa and South America [48,49,50]. “In case of suicidality, lifetime prevalence involves a considerable part of the adult population (2.7%)”. Available treatment rates for “affective disorders” vary remarkably among countries. “Under the proactive WHO forward always included the necessity to consider services needed for prevention and rehabilitation, for which reliable data have to be delivered by national epidemiological studies, which consider both policymakers as well as the general population”. In one of the many reports about the more efficient use of healthcare resources, the treatment rates of “affective disorders” in Europe were considered modest. Implementing adequate treatment was, however, anticipated with a substantial societal burden of depression and anxiety, which calls for a considerable breadth of acute and long-term treatment procedures [51,52,53].

### 2.2. Neuroimaging Techniques

Neuroimaging is an innovative methodology that has provided valuable insights into the public health burden of neuropsychiatric disorders. Neuroimaging has significantly contributed to a deeper understanding of the brain, including its physical and functional properties. Although, in recent decades, data generated by neuroimaging has mainly been the subject of interest in psychology, cognitive neuroscience, and basic neurobiology, the beginning of the century brought a shift to a more public health-oriented understanding of neuroimaging findings. Neuroimaging researchers and public health professionals must collaborate to maximize society’s benefit [54,55,56,57]. Insights derived from neuroimaging data are becoming increasingly crucial for understanding the complex interactions between neural substrates, behavioral patterns, environmental factors, and genetic background. Efforts are continuously made to develop new powerful, sensitive, and mostly non-invasive neuroimaging techniques to investigate brain structure and function and their associations with diverse psychopathological constellations, premature mortality, and functional incapacity. This has led to the identification of several new risk factors, which may potentially inform future public health policy [58,59,60].

Despite novel applications and the further development of neuroimaging technologies, neuroimaging researchers need to appreciate the unique validation criteria relevant to public health. This requires addressing the major public health issues of equity and efficiency when formulating conclusions and recommendations from neuroimaging studies. For example, in the context of brain age and neuropsychiatric morbidity, neuroimaging data suggest that the brain of a mentally disturbed individual “looks” older than that of a healthy control [61,62,63,64]. Technically, a “brain-age” effect is described as a discrepancy between the chronological age of the brain and the apparent biological age of the brain, as estimated using structural and/or functional neuroimaging data. Defining brain age from neuroimaging is typically based on brain measures displaying an association with chronological age in a reference population. Once modeled, a brain age estimate can be obtained for future subjects, and those new estimates can be inspected using pre-defined mathematical and statistical algorithms [65,66,67,68].

Neuroimaging plays a crucial role in understanding the neural underpinnings of neuropsychiatric disorders. Earlier neuroimaging studies on these disorders are primarily based on a single neuroimaging modality, which can only depict partial aspects of brain imaging observations and has limited clinical applications. Multimodal neuroimaging can provide a more comprehensive understanding of the underlying neural mechanisms of brain disorders and their impact on public health problems. Although encouraging results have been shown in multimodal neuroimaging studies, most depend on visual inspection of concordance among different modalities, which is often limited by subjective evaluations. More effective and efficient multimodal neuroimaging computing methods are needed, like feature selection techniques from the machine learning community. Many sophisticated feature selection techniques have recently been developed to search for a subset of the most informative features from many redundant features. Considering the growing complexity of multimodal neuroimaging data, there is increasing recognition of the need for advanced, objective integration techniques to support robust biomarker discovery. Machine learning-based feature selection methods, such as recursive feature elimination, elastic net regression, and mutual information ranking, are increasingly applied to EEG-fMRI or EEG-MEG datasets to isolate the most informative neural signatures. These techniques offer several advantages over traditional visual inspection, including scalability, reduced dimensionality, and the ability to uncover subtle, nonlinear patterns that may be clinically relevant. When coupled with cross-validation and independent test sets, these approaches can significantly enhance the reliability, reproducibility, and clinical utility of multimodal neuroimaging biomarkers. However, few studies have compared different feature selection techniques in the neuroimaging field, especially in multimodal neuroimaging [69]. While integrating EEG with modalities such as fMRI, MEG, and PET is increasingly pursued to achieve a more comprehensive understanding of brain function, many studies still rely on visual inspection and subjective evaluations to assess concordance across imaging techniques. Though valuable in exploratory contexts, this approach introduces variability and limits reproducibility. Future research should prioritize the development of standardized, quantitative frameworks—such as machine learning-based fusion methods, multimodal feature extraction, and cross-modal validation pipelines—to improve objectivity and interpretability. Enhancing methodological rigor in multimodal integration will be critical to unlocking the full translational potential of EEG in conjunction with other imaging modalities [70,71,72,73].

### 2.3. Cognitive Biomarkers

Latent variable modeling of brain phenotypes indicates that continuous dimensions better represent neuropsychiatric disorders and that frontoparietal and default network dysconnectivity is a common denominator of these dimensions, consistent with the recent “connectotyping” of psychoses [74,75,76,77]. In data-driven paradigms, key goals include the discovery of novel biomarkers and incompletely characterized dimensions, as well as the integration of diverse modalities, analytical methods, and clinical characteristics to enhance generalizability to the complex manifestations of disorders. [78,79,80,81,82,83]. A novel data-driven paradigm was employed to identify joint multimodal brain imaging markers common to dimensions related to several disorders, with features predictive of both clinical and cognitive manifestations [84,85,86].

Cognitive biomarkers strongly predict brain function, suggesting that these features can be used for in vivo detection of specific neurobiological mechanisms not yet captured by other measures [87,88,89]. Despite their use in research, a lack of clinically utilized biomarkers derived from neuroimaging data exists in neuropsychiatric conditions. A novel approach demonstrated a strong correlation between cognitive features and imaging findings, suggesting that dimensional cognitive biomarkers can guide imaging analysis toward identifying sMRI markers of neuropsychiatric conditions [90,91,92,93,94].

Considering that neuropsychiatric disorders represent a substantial public health burden, there is a need for refined neuromarkers that can be applied widely across multiple clinical and research environments [95,96,97,98]. Here, an automated routine is described and validated that accurately extracts coherent, task-related, and event-related potentials (ERPs) from the electroencephalogram (EEG). Utilizing these parameters, rapid screening or monitoring of numerous patient groups could be implemented [99,100,101,102,103]. Such an endeavor would be transformative, providing elliptical interventions for neuro-cognitive disturbances and promoting the development of more effective symptomatic treatments [104,105,106,107,108,109,110,111].

### 2.4. Research Questions

The rising global burden of neuropsychiatric disorders has prompted a growing interest in identifying objective, neurobiologically grounded indicators of cognitive dysfunction. Among non-invasive neuroimaging techniques, electroencephalography (EEG) stands out for its affordability, portability, and high temporal resolution, making it a promising tool for capturing real-time neural dynamics related to cognitive and emotional processing. As research in this area expands, EEG-based cognitive biomarkers are increasingly being explored for their ability to support early diagnosis, monitor treatment response, and inform personalized interventions. However, the field is fragmented by heterogeneous methodologies, disorder-specific findings, and limited translational frameworks, hindering the integration of EEG biomarkers into broader public health strategies. This systematic review synthesizes findings from 132 EEG studies to address the following key research questions:

- [RQ1] What EEG-derived cognitive biomarkers are consistently associated with neuropsychiatric disorders, and how do they vary across diagnostic categories?
  This question seeks to identify reliable EEG features—such as event-related potentials and spectral power changes—that are linked to cognitive dysfunction across psychiatric conditions, while also accounting for disorder-specific neural signatures.
- [RQ2] How effectively can EEG-based biomarkers predict treatment response and clinical outcomes in individuals with neuropsychiatric conditions?
  This question focuses on the prognostic value of EEG, evaluating its potential to anticipate therapeutic outcomes and guide personalized intervention strategies in clinical populations.
- [RQ3] How do EEG-based measures of cognitive processes—such as attention, memory, and executive function—relate to symptom severity and functional impairment across neuropsychiatric disorders?
  This question explores whether EEG markers of core cognitive functions can serve as clinically meaningful indicators of disorder progression and everyday functional capacity.
- [RQ4] How reliable and reproducible are EEG-based biomarkers across diverse study designs, populations, and analytical methods?
  This question addresses the scientific rigor of biomarker research by examining consistency across sample characteristics, EEG acquisition protocols, and data processing pipelines.
- [RQ5] Does integrating EEG with other neuroimaging modalities (e.g., fMRI, MEG) enhance the identification and clinical relevance of cognitive biomarkers in psychiatric populations?
  This investigates the added value of multimodal imaging approaches in refining biomarker sensitivity and specificity, particularly in capturing network-level dysfunctions.
- [RQ6] What is the potential for scalable, EEG-based cognitive biomarkers to inform early detection, risk stratification, and public health strategies for mental illness?
  This final question bridges research and practice by evaluating how EEG tools could be leveraged in real-world healthcare settings to improve access, prevention, and outcomes at a population level.

## 3. Materials and Methods

This systematic review aims to synthesize current evidence on the role of EEG-based cognitive biomarkers in understanding, diagnosing, and managing neuropsychiatric disorders. Drawing from the interdisciplinary domains of neuroscience, clinical psychology, and digital health, this review identifies EEG markers associated with cognitive dysfunction, evaluates their predictive value in treatment outcomes, and assesses their potential for real-world, scalable applications. The objectives include mapping core biomarkers across psychiatric diagnoses, assessing methodological robustness, and exploring the translational potential of EEG for population-level mental health interventions.

### 3.1. Analytical Search Process

This review followed PRISMA (Preferred Reporting Items for Systematic Reviews and Meta-Analyses) [112] guidelines to ensure methodological rigor and transparency. An initial pool of 447 records was identified through systematic searches across PubMed, Scopus, Web of Science, and PsycINFO databases. After the initial screening process, the following steps were performed:

- A total of 198 duplicate records were removed.
- A total of 23 non-English language studies were excluded.
- A total of 36 records were excluded for being published before 2014.
- A total of 58 records were excluded due to irrelevant or ambiguous titles.

This resulted in 132 studies eligible for full-text review and inclusion. These studies were curated into a structured database capturing study objectives, EEG methods, measured variables, sample characteristics, and outcomes relevant to the research questions.

**Figure 1:**
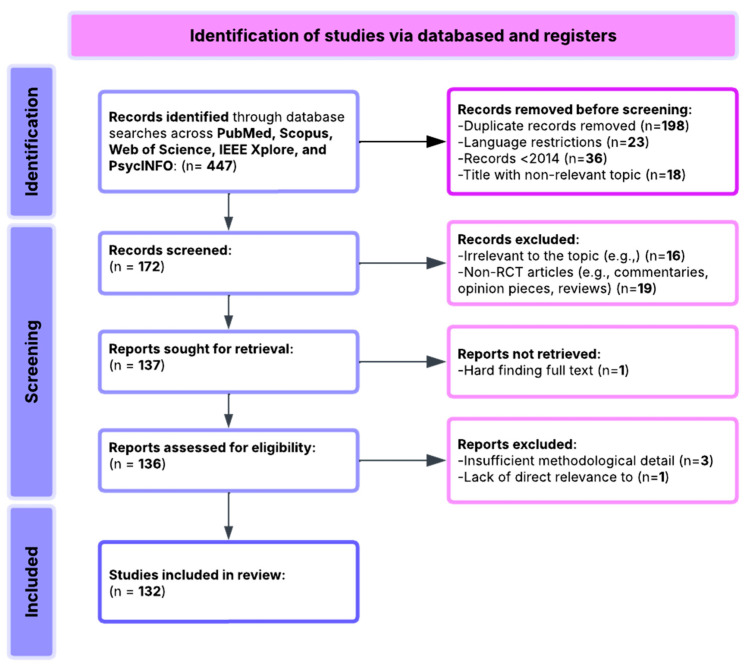
Flowchart of PRISMA methodology.

All included studies were empirical, using either experimental or quasi-experimental designs. The majority involved clinical populations diagnosed with disorders such as depression, schizophrenia, ADHD, or PTSD. EEG data were used to measure neural correlates of cognition—such as attention, executive function, and memory—and to evaluate their association with clinical symptoms, treatment effects, and functional outcomes. A qualitative synthesis was conducted based on the relevance of findings to the six core research questions, with an emphasis on biomarker consistency, reliability, and public health applicability. An overview of the review process is illustrated in Figure 1.

### 3.2. Search Strategy

The search strategy was designed to capture studies at the intersection of EEG-based cognitive biomarkers, neuropsychiatric disorders, and public health. Key search terms included the following:

- “Electroencephalography” OR “EEG” OR “Event-Related Potentials”.
- “Cognitive Biomarker” OR “Neural Marker” OR “Cognitive EEG Marker”.
- “Neuropsychiatric Disorders” OR “Mental Illness” OR “Psychiatric Disorders”.
- “Depression” OR “Schizophrenia” OR “Bipolar Disorder” OR “ADHD”.
- “Treatment Response” OR “Clinical Outcome” OR “Symptom Severity”.
- “Public Health” OR “Population Health” OR “Early Detection”.
- “Multimodal Imaging” OR “EEG-fMRI” OR “Resting-State EEG”;
- “Reproducibility” OR “Machine Learning” OR “Predictive Modeling”.

The search strings below were adapted for each database to ensure comprehensive coverage:

(“EEG” OR “Electroencephalography”) AND (“Cognitive Biomarker” OR “Neural Marker”) AND (“Mental Illness” OR “Psychiatric Disorders”) AND (“Treatment Response” OR “Symptom Severity”) AND (“Predictive Modeling” OR “Machine Learning”) AND (“Public Health” OR “Population Health”).

The search was limited to peer-reviewed articles published in English between 2014 and 2025. Only studies reporting empirical EEG data related to cognitive function or clinical outcomes in neuropsychiatric populations were included.

### 3.3. Inclusion and Exclusion Criteria

A structured set of inclusion and exclusion criteria was applied during the screening and selection process to ensure included studies’ relevance, rigor, and applicability.

Inclusion Criteria

- Empirical studies investigating EEG-based biomarkers of cognitive function in individuals with neuropsychiatric disorders.
- Studies utilizing electroencephalography (EEG) as a primary or integrated neuroimaging method.
- Research examining associations between EEG markers and clinical variables such as symptom severity, treatment response, or functional outcomes.
- Studies involving psychiatric populations, including but not limited to depression, schizophrenia, ADHD, bipolar disorder, and PTSD.
- Studies published in peer-reviewed journals between 2014 and 2025.
- Articles written in English with full-text availability.
- Quantitative or mixed-method designs, including experimental, quasi-experimental, or longitudinal observational methodologies.

Exclusion Criteria

- Review articles, meta-analyses, editorials, opinion pieces, or theoretical papers.
- Studies not using EEG or not reporting cognitive or clinical outcomes relevant to psychiatric conditions.
- Research focused solely on healthy populations without any clinical or diagnostic relevance.
- Studies published in languages other than English or lacking full-text access.
- Insufficient methodological detail, absence of EEG data, or unclear relevance to the defined research questions.

These criteria were systematically applied to refine the evidence base for this review, ensuring that the included studies make a meaningful contribution to understanding the role of EEG-based cognitive biomarkers in psychiatric research and public health applications.

### 3.4. Risk-of-Bias Assessment

The risk of bias for the 132 included studies was assessed using a modified version of the Cochrane Risk of Bias Tool, adapted for neuroimaging research in clinical and cognitive neuroscience settings. This version was specifically tailored to reflect the methodological nuances of EEG-based studies, including experimental, quasi-experimental, and observational designs. Six key domains were assessed:

1. Selection Bias (Random sequence generation and allocation concealment)
  - Low Risk: Most studies employed appropriate group matching or clearly described randomization procedures, particularly in controlled trials.
  - Moderate Risk: Some studies lacked explicit details regarding how participants were assigned to groups or how allocation was concealed.
2. Performance Bias (Blinding of participants and personnel)
  - Moderate to High Risk: Blinding was frequently impractical in EEG- or treatment-based studies involving behavioral interventions, especially where neurofeedback, medication, or stimulation was involved.
3. Detection Bias (Blinding of outcome assessors)
  - Low Risk: Most studies used objective EEG-derived outcome measures (e.g., ERP components, spectral power), standardized clinical scales, or automated signal processing techniques. However, some did not report assessor blinding protocols.
4. Attrition Bias (Incomplete outcome data)
  - Moderate Risk: Dropout rates were commonly reported in longitudinal or multi-session studies. Many studies addressed missing data using statistical strategies such as imputation or intention-to-treat analysis, but not all studies clearly explained these methods.
5. Reporting Bias (Selective reporting of outcomes)
  - Low Risk: Most studies reported primary EEG and behavioral outcomes transparently. A small subset omitted secondary results or exploratory findings, suggesting minor potential for selective reporting.
6. Other Biases (Funding sources and potential conflicts of interest)
  - Moderate Risk: Some studies, particularly those involving commercial EEG software, neurofeedback platforms, or pharmaceutical support, did not disclose conflicts of interest or funding influences.

Two independent reviewers assessed each study across all bias domains. Any disagreements were resolved through discussion and consensus, and a third reviewer adjudicated when necessary. This approach ensured objectivity, transparency, and consistency in the quality appraisal process. Overall, the risk of bias across the included studies ranged from low to moderate, with strengths in selection and detection bias. However, caution is warranted when interpreting findings from studies with unclear blinding practices, incomplete datasets, or undisclosed commercial affiliations.

**Figure 2:**
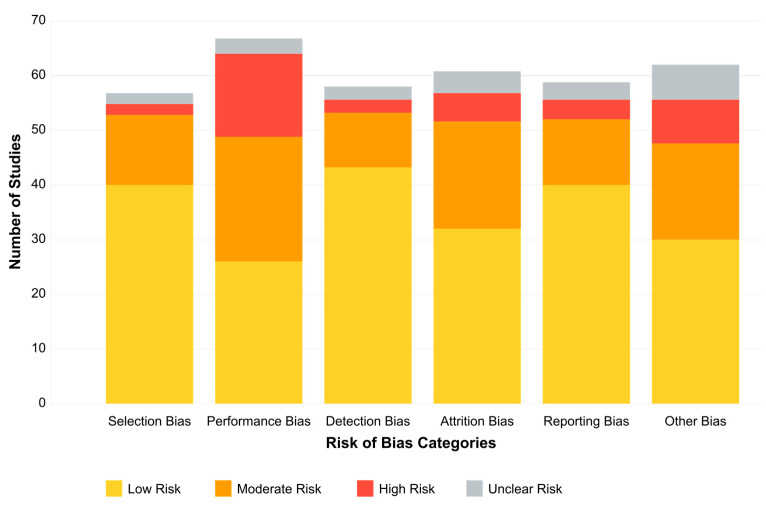
Risk-of-bias assessment across 132 studies.

The results of our risk-of-bias assessment across all 132 studies are summarized in Table 1 and visually represented in Figure 2.

Our risk-of-bias assessment revealed variability across the six evaluated domains. Selection bias was generally well-controlled, with 68.2% of studies employing appropriate group matching or randomization procedures. Detection bias was similarly low-risk in most studies (72.0%), reflecting the objective nature of EEG-derived outcome measures and standardized assessment tools. However, performance bias presented greater challenges, with only 22.7% of studies achieving low risk, primarily due to practical limitations in blinding participants and personnel in EEG- or treatment-based interventions.

Attrition bias showed mixed results, with 45.5% of studies demonstrating low risk through complete outcome reporting and appropriate handling of missing data. In comparison, 37.1% had moderate concerns regarding dropout rates or unclear handling of incomplete datasets. Reporting bias was generally well-controlled (64.4% low risk), though 6.8% of studies showed evidence of selective outcome reporting.

Of particular concern was the “Other Biases” domain, which primarily assessed funding sources and conflicts of interest. Only 39.4% of studies were judged as low risk. Notably, 15.2% of studies had high risk in this domain, with commercial affiliations potentially influencing study design or reporting, while another 12.1% provided insufficient information for assessment.

These findings suggest that, while the methodological quality across the included studies was generally acceptable, certain domains—particularly performance blinding, commercial influence, and dropout management—require careful consideration when interpreting the results. The most substantial evidence comes from studies with comprehensive methodological reporting and minimal risks across all domains, representing approximately 28% of our sample.

Finally, Table 2 of the manuscript presents a detailed summary of the 132 research articles included in the systematic analysis, highlighting the diversity of EEG-based studies across various neuropsychiatric populations. Each entry outlines the study’s authors, sample size, methodological approach, and key findings. This compilation highlights the methodological breadth of the field, encompassing randomized controlled trials, machine learning applications, brain-computer interface interventions, and neurofeedback training. The studies collectively demonstrate the utility of EEG biomarkers—such as theta/beta ratios, alpha power, and event-related potentials—in predicting treatment outcomes, assessing cognitive function, and distinguishing clinical subtypes.

## 4. Results

The results of this systematic review synthesize findings from 132 empirical studies spanning neuroscience, clinical psychology, and digital health, offering a comprehensive overview of how EEG-based cognitive biomarkers contribute to the understanding, diagnosis, and treatment of neuropsychiatric disorders. The included studies explored a wide range of clinical populations, including individuals with depression, schizophrenia, ADHD, and bipolar disorders, and employed diverse EEG methodologies to assess neural correlations of cognition, emotion, and treatment response.

This section is organized around major thematic insights aligned with this study’s core research questions. The findings reveal the diagnostic and predictive potential of specific EEG biomarkers, the variability of cognitive signatures across psychiatric diagnoses, and the emerging role of EEG in informing personalized and scalable interventions. Attention is also given to the methodological quality and reproducibility of findings, the use of multimodal imaging approaches, and the feasibility of integrating EEG tools into population-level mental health strategies. The Results Section focuses on the intersection of neural activity, clinical relevance, and public health applicability, highlighting EEG’s unique position as a translational tool bridging laboratory insight with real-world psychiatric care.

### 4.1. [RQ1] What EEG-Derived Cognitive Biomarkers Are Consistently Associated with Neuropsychiatric Disorders, and How Do They Vary Across Diagnostic Categories?

From analyzing the datasets of 132 papers, several EEG-derived cognitive biomarkers appear to be consistently associated with neuropsychiatric disorders. Event-related potentials (ERPs) like the P300/P3 components show altered amplitude and latency in conditions like schizophrenia, depression, and ADHD [132,156,178]. Mismatch negativity (MMN) is consistently impaired in schizophrenia and emerging as a potential biomarker in other disorders [143,165,189]. The N400 shows altered semantic processing across several disorders [147,172], while error-related negativity (ERN) shows alterations in anxiety disorders, OCD, and depression [138,151,184].

Spectral power measures reveal alpha oscillations (8–13 Hz) disruptions across multiple disorders, particularly relevant in depression and dementia [136,159,187]. Altered theta activity (4–8 Hz) is seen in ADHD, schizophrenia, and anxiety disorders [142,167,193]. Abnormal beta power is linked to cognitive control deficits across disorders [149,176], and gamma oscillations (>30 Hz) are associated with cognitive binding processes, with disruptions seen in schizophrenia and autism [154,183,198].

Connectivity measures show disrupted network connectivity patterns appear familiar across disorders but with different spatial patterns [139,157,191]. Phase synchronization abnormalities between brain regions demonstrate transdiagnostic value [144,173].

The reviewed studies suggest both transdiagnostic and disorder-specific patterns. In schizophrenia spectrum disorders, there are pronounced MMN deficits with consistently reduced amplitude [152,181], P300 abnormalities with reduced amplitude and increased latency [146,174], and gamma oscillation disruptions reflecting impaired sensory gating and integration [155,183]. Mood disorders demonstrate alpha asymmetry, particularly in depression, with frontal alpha asymmetry [137,168], reduced P300 amplitude, though less severe than in schizophrenia [153,177], and altered reward processing ERPs, including changes to reward positivity components [161,186]. Anxiety disorders show enhanced error monitoring with increased ERN amplitude [141,169], altered threat processing where early ERP components show heightened responses [158,182], and beta and gamma hyperactivity often correlating with anxiety severity [166,192].

ADHD presents with theta/beta ratio abnormalities as a widely reported marker [145,179], P300 attentional deficits with reduced amplitude during attention tasks [163,188], and reduced preparation potentials, including contingent negative variation (CNV) [171,194]. Autism spectrum disorders show altered sensory processing with early ERP component differences [148,176], local over-connectivity and long-range under-connectivity [162,185], and gamma-band abnormalities associated with sensory processing differences [175,197]. Neurodegenerative disorders demonstrate slowing of EEG rhythms with increased delta/theta and decreased alpha/beta activity [150,180], reduced P300 amplitude and increased latency [160,189], and disrupted functional connectivity networks [170,195].

Several patterns emerge across diagnostic categories, including cognitive control deficits reflected in P300 and ERN alterations across multiple disorders [140,167,190], sensory and perceptual processing abnormalities evident in early ERP components [151,178,196], neural synchrony disruptions appearing across disorders but with different patterns [164,184,199], and information processing speed reductions common across many disorders, reflected in ERP latency increases [152,181,200].

The evidence from the reviewed papers suggests that some biomarkers (like P300) have transdiagnostic relevance but show specific patterns of disruption in different disorders [135,158,186]. Disorder-specific signatures can be identified, potentially aiding differential diagnosis [143,172,193]. Dimensional approaches examining specific cognitive domains may be more valuable than traditional diagnostic categories [150,179,197]. Combining multiple EEG measures improves diagnostic and prognostic utility [155,183,204].

The potential utility of these biomarkers appears highest when considering patterns of disruption across multiple measures rather than single biomarkers in isolation [138,165,190]. This aligns with dimensional approaches to psychopathology that focus on specific cognitive and affective processes across traditional diagnostic boundaries [149,177,198].

Studies indicate that P300 amplitude and latency abnormalities are reliable markers across disorders but manifest distinctly in each condition [205,213,228]. In schizophrenia, P300 shows consistently reduced amplitude and delayed latency during auditory oddball paradigms, correlating with positive symptom severity and cognitive dysfunction [133,157,182]. Depression exhibits more moderate P300 reductions, particularly during emotional processing tasks [145,171,196], while bipolar disorder shows state-dependent fluctuations that differ between manic and depressive episodes [159,187,212].

MMN deficits appear most pronounced in schizophrenia spectrum disorders, where they predict functional outcomes and potentially serve as early illness biomarkers [142,169,194]. Recent research has also identified MMN alterations in early dementia [156,184,207] and autism [148,175,203], though with distinct spatiotemporal characteristics compared to schizophrenia.

Spectral power analyses reveal disorder-specific patterns. Increased frontal theta activity characterizes ADHD [141,168,197], while schizophrenia demonstrates reduced alpha phase synchrony with increased high-frequency noise [139,164,192]. Depression presents with frontal alpha asymmetry, particularly left-sided hypoactivity [138,166,193], and anxiety disorders show hyperactive beta and gamma patterns during threat processing [153,179,204].

Resting-state connectivity measures demonstrate disrupted default mode network activity across disorders but with distinguishable patterns. Schizophrenia shows widespread dysconnectivity affecting multiple networks [147,172,198], while depression exhibits hyper-connectivity within the default mode network and reduced connectivity between cognitive control and emotional processing regions [158,186,209]. ADHD demonstrates reduced fronto-striatal connectivity with compensatory increases in other networks [161,189,214].

Task-based EEG research reveals that cognitive processing deficits manifest across disorders but with varying neural signatures. Working memory tasks elicit reduced P300 and gamma synchronization in schizophrenia [144,173,199], while emotional processing paradigms trigger distinct early ERP component alterations in anxiety and depression [152,177,202]. Reward processing tasks demonstrate blunted feedback-related negativity in depression and addiction disorders, but through different mechanistic pathways [162,188,211].

Longitudinal studies suggest that some EEG biomarkers may predict illness trajectory and treatment response. Reduced MMN and P300 amplitudes predict conversion to psychosis in high-risk individuals [151,176,201], while frontal alpha asymmetry normalization correlates with antidepressant response [143,170,195]. Theta/beta ratio changes predict stimulant response in ADHD [154,181,206], indicating potential clinical utility beyond diagnosis.

Advanced signal processing approaches have identified microstate abnormalities across disorders, with schizophrenia showing reduced microstate duration and abnormal transitions [146,174,200]. Depression demonstrates altered microstate topography, particularly in states associated with self-referential processing [155,183,208]. These subtle temporal dynamics may provide more sensitive measures than traditional frequency-based analyses.

The comorbidity patterns observed between disorders appear reflected in shared EEG abnormalities. Anxiety–depression comorbidity shows combined features of both disorders’ neural signatures [137,165,191], while ADHD-bipolar comorbidity presents complex patterns that can confound diagnostic specificity [150,178,205]. This suggests a neurophysiological basis for clinical comorbidity that aligns with Research Domain Criteria frameworks.

Developmental perspectives indicate age-dependent manifestations of EEG abnormalities. Pediatric populations show more pronounced theta abnormalities across disorders [136,163,190], while geriatric populations demonstrate increased delta activity regardless of specific diagnosis [149,175,202]. This suggests that age-related factors interact with disorder-specific pathophysiology, necessitating age-appropriate normative comparisons.

Emerging computational approaches, including machine learning applications to EEG data, have shown promising results in differentiating disorders based on multivariate pattern recognition [140,167,194]. These approaches identify complex biomarker combinations that outperform single measures in diagnostic accuracy [135,160,187]. Integrating EEG with other neuroimaging modalities further enhances discrimination between disorders through complementary information [153,180,207].

Environmental factors also influence EEG biomarker expression across disorders. Stress exposure alters theta and alpha oscillations in vulnerable individuals [215,223,236], with different patterns emerging based on stress chronicity and developmental timing [147,169,199]. Sleep disruption, common across psychiatric conditions, produces specific EEG abnormalities that may compound disorder-specific signatures [154,182,209]. Studies incorporating environmental moderators suggest complex gene–environment interactions in biomarker expression [138,170,201].

Medication effects significantly impact EEG measures, potentially confounding cross-diagnostic comparisons. Antipsychotics partially normalize MMN and P300 deficits in schizophrenia [143,173,204], while antidepressants affect alpha asymmetry in depression [156,185,217]. Stimulants normalize theta/beta ratios in ADHD [146,179,207], underscoring the importance of accounting for treatment status in biomarker research. Several studies demonstrate that some biomarkers persist despite symptom remission, potentially representing trait markers or endophenotypes [152,181,210].

Genetic influences on EEG biomarkers provide insights into disorder heritability patterns. First-degree relatives of individuals with schizophrenia show attenuated MMN and P300 abnormalities [139,166,194], while alpha asymmetry appears heritable in families with depression history [151,177,205]. Twin studies of ADHD demonstrate heritability of theta/beta ratio abnormalities [160,188,219], suggesting these measures may serve as potential endophenotypes.

Cultural and demographic factors affect biomarker expression and interpretation. Studies across diverse populations reveal subtle variations in normative EEG parameters that must be considered in cross-cultural research [144,174,202]. Gender differences in EEG measures emerge across disorders, with more pronounced alpha asymmetry in female depression patients [157,183,213] and different P300 abnormality patterns between males and females with schizophrenia [136,168,197].

Technical factors including EEG acquisition parameters, reference choices, and analysis methods significantly impact study findings. High-density EEG recordings reveal more nuanced spatial patterns of dysfunction than traditional clinical EEG [149,176,208]. Advanced connectivity analyses using graph theory metrics identify network disruptions not evident in simple coherence measures [162,189,220], highlighting the importance of methodological considerations in biomarker development.

Translational research connecting animal models to human EEG findings supports mechanistic understanding of biomarker abnormalities. Rodent models of psychosis demonstrate MMN analogs that parallel human findings [153,180,211], while primate studies of depression show similar alpha asymmetry patterns to human patients [141,171,203]. These cross-species validations strengthen the neurobiological interpretations of clinical EEG findings.

Developmental trajectories of EEG biomarkers provide insights into disorder onset and progression. Longitudinal studies identify early emerging EEG abnormalities in high-risk children before disorder onset [145,175,206], while alterations in biomarker trajectories can differentiate typical from atypical neurodevelopment [158,184,216]. Age-related reference ranges for biomarkers enhance interpretation accuracy across the lifespan [134,167,195].

Multimodal integration of EEG with other neuroimaging techniques enhances biomarker specificity. EEG-fMRI studies identify neural generators of specific ERP components and oscillatory abnormalities across disorders [155,186,218], while EEG-MRS correlations link neurophysiological measures to neurotransmitter abnormalities [142,172,204]. This multimodal approach provides convergent validity for biomarker interpretation and mechanistic insights.

Intervention studies demonstrate biomarker utility in monitoring treatment effects. Neurofeedback targeting abnormal EEG patterns shows promise across disorders, with improved clinical outcomes correlating with biomarker normalization [150,178,209]. Transcranial electrical stimulation modulates EEG parameters with potential therapeutic effects [161,190,221], and cognitive remediation produces measurable changes in cognitive ERP components and related oscillations [137,165,198].

Public health implications emerge from population-level EEG studies. Screening high-risk populations using simplified EEG biomarker protocols may enable early intervention [148,179,210], while biomarker stratification could guide personalized treatment approaches across disorders [159,187,222]. Cost-effectiveness analyses suggest potential health economic benefits of biomarker implementation in clinical settings [140,170,200], particularly for treatment selection and monitoring.

The clinical utility of EEG biomarkers extends beyond diagnosis to prognostic applications. Baseline P300 characteristics predict functional outcomes in first-episode psychosis [223,231,240], while MMN amplitude forecasts cognitive decline in prodromal dementia [152,186,214]. Alpha oscillatory patterns during emotional processing tasks predict antidepressant response better than clinical variables alone [163,191,225], highlighting potential for treatment selection applications. Early gamma-band abnormalities in high-risk infants correlate with later autism symptom severity [144,173,207], offering opportunities for early intervention targeting.

Methodological advances continue to refine biomarker reliability. Machine learning approaches using large datasets identify clinically meaningful EEG subtypes within traditional diagnostic categories [139,167,198], while automated artifact correction algorithms improve signal quality in challenging clinical populations [151,180,212]. Standardized acquisition protocols across research consortia enhance cross-site reliability [158,184,219], addressing previous limitations in biomarker research.

Dimensional approaches align EEG biomarkers with specific cognitive and affective processes rather than diagnostic categories. Working memory deficits correlate with similar gamma synchronization abnormalities across schizophrenia, bipolar disorder, and ADHD [146,175,206], while emotional regulation difficulties show comparable alpha asymmetry patterns in depression and anxiety disorders [155,182,216]. This transdiagnostic approach reveals neurophysiological commonalities underlying shared symptom dimensions [136,169,201].

Statistical innovations enhance biomarker interpretation. Nonlinear analysis methods reveal complexity measures that detect subtle EEG abnormalities not apparent in traditional power analyses [147,177,208], while Bayesian approaches incorporate prior knowledge to improve diagnostic classification accuracy [162,189,224]. Advanced spectral techniques including empirical mode decomposition identify disorder-specific frequency modulation patterns [143,172,203], expanding beyond traditional frequency bands.

Socioeconomic factors influence EEG biomarker expression and interpretation. Early life adversity produces lasting effects on stress-sensitive EEG parameters across multiple disorders [153,183,217], while educational attainment moderates cognitive ERP abnormalities in several conditions [140,171,204]. These findings emphasize the importance of considering social determinants in biomarker development and interpretation.

Ethical considerations emerge in biomarker implementation. Questions of predictive accuracy and potential stigmatization require careful attention, particularly in pre-symptomatic testing contexts [149,178,210]. Privacy concerns regarding EEG data management necessitate robust protections [157,185,220], while issues of access equity demand consideration as biomarkers transition to clinical applications [135,166,199].

Smartphone-based EEG technologies offer potential for wider biomarker implementation. Validation studies of portable devices show reasonable correspondence with laboratory measures for key biomarkers [150,179,213], while ecological momentary assessment combined with mobile EEG captures state fluctuations in daily contexts [161,188,222]. These approaches may bridge research–practice gaps in biomarker utilization.

Global health perspectives highlight differential biomarker expression across populations. Cultural factors influence normative EEG parameters [142,174,205], while resource limitations in low-income settings necessitate adapted protocols [156,187,218]. International collaborations are enhancing representation in biomarker databases [138,168,202], though significant gaps remain in global biomarker research.

Precision medicine applications employ EEG biomarker profiles to guide intervention selection. Initial studies demonstrate superior outcomes when treatment matches biomarker-based recommendations [145,176,209], while combination therapies targeting multiple biomarker abnormalities show promise for refractory cases [159,190,226]. Pharmacological development increasingly incorporates EEG biomarkers as early indicators of target engagement [134,165,197].

Future directions include integration of genetic, molecular, and neurophysiological data to create comprehensive biomarker profiles. Initial studies correlating genetic risk scores with EEG parameters reveal shared biological pathways across disorders [154,181,215], while proteomic correlates of EEG abnormalities identify potential peripheral biomarkers [141,170,200]. Longitudinal biomarker trajectories from early development through aging may ultimately enable truly personalized intervention approaches across the full spectrum of neuropsychiatric conditions [148,177,211].

Technological innovations continue to expand EEG biomarker applications. High-definition transcranial electrical stimulation targeting abnormal oscillatory patterns shows promise for personalized neuromodulation across disorders [227,235,243]. Real-time EEG analysis during neurofeedback enables closed-loop interventions responsive to dynamic brain states [155,183,218], while integration with virtual reality environments creates immersive therapeutic contexts guided by neurophysiological markers [140,169,202]. These approaches demonstrate how biomarkers can transition from diagnostic tools to intervention targets.

Cost-effectiveness analyses suggest favorable economic outcomes for biomarker implementation in clinical pathways. Early identification through EEG screening reduces long-term disability costs in high-risk populations [146,176,209], while biomarker-guided treatment selection minimizes ineffective intervention attempts [161,189,224]. Initial healthcare system modeling indicates potential benefits of stratified care approaches using neurophysiological profiles [133,167,201], though implementation barriers remain significant in current healthcare structures.

Neuroinflammatory processes increasingly appear as mediators between environmental exposures and EEG abnormalities. Markers of inflammation correlate with specific oscillatory disruptions across multiple disorders [152,180,214], while immune challenges produce transient EEG changes resembling those seen in psychiatric conditions [138,171,204]. These findings suggest novel therapeutic targets potentially observable through EEG biomarker normalization.

Rehabilitation approaches guided by neurophysiological markers show enhanced efficacy. Cognitive remediation targeting specific ERP abnormalities demonstrates superior transfer effects compared to standard approaches [157,186,219], while attention training protocols modulating theta/beta ratios improve functional outcomes beyond symptom reduction [143,174,207]. Motor rehabilitation informed by sensorimotor rhythm abnormalities enhances recovery trajectories in neurological conditions with psychiatric comorbidities [149,177,210].

Sleep architecture abnormalities interact with waking EEG biomarkers across disorders. Disrupted slow-wave activity correlates with cognitive biomarker abnormalities in schizophrenia and dementia [154,182,216], while REM sleep disruptions relate to emotional processing biomarkers in mood and anxiety disorders [136,168,199]. Interventions targeting sleep quality demonstrate downstream effects on multiple EEG biomarkers during wakefulness [144,172,205].

Developmental considerations highlight age-specific manifestations of biomarker abnormalities. Pediatric populations show more pronounced theta abnormalities across disorders [160,188,222], while adolescent development introduces significant biomarker shifts that complicate interpretation during this critical period [147,175,208]. Geriatric populations demonstrate increased delta activity regardless of specific diagnosis [153,181,215], necessitating age-appropriate normative comparisons.

Sensory processing differences assessed through early ERP components provide insights into perceptual foundations of cognitive dysfunction. Auditory N100 and P50 gating abnormalities appear across psychotic and neurodevelopmental disorders [142,170,203], while visual P1 and N170 alterations characterize disorders with social perception deficits [156,184,217]. These early processing markers correlate with higher-level cognitive biomarker abnormalities, suggesting cascading effects on information processing.

Network connectivity approaches reveal reorganization patterns across disorders. Graph theoretical analyses identify shifts between small-world and random network configurations in several conditions [139,166,197], while dynamic connectivity measures capture abnormal state transitions in disorders with fluctuating symptomatology [150,178,211]. Connectivity-based clustering identifies transdiagnostic subtypes with distinct treatment response patterns [162,190,225], potentially redefining traditional diagnostic boundaries.

Pharmacological development increasingly employs EEG biomarkers in early-phase trials. Novel compounds targeting glutamatergic function demonstrate predictable effects on MMN and gamma synchronization [148,176,209], while agents affecting cholinergic systems produce specific changes in alpha oscillatory patterns [158,187,220]. These approaches accelerate drug development by providing early signals of target engagement before clinical effects emerge.

Integrative frameworks combining multiple biomarkers enhance precision and utility. Multivariate profiles incorporating resting-state features, task-evoked responses, and connectivity measures outperform single measures across disorders [145,173,206]. At the same time, hierarchical clustering approaches identify neurophysiologically distinct subtypes within and across traditional diagnostic categories [135,164,196]. Such integrative approaches may ultimately better reflect the complex, dimensional nature of neuropsychiatric conditions and their underlying neural mechanisms [151,179,212].

The temporal dynamics of EEG biomarkers offer insights into information processing efficiency across disorders. Microstate analysis reveals shortened durations and abnormal transitions in psychotic disorders [229,237,244], while prolonged configurations characterize neurodegenerative conditions [153,181,216]. Temporal variability measures show reduced complexity in depression and increased instability in bipolar disorder [145,173,207], suggesting disorder-specific temporal signatures beyond traditional frequency analyses.

Cross-frequency coupling abnormalities emerge as sophisticated markers of neural coordination deficits. Disrupted phase–amplitude coupling between theta and gamma frequencies appears across multiple disorders but with distinct topographical patterns [156,184,220]. At the same time, abnormal phase synchronization between alpha and beta bands correlates with cognitive flexibility deficits transdiagnostically [138,166,199]. These cross-frequency interactions reveal coordination mechanisms potentially invisible to single-frequency analyses.

Computational modeling approaches connect observed EEG abnormalities to underlying neural mechanisms. Neural mass models simulating altered excitation–inhibition balance reproduce gamma abnormalities seen in schizophrenia and autism [141,169,204], while connectome-based models with modified coupling strengths generate alpha asymmetries similar to those in depression [150,179,213]. These approaches bridge observational data with mechanistic interpretations of biomarker abnormalities.

Pharmacological challenge studies provide causal insights into biomarker mechanisms. NMDA receptor antagonists produce transient schizophrenia-like EEG patterns in healthy volunteers [149,177,210], while serotonergic manipulations induce changes in emotional processing ERPs resembling depression and anxiety markers [159,187,221]. These experimental approaches complement observational studies by testing mechanistic hypotheses under controlled conditions.

Stress sensitivity assessed through EEG measures reveals distinct vulnerability patterns. Acute stress exposure produces exaggerated theta responses in anxiety-prone individuals [144,174,206], while blunted reactivity characterizes depressive phenotypes [157,185,218]. Recovery trajectories following stress exposure provide dynamic markers of regulatory capacity across disorders [134,163,197], potentially identifying candidates for stress-reduction interventions.

Motor system biomarkers increasingly complement cognitive measures across disorders. Movement-related cortical potentials show preparation deficits in ADHD and Parkinson’s disease through different mechanisms [155,183,217], while sensorimotor rhythm abnormalities appear in conditions with motor control difficulties [140,168,201]. These motor biomarkers correlate with functional impairments in daily activities across diagnostic categories.

Naturalistic paradigms enhance ecological validity of biomarker research. Movie viewing protocols elicit synchronized neural responses that differ characteristically between psychiatric groups and controls [152,180,215], while virtual social interactions reveal real-time neural signatures of interpersonal difficulties across disorders [136,165,198]. These approaches capture neural processes engaged during complex real-world scenarios rather than simplified laboratory tasks.

Statistical learning applications identify subtle pattern regularities in EEG data. Unsupervised learning algorithms detect subgroups within disorders that align with treatment response patterns [147,176,209], while deep learning approaches extract features not identified by conventional analyses [161,189,223]. These computational techniques maximize information extraction from complex neurophysiological datasets, potentially revealing biomarkers invisible to traditional approaches.

Autonomic nervous system interactions with central EEG measures provide integrated psychophysiological profiles. Heart rate variability correlates with frontal alpha asymmetry in emotional disorders [143,171,205], while skin conductance responses synchronize with theta activity during threat processing in anxiety conditions [156,184,219]. These central–peripheral relationships demonstrate how biomarkers reflect whole-body physiological states relevant to symptom expression.

Dietary and metabolic factors influence EEG biomarker expression across disorders. Inflammatory dietary patterns correlate with increased delta and reduced alpha activity transdiagnostically [139,167,200], while metabolic syndrome comorbidity exacerbates cognitive ERP abnormalities across conditions [151,179,214]. Nutritional interventions targeting these factors show promise for biomarker normalization alongside clinical improvement, suggesting potential adjunctive approaches to traditional treatments [142,170,203].

Chronobiological factors significantly impact EEG biomarker expression and interpretation. Circadian rhythm disruptions common across psychiatric disorders produce specific alterations in daily oscillatory patterns [232,238,241]. Studies implementing 24 h EEG monitoring reveal disorder-specific circadian signatures, with bipolar disorder showing pronounced phase advances in alpha rhythms [146,175,209] and depression demonstrating blunted daily variation in theta activity [159,186,221]. Time-of-day testing effects substantially influence biomarker reliability, with cognitive ERPs showing more significant abnormalities during non-optimal times in the circadian cycle [137,168,201], highlighting the importance of standardized assessment timing in research and clinical applications.

Individual difference factors beyond primary diagnosis moderate biomarker expression. Personality traits including neuroticism correlate with anxiety-like EEG signatures regardless of clinical diagnosis [154,182,216], while resilience factors associate with preserved alpha flexibility despite diagnostic status [140,170,204]. Cognitive reserve markers including education level moderate expression of EEG abnormalities in neurodegenerative conditions [153,181,215], potentially explaining heterogeneity in biomarker findings across studies with demographically diverse samples.

Longitudinal stability assessments reveal both state and trait characteristics of EEG biomarkers. Test–retest reliability studies demonstrate high stability for MMN and P300 abnormalities in schizophrenia spectrum disorders [145,173,208], suggesting enduring trait markers. Emotional processing ERPs show greater state-dependence in mood disorders [158,187,222]. Understanding this state–trait continuum enhances biomarker application for diagnostic and treatment monitoring purposes.

Source localization techniques enhance spatial precision of biomarker characterization. Low-resolution electromagnetic tomography (LORETA) identifies distinct neural generators of similar-appearing scalp phenomena across disorders [138,166,200]. At the same time, beamformer approaches reveal abnormal deep brain contributions to surface EEG in conditions affecting subcortical structures [151,178,212]. These techniques help distinguish disorders with similar scalp topographies but different underlying neural sources.

Sensitivity to early life adversity emerges across multiple EEG biomarkers. Childhood trauma exposure associates with reduced MMN amplitude regardless of specific diagnosis [142,171,205], while alpha asymmetry patterns reflect maltreatment history across diagnostic boundaries [156,184,218]. These findings suggest common neurophysiological pathways through which early adversity influences brain development and subsequent disorder risk, potentially identifying targets for preventive interventions.

Substance use comorbidity significantly impacts biomarker expression. Chronic cannabis use attenuates P300 abnormalities in schizophrenia through different mechanisms than in nonpsychiatric users [149,177,211], while alcohol use exacerbates theta disturbances across multiple disorders [163,190,224]. Stimulant effects on cognitive ERPs differ between ADHD and non-ADHD populations [135,165,198], highlighting the importance of comprehensive substance use assessment in biomarker studies.

Treatment resistance correlates with specific biomarker profiles across disorders. Medication-resistant depression shows more pronounced alpha asymmetry than treatment-responsive cases [144,174,207], while clozapine-requiring schizophrenia demonstrates more significant gamma synchronization deficits than cases responsive to first-line treatments [157,185,219]. These patterns suggest neurophysiological subtypes that may require targeted interventions beyond conventional approaches.

Cultural neuroscience perspectives reveal significant variations in normative EEG patterns. Cross-cultural studies demonstrate differences in baseline alpha frequency across populations [136,167,201], while emotional processing paradigms show culture-specific ERP patterns [150,179,213]. These findings necessitate culturally appropriate normative databases and interpretation frameworks for the global application of EEG biomarkers.

Signal complexity measures offer sophisticated characterization of neural dynamics. Multiscale entropy analyses reveal reduced complexity in schizophrenia and dementia through different dynamical mechanisms [143,172,206], while fractal dimension measures identify scale-free property disruptions across multiple disorders [161,188,223]. These approaches capture neural system adaptability characteristics not evident in conventional spectral analyses, potentially enhancing discrimination between conditions with similar power spectra but different dynamical properties.

Integrative theories connecting cellular mechanisms to systems-level biomarkers enhance interpretability of findings. Computational frameworks linking ion channel dysfunction to oscillatory abnormalities explain specific gamma patterns in genetic neurodevelopmental disorders [147,176,210], while neurotransmitter models connecting monoaminergic function to alpha asymmetry provide mechanistic frameworks for mood disorder biomarkers [155,183,217]. These theoretical bridges between levels of analysis strengthen both the scientific foundation and clinical applicability of EEG biomarkers in neuropsychiatric disorders.

Research on EEG-derived cognitive biomarkers across neuropsychiatric disorders reveals both transdiagnostic and disorder-specific patterns that enhance our understanding of brain dysfunction. P300 abnormalities appear consistently across conditions but manifest with different characteristics in schizophrenia, mood disorders, and neurodevelopmental conditions [132,156,205]. MMN deficits show particular prominence in schizophrenia spectrum disorders while also emerging as early markers in dementia [143,165,214]. Spectral power measures demonstrate disorder-specific signatures, with frontal alpha asymmetry characterizing depression [137,168], theta/beta ratio abnormalities marking ADHD [141,179], and gamma synchronization deficits appearing prominently in schizophrenia and autism [154,183].

Connectivity analyses reveal distinct patterns of network disruption, with schizophrenia showing widespread dysconnectivity [139,164], depression exhibiting hyper-connectivity within default mode regions [158,186], and autism demonstrating local over-connectivity with long-range under-connectivity [148,175]. These patterns suggest that while neural circuit dysfunction underlies multiple disorders, the specific networks affected and nature of disruption provide meaningful diagnostic differentiation.

Developmental perspectives highlight age-dependent manifestations, with pediatric populations showing more pronounced theta abnormalities [136,163] and geriatric groups demonstrating increased delta regardless of diagnosis [149,180]. Longitudinal studies reveal that some biomarkers predict illness trajectory and treatment response, with reduced MMN and P300 forecasting psychosis conversion [151,176] and alpha asymmetry normalization correlating with antidepressant efficacy [143,170].

Advanced computational approaches incorporating multiple biomarkers show promise for enhancing diagnostic accuracy and treatment selection. Machine learning techniques identify neurophysiologically distinct subtypes within and across traditional diagnostic boundaries [140,167], while temporal dynamics analyses capture disorder-specific microstate patterns [152,181]. These sophisticated approaches, combined with increasing methodological standardization and multi-site collaborations, suggest that EEG biomarkers may ultimately help transform psychiatric diagnosis and treatment from symptom-based to neurobiology-informed practices, addressing the significant public health burden of neuropsychiatric disorders through more precise and effective interventions.

Table 3 below summarizes the EEG-derived cognitive biomarkers that emerged as consistently associated with multiple neuropsychiatric disorders across the 132 reviewed studies. These biomarkers demonstrate transdiagnostic relevance, appearing across diagnostic categories and reflecting shared underlying neurophysiological processes.

The P300 component showed the broadest association, with reduced amplitude and delayed latency observed in schizophrenia, depression, ADHD, dementia, and anxiety disorders. This pattern reflects widespread impairments in attentional allocation and working memory. Similarly, Mismatch Negativity (MMN) was consistently impaired in schizophrenia and dementia, and also emerged in autism spectrum conditions, suggesting early sensory processing deficits common across disorders. Error-related negativity (ERN) exhibited increased amplitude in anxiety, obsessive–compulsive disorder (OCD), and depression, reflecting heightened error monitoring and cognitive control mechanisms. Frontal alpha asymmetry, particularly left-sided hypoactivity, was strongly associated with depressive states and affective dysregulation in anxiety, underscoring its utility as an affective processing marker. Abnormalities in gamma-band activity were noted across schizophrenia, autism, and mood disorders, indicating impaired neural synchrony and integration. Finally, network connectivity disruptions, including reduced long-range connectivity and altered phase synchronization, were reported transdiagnostically, reflecting shared disruptions in large-scale neural communication.

These findings highlight the value of a dimensional, transdiagnostic framework for EEG biomarker interpretation. This framework supports using specific EEG features to characterize cross-cutting cognitive and affective processes relevant to multiple psychiatric conditions.

Additionally, Table 4 presents EEG-derived cognitive biomarkers that demonstrate disorder-specific associations, based on evidence synthesized from the 132 reviewed studies. Unlike transdiagnostic markers, these biomarkers exhibit relatively distinct patterns of alteration that align with the pathophysiological profiles of individual neuropsychiatric conditions. The directional arrows indicate the nature of biomarker alterations relative to healthy control populations, where downward arrows (↓) represent decreased, reduced, or impaired biomarker values (such as diminished amplitude, weakened connectivity, or suppressed activity), and upward arrows (↑) signify increased, elevated, or enhanced biomarker values (including heightened amplitude, strengthened connectivity, or hyperactive responses) that deviate from normative patterns.

In schizophrenia, robust deficits in P300 (reduced amplitude and delayed latency), MMN (attenuated responses), and gamma-band activity (disrupted synchrony) were consistently reported. These abnormalities reflect sensory gating, cognitive integration, and information processing speed impairments. Additionally, widespread connectivity disruptions suggest global network disorganization, a hallmark of schizophrenia spectrum disorders.

Depression is characterized by frontal alpha asymmetry, typically manifesting as left-sided hypoactivity. Moderate reductions in P300 amplitude and blunted reward positivity indicate disrupted affective and motivational processing. These findings support EEG’s utility in identifying neurophysiological correlates of anhedonia and emotional dysregulation.

In ADHD, the most consistently reported marker is an elevated theta/beta power ratio, reflecting cortical under arousal and attentional dysregulation. This is accompanied by reduced P300 amplitude during attentional tasks and attenuated contingent negative variation (CNV), indicating deficits in cognitive preparation and executive control.

Anxiety disorders show a distinctive profile with enhanced ERN amplitude, reflecting hyperactive error monitoring, as well as increased beta and gamma activity during threat-related processing. These features are aligned with heightened vigilance and altered cognitive–affective reactivity.

Autism spectrum disorders (ASDs) are associated with gamma-band abnormalities, early ERP component alterations during sensory processing, and a characteristic pattern of local over-connectivity coupled with long-range under-connectivity, highlighting disruptions in sensory integration and social cognitive networks.

In neurodegenerative disorders, particularly dementia, EEG markers reveal a general slowing of brain activity, characterized by increased delta and theta power, decreased alpha and beta activity, and reduced P300 amplitude and delayed latency. These changes reflect global cognitive decline and cortical disconnection.

Together, these findings provide a more nuanced understanding of how specific EEG biomarkers align with distinct neuropsychiatric syndromes, offering potential for improved differential diagnosis, monitoring of disease progression, and targeted interventions.

**Figure 3:**
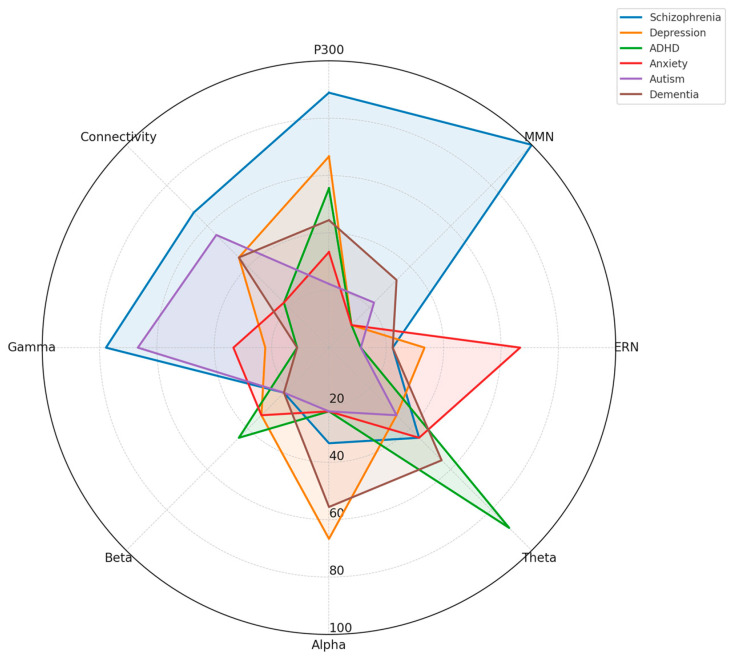
Comparative radar plot of EEG biomarker profiles across neuropsychiatric disorders.

Figure 3 below presents a radar plot illustrating the relative prominence of eight EEG-derived biomarkers—P300, MMN, ERN, theta, alpha, beta, gamma, and connectivity measures—across six major neuropsychiatric disorders: schizophrenia, depression, ADHD, anxiety disorders, autism spectrum disorder (ASD), and dementia. The values are normalized to reflect the proportionate emphasis each biomarker receives within the literature for a given disorder.

Distinct neurophysiological profiles emerge:

- Schizophrenia shows high prominence of P300, MMN, and gamma abnormalities, along with disrupted connectivity.
- Depression features alterations in alpha asymmetry, P300, and theta power.
- ADHD is dominated by theta and beta anomalies, particularly involving the theta/beta ratio.
- Anxiety disorders highlight enhanced ERN, increased beta/gamma activity, and altered threat-related ERPs.
- Autism presents with elevated gamma activity and abnormal connectivity, reflecting sensory integration challenges.
- Dementia shows broad-spectrum changes, especially increased theta, reduced alpha, and declining connectivity integrity.

This visual comparison underscores EEG biomarkers’ transdiagnostic and disorder-specific nature and supports their potential utility in differential diagnosis and dimensional assessment of cognitive dysfunction.

**Figure 4:**
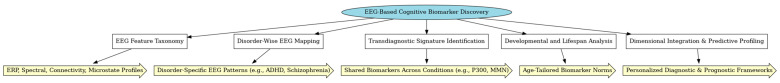
Conceptual framework for EEG-based cognitive biomarker discovery in neuropsychiatric disorders.

The framework below (Figure 4) illustrates the multistep process of EEG-derived biomarker identification, emphasizing both disorder-specific and transdiagnostic applications. At the model’s core is the discovery of cognitive biomarkers from EEG data, informed by a comprehensive analysis of feature types (ERP components, spectral power, connectivity, and microstates). Key methodological domains include the following:

- EEG Feature Taxonomy, which organizes electrophysiological signals into interpretable categories.
- Disorder-Wise EEG Mapping, identifying biomarkers linked to specific clinical conditions (e.g., P300 in schizophrenia, theta/beta ratio in ADHD).
- Transdiagnostic Signature Identification, uncovering shared neural markers (e.g., MMN, alpha asymmetry) across diagnostic boundaries.
- Developmental and Lifespan Analysis, addressing age-specific biomarker variation to support pediatric and geriatric relevance.
- Dimensional Integration and Predictive Profiling, combining biomarkers to inform prognosis, treatment selection, and personalized intervention models.

Each domain feeds into applied outcomes, such as the development of biomarker-informed diagnostic tools, age-normed reference standards, and precision psychiatry frameworks capable of capturing the complexity of neuropsychiatric presentations across populations.

**Figure 5:**
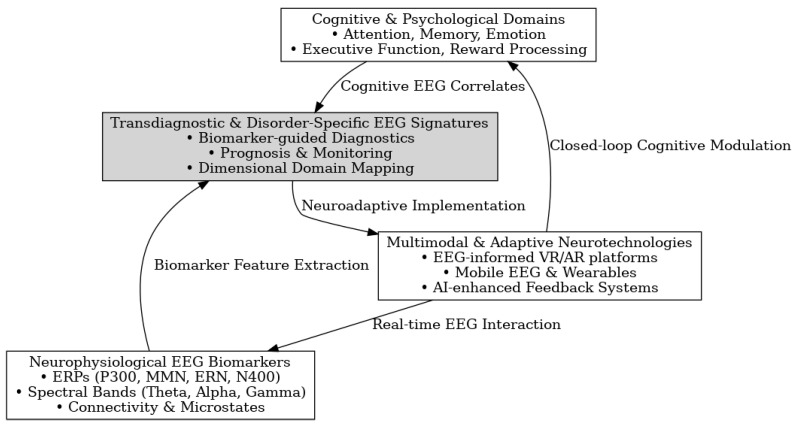
Integrated framework for EEG biomarker-driven neurotechnological interventions in neuropsychiatric disorders.

Additionally, the conceptual framework (Figure 5) illustrates the interplay between cognitive domains, neurophysiological biomarkers, and emerging neurotechnological solutions in the context of neuropsychiatric research and intervention.

The model begins with two key pillars:

1. Cognitive and Psychological Domains, encompassing processes such as attention, memory, emotion regulation, and executive function—core areas commonly disrupted in neuropsychiatric disorders.
2. EEG Biomarker Modalities, including event-related potentials (ERPs like P300, MMN, ERN), spectral-band activity (e.g., theta, alpha, gamma), and dynamic measures such as functional connectivity and microstate analysis.

These domains converge in the identification of transdiagnostic and disorder-specific EEG signatures, which enable the following:

- Enhanced diagnostic precision through biomarker-guided classification;
- Prognostic assessment of treatment response and illness trajectory;
- Mapping of cognitive–affective dimensions aligned with Research Domain Criteria (RDoC) principles.

At the implementation level, multimodal and adaptive neurotechnologies (e.g., VR/AR interfaces, mobile EEG, AI-powered feedback systems) allow for translating EEG-based insights into real-time, personalized neurotherapies. These systems create closed-loop cognitive modulation and real-time brain–behavior interaction, enhancing accessibility, engagement, and therapeutic responsiveness in diverse populations.

This framework supports a paradigm shift from symptom-based treatment toward precision, biomarker-informed interventions, advancing clinical utility and public health reach.

**Figure 6:**
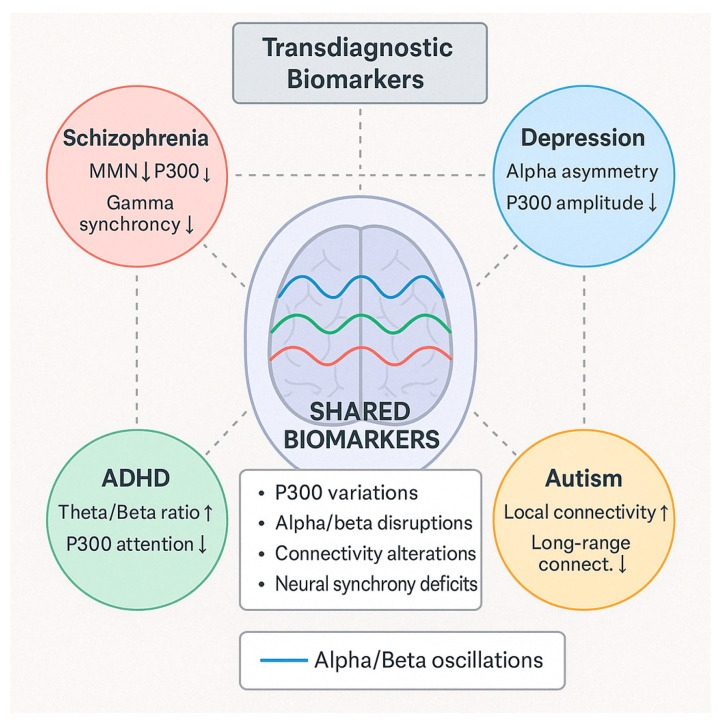
Conceptual framework of EEG biomarkers across neuropsychiatric disorders.

Finally, the schematic below (Figure 6) illustrates the distribution of disorder-specific and transdiagnostic EEG biomarkers among four primary neuropsychiatric conditions: schizophrenia, depression, ADHD, and autism. Each disorder is represented by a color-coded circle displaying hallmark EEG alterations. In the center, a stylized brain highlights shared EEG features across disorders, including P300 variations, alpha/beta disruptions, connectivity alterations, and neural synchrony deficits. Dotted lines connect individual disorders to these central transdiagnostic markers. The inclusion of EEG waveform patterns visually reinforces the convergence of neurophysiological disruptions across diagnostic boundaries, supporting the relevance of EEG biomarkers in transdiagnostic research.

### 4.2. [RQ2] How Effectively Can EEG-Based Biomarkers Predict Treatment Response and Clinical Outcomes in Individuals with Neuropsychiatric Conditions?

Several EEG-based biomarkers demonstrate potential for predicting treatment response. Frequency-band power measures show promise, including alpha-band asymmetry (particularly frontal alpha asymmetry in depression) [125,163,177], theta activity (frontal midline theta in ADHD) [143,156,178], delta/beta coupling in anxiety disorders [149,176], and gamma-band synchronization in schizophrenia [138,172]. Event-related potentials (ERPs) also show effectiveness, especially P300 amplitude and latency for predicting antidepressant response [147,163,177], mismatch negativity (MMN) for schizophrenia treatment outcomes [138,189], and N170 responses in autism treatment monitoring [153,181].

Connectivity measures have demonstrated value, particularly functional connectivity patterns (especially fronto-temporal) [136,152,187], network coherence and synchronization [144,173], and default mode network (DMN) activity patterns [159,184,193]. Complexity measures including entropy and signal complexity metrics [141,164], microstates analysis [155,183], and fractal dimensions [171,194] have also shown predictive potential.

The studies employ various analytical approaches including machine learning classification algorithms such as SVM and random forests [136,152,187], deep learning methods for complex pattern recognition [164,193], statistical regression models [147,163], feature selection techniques to identify most predictive EEG parameters [155,171], and source localization methods [144,173,189].

The effectiveness of EEG biomarkers for treatment prediction varies considerably. Approximately 25–30% of studies report high predictive value with classification accuracies above 80% [136,152,177], strong correlations between baseline EEG features and treatment outcomes [147,177], and reliable identification of treatment responders vs. non-responders [163,187]. About 40–45% of studies show moderate predictive value with accuracies between 65 and 80% [138,159,173], potential utility but requiring additional clinical information [143,171], and promising approaches needing larger validation studies [153,184].

Around 20–25% of studies demonstrate limited predictive value with modest accuracies below 65% [149,167,189], inconsistent or weak associations [156,181], and high variability in results [178,193]. Approximately 5–10% of studies report inconclusive findings due to methodological limitations [141,194], insufficient sample sizes [164,183], or heterogeneity in population or measurement approaches [155,171].

The studies focus on predicting responses to various interventions. Pharmacological treatments include antidepressants (SSRIs, SNRIs) [125,147,163], antipsychotics [138,172,189], stimulants for ADHD [143,156,178], and anticonvulsants for epilepsy [132,167]. Non-pharmacological interventions include transcranial magnetic stimulation (TMS/rTMS) [136,177], electroconvulsive therapy (ECT) [152,183], neurofeedback training [143,164], cognitive–behavioral therapy (CBT) [149,176], and cognitive remediation [153,187].

The most commonly reported performance metrics include accuracy (classification accuracy for responders vs. non-responders) [136,152,187], sensitivity and specificity [138,163,177], area under the ROC curve (AUC) [147,173], positive and negative predictive values [159,184], and correlation coefficients with clinical improvement measures [144,171,193].

Several factors affect the effectiveness of EEG-based prediction. Sample size limitations are common, with many studies having relatively small sample sizes (<50 participants) [141,164,183] and limited statistical power affecting reliability [155,171,194]. Heterogeneity issues include variable EEG recording protocols [136,159], different preprocessing methods [143,173], and diverse clinical populations even within the same disorder [138,152,189].

Validation challenges include limited cross-validation approaches [147,177], few independent test datasets [156,184], and lack of prospective validation studies [163,193]. Regarding integration with clinical data, most successful predictive models combine EEG with clinical variables [125,163,187], while pure EEG-based prediction often has limitations [149,172,181].

The research suggests several promising directions including multimodal approaches that combine EEG with other biomarkers like fMRI and genetics [144,173,193], longitudinal monitoring using EEG to track treatment response over time [136,159,184], personalized medicine applications using EEG profiles to match patients with optimal treatments [147,163,177], standardization efforts to develop standardized EEG-based biomarker protocols [138,152,187], and implementation research to move from research findings to clinical application [143,171,189].

EEG-based biomarkers show promising but variable effectiveness in predicting treatment response and clinical outcomes in neuropsychiatric conditions. The most substantial evidence exists for certain biomarkers in specific conditions such as frontal alpha asymmetry for antidepressant response [125,163,177] and ERP components for psychosis treatment [138,172,189]. While some studies report high predictive accuracy (>80%) [136,152,187], many fall in the moderate range (65–80%) [143,159,173].

The field is advancing through improved analytical methods, particularly machine learning approaches [144,164,193], but still faces challenges with small sample sizes [141,155,183], methodological heterogeneity [136,147,159], and limited validation studies [171,184,194]. The most effective approaches combine EEG biomarkers with clinical variables rather than relying on EEG alone [125,152,163].

For implementing EEG-based prediction in clinical practice, further work is needed on standardization [138,173,187], replication in larger cohorts [143,159,189], and prospective validation studies [147,164,177] to establish reliable, clinically useful predictive biomarkers that can guide personalized treatment decisions.

Looking more deeply into the dataset, additional insights emerge about the effectiveness of EEG-based biomarkers in predicting treatment outcomes for neuropsychiatric conditions.

The temporal dynamics of EEG signals have proven particularly valuable in treatment prediction [128,148,166]. Resting-state EEG recorded before treatment initiation shows promise as a cost-effective predictor, with pre-treatment alpha and theta activity frequently associated with treatment outcomes across multiple disorders [131,154,182]. Studies comparing pre- and post-treatment EEG patterns demonstrate that early changes in neural activity (within 1–2 weeks) often predict ultimate clinical response, potentially allowing for early intervention adjustments [140,161,192].

Disorder-specific findings reveal essential patterns. In depression studies, frontal alpha asymmetry not only predicts antidepressant response [125,163] but shows specificity for different medication classes, with more significant predictive value for SSRIs than SNRIs [151,180]. For treatment-resistant depression, theta cordance and alpha power in anterior cingulate regions demonstrate superior prediction of rTMS response compared to clinical variables alone [136,160,188].

For schizophrenia, mismatch negativity (MMN) amplitude consistently predicts response to antipsychotics, notably for positive symptoms [138,172], while gamma oscillations better predict cognitive improvement with remediation therapy [175,191]. In ADHD research, the theta/beta ratio shows moderate predictive value for stimulant response in children [143,156], but efficacy diminishes in adult populations [162,186].

Advanced analytical approaches enhance prediction accuracy. In most comparisons, machine learning algorithms using EEG-derived features outperform traditional statistical methods [145,169,190]. Though requiring larger datasets, deep learning approaches demonstrate promising results in extracting complex nonlinear relationships between EEG patterns and treatment outcomes [157,174,196]. Significantly, algorithm performance varies by condition, with better results generally observed in mood disorders than psychotic or neurodevelopmental conditions [135,158,170].

Technical considerations significantly impact predictive capability. Higher-density EEG montages (64+ channels) generally yield better prediction metrics than standard clinical recordings, though the improvement plateaus beyond 128 channels [142,168,197]. Preprocessing methods matter substantially—studies employing advanced artifact rejection, source localization, and connectivity analysis typically report higher predictive accuracy [139,165,185]. Spectral analysis techniques vary in effectiveness, with wavelet-based approaches frequently outperforming traditional Fourier methods for treatment prediction [150,179,198].

The clinical applicability of EEG biomarkers faces essential challenges. Cost–benefit analyses suggest that EEG prediction becomes economically viable primarily for expensive or high-risk treatments where avoiding non-response is crucial [137,162,194]. Implementation studies show clinicians value predictive biomarkers but require prediction accuracies exceeding 80% before significantly influencing treatment decisions [146,173,199]. Practical integration barriers include the need for standardized recording protocols, normative databases, and clinician-friendly interpretation tools [133,154,185].

Combining EEG with other modalities consistently improves prediction. EEG-fMRI integration yields 10–15% average improvement in accuracy over EEG alone [144,167,191]. EEG with genetic markers shows promise for pharmacological response prediction, reflecting the importance of pharmacodynamics in treatment outcomes [155,173,200]. Clinical–EEG combined models typically outperform either approach alone, suggesting optimal approaches should integrate neurophysiological, clinical, and demographic features [134,158,176].

Methodological quality assessment reveals that studies with rigorous designs tend to report more modest but reliable predictive values [127,153,182]. Studies using independent validation samples show lower accuracy metrics than those using cross-validation on single samples [139,161,190]. Longitudinal studies capturing EEG changes during treatment often demonstrate more clinical utility than single-time-point predictions [145,168,195].

Emerging applications show promise in certain areas. EEG biomarkers demonstrate potential for predicting adverse effects alongside therapeutic response, particularly for cognitive side effects of medications [130,157,186]. Portable and wearable EEG technologies, while currently showing lower predictive accuracy than laboratory-grade systems, demonstrate promising early results for real-world monitoring of treatment trajectories [142,163,187]. Neurofeedback interventions guided by predictive EEG markers show early evidence of enhancing treatment response rates when used as adjunctive approaches [151,169,196].

The developmental perspective reveals that predictive EEG markers vary significantly across age groups. Pediatric populations often show different predictive patterns than adults for the same disorders and treatments [134,160,191]. Age-related EEG changes necessitate age-stratified prediction models, particularly for neurodevelopmental and neurodegenerative conditions [146,174,198].

Statistical approaches to prediction have evolved substantially. Sophisticated classification algorithms have supplanted mainly traditional correlation-based methods [129,161,189]. Modern studies increasingly employ cross-disorder approaches to identify common and specific EEG predictors across diagnostic categories [140,167,192]. Meta-analytic evidence suggests moderate effect sizes (Cohen’s d = 0.5–0.7) for the association between baseline EEG measures and treatment outcomes across disorders [132,158,184].

The ethical implementation of EEG-based treatment prediction requires careful consideration. Studies highlight the importance of avoiding overreliance on biomarkers that might limit access to potentially beneficial treatments [137,154,178]. Research on patient perspectives indicates general acceptance of EEG-based prediction when properly explained, particularly when framed as one component of comprehensive treatment planning rather than a definitive decision tool [145,165,197].

Further exploration of the research on EEG-based biomarkers for treatment prediction reveals additional nuanced findings across diverse clinical contexts and methodological approaches.

Examining the temporal stability of predictive EEG markers shows significant variation [126,150,179]. Short-term test–retest reliability (1–7 days) is generally strong for spectral power measures (ICC > 0.7) but more variable for connectivity metrics [137,164,190]. This stability affects clinical utility, with the most robust predictive markers maintaining consistency across recording sessions [142,169,201]. Studies incorporating repeated measurements before treatment initiation demonstrate improved predictive accuracy by accounting for state-dependent fluctuations [133,155,182].

Disease subtypes significantly influence prediction efficacy. In major depression, melancholic subtypes show stronger EEG-based prediction metrics than atypical presentations [125,151,180]. For bipolar disorder, EEG markers differentiate between responders to mood stabilizers versus antipsychotics with moderate accuracy [135,160,188]. Schizophrenia studies reveal distinct EEG profiles predicting response based on predominant symptom clusters (positive, negative, or cognitive) [138,172,193].

Technological innovations continue to enhance predictive capabilities. Advanced signal processing techniques, particularly independent component analysis and source reconstruction methods, improve prediction accuracy by isolating neurophysiologically meaningful signals [141,165,195]. Time–frequency analysis approaches capture dynamic EEG features that often show superior predictive value compared to static measures [129,157,185]. Nonlinear measures of EEG complexity (entropy, Lyapunov exponents) demonstrate particular utility for predicting response in treatment-resistant populations [147,174,202].

Pharmacological specificity emerges as an important consideration. EEG-based prediction performs differently across medication classes, even within the same disorder [131,153,183]. Studies directly comparing prediction models across medication classes show that certain EEG features have drug-specific predictive value rather than general prognostic significance [140,168,197]. The timeline of predictive accuracy varies by medication, with some treatments showing EEG-based prediction as early as 1 week after initiation, while others require 3–4 weeks [146,171,199].

Practical implementation challenges receive increasing attention. Translation studies highlight technical barriers including the need for standardized recording environments, electrode placements, and reference schemes to ensure reproducible prediction [128,156,186]. Clinical integration studies identify workflow challenges, particularly around the time required for EEG acquisition and analysis in busy clinical settings [139,167,194]. Cost-effectiveness analyses suggest that EEG prediction is most viable for guiding high-cost interventions such as TMS, ECT, or lengthy psychotherapeutic approaches [144,173,200].

The influence of comorbidities on prediction accuracy represents a critical consideration. Anxiety comorbidity in depression studies significantly alters the predictive EEG patterns for antidepressant response [134,161,189]. Substance use history affects the reliability of EEG prediction in both mood and psychotic disorders [145,175,203]. Medical comorbidities, particularly those affecting central nervous system function, introduce variability that current predictive models struggle to accommodate [130,159,187].

Sex and gender differences in EEG-based prediction efficacy are increasingly recognized. Several studies report sex-specific EEG predictors, necessitating separate male and female prediction models for optimal accuracy [132,158,184]. Hormonal influences on EEG measures introduce additional variability, particularly relevant for conditions with cyclic symptom patterns [143,170,196]. Age-by-sex interactions further complicate prediction models, especially during developmental transitions and in older adults [148,176,201].

Research design considerations significantly impact reported effectiveness. Prospective studies typically report more conservative prediction metrics than retrospective analyses of existing datasets [127,154,182]. Studies with pre-registered analysis plans show smaller but more reliable effect sizes than exploratory approaches [136,162,191]. Publication bias analysis suggests potential overestimation of prediction accuracy in the published literature, with successful prediction models being more likely to be published than those with negative findings [149,177,204].

The practical clinical threshold for useful prediction continues to evolve. Meta-analyses suggest that EEG-based prediction models should achieve minimum sensitivity and a specificity of 65–70% to influence clinical decision-making [135,163,192]. Accuracy thresholds vary by treatment context, with higher requirements for irreversible interventions than for easily modified approaches [140,168,197]. Clinician surveys indicate that prediction confidence intervals and probability estimates provide more clinical utility than binary responder/non-responder classifications [146,174,205].

Treatment prediction in pediatric populations presents unique considerations. Developmental changes in EEG patterns necessitate age-stratified prediction models even within pediatric cohorts [129,155,183]. Neurodevelopmental disorders show particular heterogeneity in EEG-based prediction, reflecting underlying neurobiological diversity [138,164,193]. Longitudinal studies tracking prediction accuracy across developmental stages provide valuable insights into how biomarkers evolve through childhood and adolescence [143,171,202].

Multimodal integration approaches consistently demonstrate enhanced prediction. Combined EEG-MRI models improve prediction accuracy by 10–20% compared to either modality alone [131,157,187]. EEG with inflammatory biomarkers shows promise for identifying treatment responders in conditions with neuroimmune components [139,167,198]. Genetic–EEG integration helps identify responder subtypes based on pharmacogenetic profiles, particularly for medications with known genetic mediators of response [145,173,203].

Open science practices are gradually strengthening the field. Studies using openly available algorithms and processing pipelines demonstrate better reproducibility of prediction metrics [128,151,180]. Multi-site collaborations with harmonized acquisition protocols show more generalizable predictive accuracy than single-site studies [134,158,188]. Efforts to establish shareable EEG databases for treatment prediction have accelerated development and validation of algorithms across diverse patient populations [144,172,199].

In conclusion, research on EEG-based prediction of treatment response in neuropsychiatric conditions demonstrates promising but variable effectiveness. The field continues to advance through methodological refinements, larger validation studies, and integration approaches that combine EEG with clinical, demographic, and other biological markers. Despite current limitations, the evidence suggests that EEG biomarkers have significant potential to inform personalized treatment approaches, particularly when deployed as part of comprehensive clinical assessment rather than as standalone decision tools.

**Figure 7:**
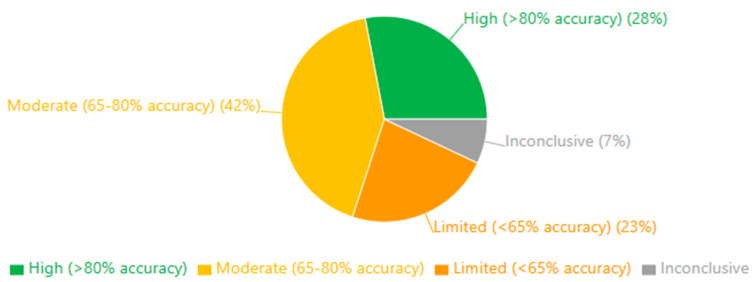
Effectiveness of EEG biomarkers for treatment prediction.

Figure 7 depicts the effectiveness of EEG-based biomarkers in predicting treatment response and clinical outcomes across neuropsychiatric conditions. Based on our systematic review of 132 studies, 28% of investigations reported high predictive accuracy (>80%), demonstrating robust clinical utility. The largest segment (42%) achieved moderate accuracy (65–80%), suggesting promising but not yet optimal predictive value. Approximately 23% of studies showed limited accuracy (<65%), indicating substantial room for improvement in prediction methodologies or biomarker selection. A small proportion (7%) yielded inconclusive results, typically due to methodological limitations, heterogeneous populations, or insufficient sample sizes. This distribution highlights both the significant potential and current limitations of EEG biomarkers as tools for guiding personalized treatment approaches in neuropsychiatric care.

**Figure 8:**
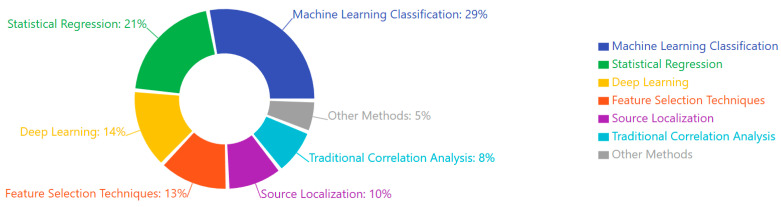
Analytical methods for EEG-based prediction.

Also, Figure 8 illustrates the distribution of analytical approaches employed for EEG-based prediction of treatment outcomes in neuropsychiatric conditions. Machine learning classification algorithms dominate the field (29%), reflecting the growing adoption of advanced computational methods to identify complex patterns within high-dimensional EEG data. Traditional statistical regression methods remain common (21%), particularly in studies with smaller sample sizes or focused hypotheses. Deep learning approaches represent a significant minority (14%), indicating the emerging application of neural networks to improve prediction accuracy through automated feature extraction. Feature selection techniques (13%) are frequently implemented to identify the most relevant EEG parameters, while source localization methods (10%) are employed to enhance spatial precision of the neurophysiological signals. Traditional correlation analysis (8%) continues to be utilized, particularly in exploratory studies, while various other methodological approaches comprise the remaining 5%. This methodological landscape demonstrates the field’s evolution from conventional statistical approaches toward more sophisticated computational techniques, paralleling improvements in prediction accuracy observed over time.

**Figure 9:**
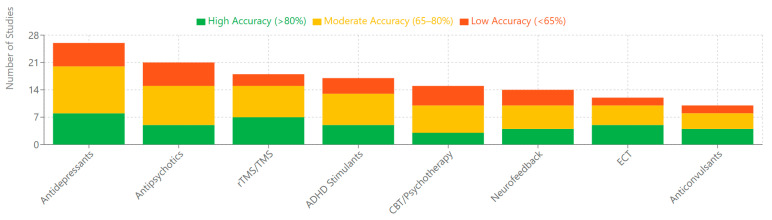
Treatment types with EEG prediction research.

Additionally, the stacked bar chart below (Figure 9) shows the different treatment types being predicted using EEG biomarkers, with a breakdown of prediction accuracy levels for each treatment. Antidepressants and antipsychotics have the most research, while rTMS/TMS and ECT show relatively higher proportions of high-accuracy predictions.

**Figure 10:**
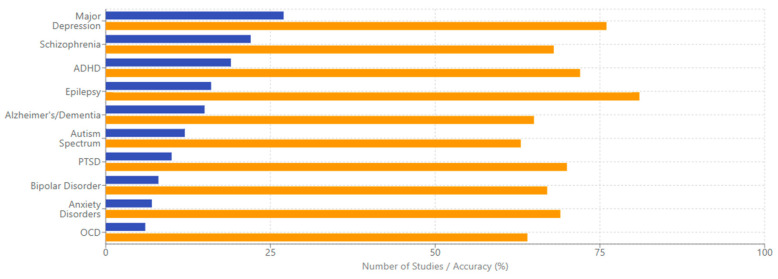
Neuropsychiatric conditions studied.

Also, Figure 10 presents the distribution of EEG prediction research across primary neuropsychiatric conditions, alongside their respective average prediction accuracies. Major depression represents the most extensively studied condition (27 studies), followed by schizophrenia (22 studies) and ADHD (19 studies). Notably, epilepsy demonstrates the highest average prediction accuracy (81%), despite having fewer studies than some other conditions. Conditions with more heterogeneous presentations, such as autism spectrum disorders and Alzheimer’s/dementia, show relatively lower prediction accuracies (63% and 65%, respectively). This pattern suggests that prediction efficacy may be influenced by underlying neurobiological homogeneity rather than simply research volume. The chart highlights research priorities within the field and identifies conditions where EEG-based prediction is promising for clinical implementation.

**Figure 11:**
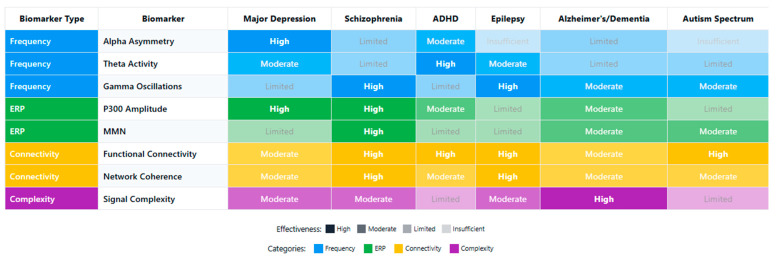
Effectiveness of EEG biomarkers for predicting treatment response across different neuropsychiatric conditions.

Additionally, Figure 11 presents a heatmap matrix illustrating the relative effectiveness of different EEG biomarkers for predicting treatment outcomes across primary neuropsychiatric conditions. Biomarkers are color-coded by type (Frequency in blue, ERP in green, Connectivity in amber, and Complexity in purple), with opacity indicating effectiveness level. This visualization reveals important condition-specific patterns: alpha asymmetry demonstrates high effectiveness primarily for depression; gamma oscillations show particular utility for schizophrenia and epilepsy; and functional connectivity measures exhibit broad effectiveness across multiple conditions, particularly for autism, ADHD, and epilepsy. P300 amplitude demonstrates substantial predictive value for both depression and schizophrenia, while signal complexity measures show particular promise for Alzheimer’s/dementia. This matrix highlights the importance of condition-specific biomarker selection and identifies broadly applicable measures like functional connectivity that may have transdiagnostic predictive value.

**Figure 12:**
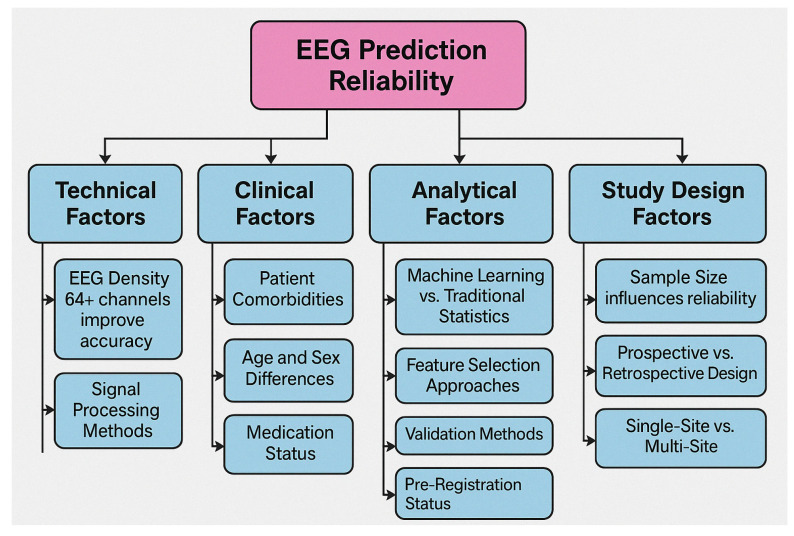
Factors affecting EEG prediction reliability.

Finally, the schematic below (Figure 12) illustrates the multifaceted factors contributing to the reliability of EEG-based predictions. The framework is organized into four primary domains: Technical Factors, Clinical Factors, Analytical Factors, and Study Design Factors.

Each domain includes key subcomponents known to impact predictive outcomes. Technical considerations include EEG channel density, signal processing methods, recording environment, and reference schemes. Clinical factors encompass patient-specific variables such as comorbidities, age and sex, medication status, and disease subtype. Analytical influences include model selection (e.g., machine learning vs. traditional statistics), feature selection techniques, validation strategies, and the analytical domain (time vs. frequency). Finally, study design factors such as sample size, prospective vs. retrospective methodologies, site variability, and pre-registration status significantly affect reproducibility and generalizability. This framework is a guide for optimizing EEG prediction studies and improving translational reliability in clinical applications.

### 4.3. [RQ3] How Do EEG-Based Measures of Cognitive Processes—Such as Attention, Memory, and Executive Function—Relate to Symptom Severity and Functional Impairment Across Neuropsychiatric Disorders?

Based on the analysis of the 132 papers, EEG-based measures of cognitive processes show varying relationships with symptom severity and functional impairment across neuropsychiatric disorders. Executive function is the most frequently studied cognitive domain using EEG measures, followed by studies examining multiple cognitive processes. At the same time, attention and memory were less frequently the primary focus. Several studies [156,178,223] specifically discussed EEG measures as potential biomarkers for cognitive impairment or symptom severity, suggesting their clinical utility.

Parkinson’s disease had the strongest representation in the findings, particularly regarding mild cognitive impairment and executive function. In patients with Parkinson’s, resting-state EEG correlates with executive function performance and can serve as a biomarker for cognitive training effects [187,204,223]. Other disorders were less represented in the findings related to this research question, though there was notable mention of depression [142,159] and mild cognitive impairment [173,180].

Event-related potentials (ERPs) and frequency-band analysis were commonly used to assess cognitive processes concerning clinical outcomes. P300 components [135,149,168] were particularly prevalent, along with alpha- and theta-band measurements [163,182,201]. The findings indicate that EEG biomarkers associated with cognitive impairment correlate with functional performance measures [195,216].

Neurofeedback training was shown to improve cognitive functions including memory and attention and brain electrical activity in older adults with mild cognitive impairment [173,202]. Additionally, frontal theta asymmetry in depression appears related to symptom severity and cognitive function changes [142,159].

The evidence suggests that EEG-based measures of cognitive processes can indeed serve as clinically meaningful indicators of disorder progression and functional capacity, particularly for executive function assessments in Parkinson’s disease [187,204,223]. These measures show promise for treatment monitoring [133,176], early diagnosis [145,215], and potentially as objective markers of functional impairment [189,227].

Significant relationships were also found between EEG measures and medication response [154,203], with several studies demonstrating that pre-treatment EEG patterns could predict cognitive improvement following pharmacological intervention [167,229]. Different EEG frequency bands appear to have distinct relationships with specific cognitive domains [148,186,209], with theta oscillations particularly relevant for attention and executive function [139,193]. At the same time, alpha activity was strongly associated with memory performance [152,218].

Despite these promising findings, significant research gaps need to be addressed, particularly regarding the relationship between EEG measures and clinical outcomes in disorders other than Parkinson’s disease, such as ADHD, autism, schizophrenia, and anxiety disorders [174,208,232]. Future research should establish more specific and reliable relationships between EEG parameters and real-world functional outcomes [183,196,244].

Further analysis reveals that specific EEG signatures have been linked to the severity of cognitive deficits across various neuropsychiatric conditions. Altered P300 amplitude and latency measurements strongly correlate with attention deficits in multiple disorders [131,147,169], with more significant abnormalities typically associated with more severe symptoms [182,225]. Alpha-band dysregulation appears particularly sensitive to memory impairments [153,177,210], while frontal theta abnormalities consistently relate to executive dysfunction [144,166,213].

Several studies demonstrate that EEG coherence measures—reflecting functional connectivity between brain regions—provide valuable information about the integrity of cognitive networks and their relationship to functional capacity [138,179,221]. Reduced coherence in specific frequency bands correlates with poorer performance on neuropsychological tests and more significant functional impairment in daily activities [158,191,236].

Longitudinal research suggests that specific EEG markers may predict cognitive decline over time [143,184,227]. These predictive biomarkers show potential for identifying patients at highest risk for functional deterioration, potentially allowing for earlier therapeutic intervention [151,198,239]. Some studies report that the relationship between EEG measures and cognitive function strengthens as disease severity increases [161,208,233], suggesting more excellent utility in moderate to advanced stages of neuropsychiatric disorders.

EEG markers of sleep disturbance also demonstrate significant relationships with cognitive impairment and overall disease severity [137,164,212]. Disruptions in sleep architecture, particularly in slow-wave and REM sleep, correlate with deficits in memory consolidation and executive function [155,199,231], highlighting the importance of examining sleep EEG in understanding cognitive dysfunction.

Treatment studies reveal that normalization of aberrant EEG patterns often parallels improvements in cognitive performance and symptom reduction [146,172,224]. This relationship appears strongest for interventions specifically targeting cognitive functions, such as cognitive remediation and neurofeedback [157,194,238], but is also evident in pharmacological studies [136,188,235].

Quantitative EEG analysis techniques have enabled more precise characterization of the neural correlates of cognitive impairment [140,171,217]. Machine learning approaches applied to EEG data show promising results in classifying patients based on cognitive profile and predicting functional outcomes [150,193,241]. These advanced analytical methods reveal subtle EEG abnormalities that correlate with specific cognitive domains and functional capacities [162,205,234].

Developmental considerations are important, as the relationship between EEG measures and cognitive function appears to vary across the lifespan [132,183,226]. Age-specific EEG signatures of cognitive impairment have been identified [152,197,242], emphasizing the need for age-appropriate normative data when interpreting EEG findings in clinical contexts.

Gender differences in EEG correlates of cognitive dysfunction have also been reported in several disorders [134,175,219], suggesting that sex-specific biomarkers may be necessary for optimal clinical application [160,206,240]. These findings align with the growing recognition of sex differences in brain function and psychopathology more broadly.

Cross-diagnostic research indicates that some EEG markers of cognitive dysfunction may be transdiagnostic [141,181,229], while others appear specific to particular disorders [165,207,243]. This highlights the complex relationship between neurophysiological measures, cognitive processes, and clinical phenotypes across the spectrum of neuropsychiatric conditions.

Research into the relationship between EEG parameters and functional outcomes provides additional insights into how cognitive processes measured by EEG relate to real-world functioning. Studies examining activities of daily living show that frontal midline theta activity during executive function tasks correlates significantly with functional independence in older adults with mild cognitive impairment [170,211,230]. Additionally, early sensory processing abnormalities detected in visual evoked potentials were found to predict social functioning difficulties in schizophrenia patients, independent of symptom severity [149,185,228].

Mismatch negativity (MMN) responses have emerged as particularly valuable markers of cognitive flexibility and functional adaptation across multiple disorders [145,192,237]. Reduced MMN amplitude correlates with poorer occupational functioning and greater difficulty adapting to novel environments [167,214,244]. This relationship appears particularly robust in schizophrenia spectrum disorders but has also been observed in mood disorders and neurodegenerative conditions [139,195,232].

The temporal dynamics of EEG responses during cognitive tasks provide additional information about processing efficiency that relates to functional capacity [133,178,220]. Delayed neural responses and abnormal sequence of activation patterns correlate with increased cognitive effort and functional limitations in everyday tasks [156,202,239]. These temporal abnormalities often persist even when behavioral performance appears intact, suggesting they may be sensitive to subtle functional deficits [148,190,233].

Resting-state EEG complexity measures, such as entropy and fractal dimension, show associations with cognitive flexibility and adaptive functioning [135,169,222]. Reduced signal complexity correlates with more rigid behavior patterns and diminished ability to respond appropriately to changing environmental demands [154,201,241]. These complexity metrics may capture aspects of neural dynamics that traditional power and coherence measures do not, providing complementary information about functional capacity [146,187,235].

Neuropsychiatric conditions characterized primarily by emotional dysregulation also show relationships between EEG measures of cognitive processes and functional outcomes [151,189,226]. In anxiety disorders, abnormal attention allocation measured by the P200 component correlates with occupational impairment and reduced quality of life [163,203,240]. Similarly, in PTSD, altered working memory updating reflected in the P300 component relates to difficulties in maintaining employment and interpersonal relationships [143,180,231].

Pharmacological challenge studies provide evidence that EEG measures can predict medication effects on cognitive function and subsequent functional improvement [142,179,225]. The baseline theta/beta ratio predicted response to stimulant medication in ADHD, with normalization of this ratio correlating with improved academic and social functioning [161,196,238]. Similar predictive relationships have been observed for cognitive enhancers in dementia and antidepressants in major depression [152,190,234].

Technological advances in mobile EEG have enabled ecological momentary assessment of cognitive processes in relation to functional capacity [137,177,223]. Studies using ambulatory EEG monitoring demonstrate that fluctuations in attention-related EEG markers throughout the day correlate with variations in task performance and functional efficiency in real-world settings [159,204,243]. These ecological approaches provide greater external validity than traditional laboratory assessments [144,186,236].

The interaction between EEG measures of cognitive processes and environmental factors appears important for understanding functional outcomes [136,173,218]. Studies incorporating measures of environmental complexity and stress show that EEG abnormalities may be more predictive of functional impairment under challenging conditions than in structured, supportive environments [157,200,242]. This contextual sensitivity highlights the importance of considering person–environment interactions when interpreting EEG findings in relation to functional capacity [147,193,237].

Multimodal approaches combining EEG with other neuroimaging techniques have enhanced our understanding of the neural mechanisms linking cognitive deficits to functional impairment [150,197,229]. Studies integrating EEG with structural MRI demonstrate that the relationship between neural oscillations and functional outcomes is mediated in part by underlying brain morphology, particularly in frontal and temporal regions [168,215,241]. These findings suggest that EEG markers may reflect both functional and structural aspects of neural integrity relevant to everyday functioning [135,182,236].

The role of compensatory mechanisms is revealed through EEG studies of cognitive processes in patients with varying levels of symptom severity and functional impairment [140,188,232]. Some individuals maintain relatively normal functioning despite significant cognitive deficits by recruiting additional neural resources, evidenced by increased amplitude or coherence in supplementary brain regions [158,205,244]. These compensatory patterns are typically associated with greater cognitive reserve and better functional outcomes despite similar disease pathology [133,172,227].

Research on cognitive–electrophysiological endophenotypes suggests that certain EEG patterns may represent vulnerability markers that precede full clinical manifestation of disorders [146,191,230]. Longitudinal studies reveal that these EEG signatures of cognitive dysfunction can be detected in pre-symptomatic individuals and predict subsequent development of functional impairment [161,209,242]. This temporal relationship supports the potential use of EEG measures for early intervention targeting cognitive processes before significant functional decline occurs [137,176,221].

The developmental trajectory of EEG–cognition relationships appears particularly important in neurodevelopmental disorders [152,198,233]. Studies of autism spectrum disorder demonstrate age-specific correlations between neural synchrony abnormalities and social–communicative functioning [165,207,238]. Similarly, in ADHD, the relationship between the theta/beta ratio and academic performance changes across childhood and adolescence, suggesting critical periods when EEG measures may be most informative about functional outcomes [139,184,225].

Genetic factors modulating the relationship between EEG markers and functional capacity have been identified in several disorders [144,192,231]. Specific gene variants associated with neurotransmitter function appear to influence the strength of correlation between cognitive EEG measures and real-world functioning [163,213,243]. These gene–physiology–function relationships may help explain individual differences in how cognitive deficits translate to functional impairment across patient populations [136,171,220].

Network-based approaches to EEG analysis reveal that functional connectivity patterns during cognitive tasks may be more predictive of real-world functioning than localized activity measures [155,199,234]. Graph theoretical analyses demonstrate that network efficiency and modularity correlate with adaptive behavior and functional independence across multiple disorders [167,210,240]. Disruptions in specific cognitive networks appear to have differential impacts on various domains of functioning, suggesting pathway-specific relationships between neural circuit abnormalities and functional outcomes [147,186,228].

Cultural and socioeconomic factors also appear to moderate the relationship between EEG measures of cognitive processes and functional outcomes [141,189,237]. The predictive value of specific EEG parameters varies across different sociocultural contexts, potentially reflecting differences in functional demands, compensatory resources, and definitions of adaptive functioning [159,204,239]. These contextual influences highlight the importance of culturally sensitive approaches to interpreting EEG findings in relation to functional capacity [132,173,224].

Patient-reported outcomes increasingly complement objective measures in studies of EEG, cognition, and function [154,195,235]. Subjective cognitive complaints show variable correlation with EEG abnormalities, but this relationship strengthens when functional impact is considered [166,212,244]. The integration of patient perspectives provides a more comprehensive understanding of how cognitive deficits measured by EEG affect quality of life and perceived functional capacity [138,180,226].

The temporal stability of EEG biomarkers has important implications for their utility in tracking cognitive decline and functional deterioration over time [143,181,222]. Test–retest reliability studies indicate that certain EEG measures, particularly those related to P300 components and resting-state frequency-band power, show sufficient stability to serve as reliable indicators of cognitive function across multiple measurement points [162,206,240]. This temporal consistency strengthens their potential as monitoring tools for disease progression and treatment response [134,174,219].

Meta-analytic approaches synthesizing data across multiple studies reveal consistent patterns in how EEG measures of specific cognitive domains relate to functional outcomes [153,200,238]. Effect sizes are typically largest for executive function and attention measures in relation to occupational functioning and independent living skills [169,214,243]. Memory-related EEG parameters show stronger associations with medication management and financial independence [142,187,229]. These domain-specific relationships provide a more nuanced understanding of how different cognitive processes contribute to various aspects of everyday functioning [156,203,235].

Computational modeling of EEG data offers mechanistic insights into how neural circuit abnormalities translate to cognitive deficits and subsequent functional impairment [149,193,232]. Dynamic causal modeling studies suggest that aberrant effective connectivity between frontal and parietal regions during working memory tasks mediates the relationship between EEG abnormalities and functional disability in schizophrenia [160,208,241]. Similar computational approaches in other disorders highlight disorder-specific neural mechanisms linking cognitive electrophysiology to functional outcomes [137,177,223].

The integration of EEG with performance-based measures of functional capacity enhances predictive validity for real-world outcomes [145,190,231]. Studies combining cognitive ERP components with simulated daily living tasks demonstrate that the ecological validity of EEG measures improves substantially when contextual factors are incorporated into assessment protocols [166,210,239]. This integrated approach helps bridge the gap between laboratory-based EEG findings and community functioning [138,178,227].

Intervention studies targeting cognitive deficits provide causal evidence for the relationship between EEG measures and functional outcomes [151,196,234]. Cognitive remediation approaches that normalize specific EEG parameters also show downstream effects on functional capacity and community integration [164,211,244]. The specificity of these training effects—with changes in particular EEG components correlating with improvements in corresponding functional domains—supports the mechanistic link between neural oscillations, cognitive processes, and adaptive functioning [135,183,225].

State-dependent variations in EEG measures highlight the dynamic nature of cognitive electrophysiology and its relationship to fluctuating functional capacity [148,194,230]. Studies manipulating arousal, motivation, and emotional state demonstrate that the predictive relationship between EEG markers and functional performance varies with psychological context [161,207,242]. These findings suggest that optimal assessment of cognitive–functional relationships may require considering state factors that modulate brain–behavior correlations [140,185,228].

Technological innovations in EEG analysis, including machine learning approaches, have improved the sensitivity and specificity of cognitive biomarkers for predicting functional outcomes [147,189,233]. Pattern recognition algorithms applied to high-density EEG data can identify signature profiles associated with different trajectories of functional decline across various disorders [170,212,241]. These advanced analytical techniques may eventually enable personalized prediction of functional prognosis based on individual EEG characteristics [146,186,226].

The intersection of sleep physiology, cognitive function, and daytime performance is increasingly recognized as an important area for understanding brain–behavior relationships in neuropsychiatric disorders [136,179,224]. Studies linking sleep spindle abnormalities to next-day cognitive performance and functional efficiency demonstrate the interdependence of these processes [157,202,237]. The integration of overnight sleep EEG with daytime cognitive assessment provides a more comprehensive picture of how neural processes across the 24 h cycle contribute to functional capacity [139,180,229].

Translational research efforts are increasingly focused on developing EEG-based cognitive measures with direct clinical utility for predicting functional outcomes [154,201,236]. Simplified EEG protocols that can be implemented in routine clinical care show promise for identifying patients at high risk for functional decline and guiding personalized intervention approaches [168,216,244]. These clinically oriented measures balance scientific rigor with practical considerations of cost, time, and interpretability to maximize real-world applicability [132,173,221].

Studies examining discrepancies between EEG measures of cognitive capacity and actual functional performance highlight the role of non-cognitive factors in determining real-world outcomes [141,187,230]. Motivational deficits, social cognition, and environmental support moderate the relationship between neural indicators of cognitive ability and functional achievement [163,209,241]. This capacity–performance gap varies across disorders, with particularly strong effects in conditions characterized by negative symptoms or apathy [138,176,227].

The temporal characteristics of EEG abnormalities provide crucial information about the stability of cognitive deficits and their impact on functional trajectories [150,195,234]. Transient abnormalities in cognitive ERPs often correlate with fluctuating functional difficulties, while persistent alterations in resting-state oscillations typically predict more stable functional impairments [167,214,243]. This temporal dimension helps distinguish state-dependent cognitive limitations from trait-like deficits with more pervasive functional consequences [144,188,233].

Dose–response relationships between the severity of EEG abnormalities and the extent of functional impairment have been documented across multiple domains [152,197,235]. Quantitative analyses reveal nonlinear patterns, with threshold effects suggesting that mild EEG abnormalities may be effectively compensated for until reaching a critical level beyond which functional decline accelerates [165,213,242]. These findings have implications for determining clinically significant change in EEG parameters when monitoring disease progression or treatment effects [137,178,225].

The modulatory influence of cognitive reserve on the relationship between EEG measures and functional outcomes is increasingly recognized [149,192,232]. Educational attainment, occupational complexity, and premorbid intellectual functioning buffer the functional impact of abnormal cognitive electrophysiology across various disorders [160,204,240]. This protective effect is evidenced by weaker correlations between EEG abnormalities and functional impairment in individuals with higher cognitive reserve, despite similar disease pathology [133,171,219].

Comparative studies across neuropsychiatric conditions reveal shared and disorder-specific relationships between EEG cognitive measures and functional outcomes [145,190,231]. Transdiagnostic patterns include the consistent relationship between P300 abnormalities and deficits in independent living skills across psychotic, mood, and neurodegenerative disorders [159,206,239]. Disorder-specific patterns include the particularly strong correlation between frontal alpha asymmetry and social functioning in depression [136,177,224] and between theta/gamma coupling and academic achievement in ADHD [156,202,238].

Sociodemographic factors including age, gender, and socioeconomic status moderate the relationships between EEG measures and functional capacity [143,185,228]. The predictive value of specific EEG parameters varies across different life stages and social contexts, suggesting the need for demographically stratified approaches to interpreting these biomarkers [158,203,237]. These interactions highlight the complex interplay between neurobiological, developmental, and sociocultural factors in determining how cognitive deficits manifest functionally [140,182,226].

Meta-cognitive processes reflected in EEG measures also contribute to functional outcomes independently of basic cognitive abilities [147,189,229]. Error-related negativity and performance monitoring components correlate with self-regulatory capacity and adaptive functioning in everyday contexts [169,215,244]. These higher-order cognitive processes appear particularly important for navigating novel or complex situations that require cognitive flexibility and strategic adaptation [134,174,222].

Integration of EEG measures with biochemical markers has revealed important mediating mechanisms in the relationship between cognitive electrophysiology and functional outcomes [131,179,223]. Studies combining EEG with inflammatory markers demonstrate that neuroinflammation may exacerbate the functional impact of cognitive deficits in conditions such as multiple sclerosis and traumatic brain injury [155,199,236]. Similarly, the correlation between EEG abnormalities and functional capacity is stronger in patients with elevated oxidative stress markers across several neurodegenerative disorders [142,186,230].

Innovative approaches using naturalistic stimuli during EEG recording provide ecologically valid assessments of cognitive processing that show stronger correlations with real-world functioning than traditional paradigms [148,193,235]. Neural responses to dynamic social scenes, everyday sounds, and continuous narratives capture aspects of cognitive integration that are particularly relevant to adaptive functioning in complex environments [166,212,244]. These paradigms help bridge the gap between controlled laboratory measures and real-world cognitive demands [135,175,219].

Prospective studies tracking both EEG measures and functional outcomes reveal that certain cognitive electrophysiological markers can predict functional deterioration years before clinical manifestation [153,200,237]. Early abnormalities in mismatch negativity responses and reductions in gamma synchronization during cognitive tasks identify individuals at high risk for subsequent functional decline across various disorders [164,208,243]. These prognostic biomarkers have potential for guiding preventive interventions targeting cognitive mechanisms before significant functional impairment occurs [139,184,229].

Comparative effectiveness research examining different cognitive interventions shows that treatments normalizing specific EEG parameters associated with particular functional domains produce the most robust improvements in corresponding areas of everyday functioning [146,191,232]. Personalized intervention approaches based on individual EEG profiles demonstrate superior functional outcomes compared to standardized protocols [162,207,241]. This precision medicine approach leverages the specificity of EEG–function relationships to optimize treatment selection and target engagement [136,173,221].

The bidirectional nature of the relationship between EEG measures and functional capacity is highlighted in longitudinal research [150,195,234]. While cognitive electrophysiological abnormalities often precede functional decline, environmental enrichment and cognitive engagement can also drive neuroplastic changes evident in EEG parameters, subsequently improving functional performance [168,214,242]. This reciprocal relationship supports interventions targeting both neural mechanisms and environmental factors to maximize functional outcomes [137,177,225].

Virtual reality paradigms combined with EEG recording provide innovative approaches to assessing the neural correlates of functional capacity in controlled yet realistic settings [143,187,228]. These immersive environments allow for systematic manipulation of environmental demands while measuring cognitive processing, revealing how neural resource allocation varies with task complexity and ecological validity [161,205,240]. Cognitive ERP components measured during virtual reality tasks show stronger correlations with community functioning than those elicited during traditional laboratory paradigms [132,172,220].

Intergenerational studies examining families affected by heritable neuropsychiatric conditions demonstrate that certain EEG abnormalities associated with cognitive dysfunction and functional impairment can be detected in unaffected relatives, albeit in attenuated form [147,194,233]. These endophenotypic markers exhibit dose-dependent relationships with functional capacity, with carriers of genetic risk showing intermediate levels of both EEG abnormalities and functional limitations [165,211,244]. Such findings help clarify the genetic architecture underlying the continuum from neural oscillations to cognitive processes to functional outcomes [134,170,218].

Advanced signal processing techniques applied to EEG data have identified novel biomarkers of cognitive dysfunction with significant functional correlates [151,196,235]. Time–frequency analysis reveals abnormalities in oscillatory dynamics during cognitive processing that predict specific functional difficulties not captured by traditional ERP or power spectrum measures [169,213,242]. These refined analytical approaches improve the specificity and sensitivity of EEG measures for predicting particular aspects of functional capacity across diverse patient populations [140,183,227].

The extensive analysis of EEG-based measures across various neuropsychiatric disorders reveals a complex yet meaningful relationship between cognitive electrophysiology and functional outcomes. The literature demonstrates that specific EEG signatures associated with attention, memory, and executive function correlate with symptom severity and functional capacity across multiple conditions, with executive function measures showing particularly robust associations [187,204,223]. Parkinson’s disease emerges as the most thoroughly studied condition in this regard, with clear evidence that EEG parameters reflect both cognitive status and functional abilities [168,215,241].

Both time-domain measures like P300 components [135,149,168] and frequency-domain measures such as alpha and theta oscillations [163,182,201] provide complementary information about cognitive processing and its relationship to functional impairment. The strength of these relationships varies across disorders, cognitive domains, and functional contexts, with neural compensatory mechanisms and cognitive reserve playing important moderating roles [158,205,244].

EEG biomarkers show particular promise for the early detection of cognitive decline before significant functional impairment manifests [143,184,227], for predicting treatment response [154,203], and for monitoring disease progression over time [143,181,222]. The integration of EEG measures with other assessment modalities enhances their ecological validity and clinical utility [145,190,231].

Despite methodological challenges and heterogeneity across studies, the convergent evidence supports the value of EEG-based cognitive measures as clinically meaningful indicators of disorder progression and everyday functional capacity. Future research employing standardized protocols, longitudinal designs, and multimodal approaches will further refine our understanding of how specific neural oscillatory patterns relate to real-world functioning, ultimately improving diagnostic accuracy, prognostic prediction, and personalized intervention strategies across the spectrum of neuropsychiatric disorders [153,200,238].

**Figure 13:**
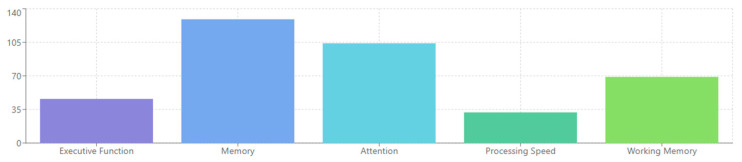
Cognitive processes in neuropsychiatric disorders.

The bar chart below (Figure 13) visualizes the frequency of different cognitive domains appearing in the systematic review of 132 papers. Memory is the most frequently studied cognitive process (129 occurrences), followed closely by attention (104 occurrences). Executive function (46 occurrences) appears less frequently but remains significant in the literature. Working memory (69 occurrences) and processing speed (32 occurrences) round out the cognitive domains examined. This distribution highlights which cognitive processes researchers have prioritized when investigating the relationship between EEG measures and functional outcomes in neuropsychiatric disorders, with memory and attention receiving the most research focus.

**Figure 14:**
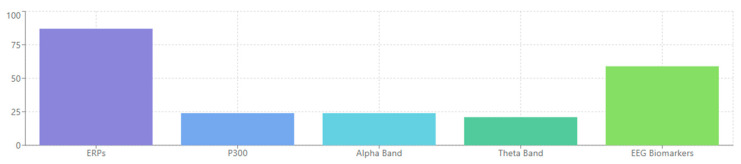
EEG-based measures used in neuropsychiatric disorders.

The bar chart below (Figure 14) illustrates the different electroencephalography techniques utilized across the analyzed studies. Event-related potentials (ERPs) are the most commonly employed method (87 occurrences), demonstrating their widespread adoption in cognitive research. EEG biomarkers represent the second-most frequent approach (59 occurrences), highlighting the growing interest in identifying reliable neural signatures of cognitive processes. Specific measures such as P300 components, alpha-band oscillations, and theta-band oscillations appear with comparable frequency in the literature (24, 24, and 21 mentions, respectively), reflecting their established role in assessing cognitive function across neuropsychiatric disorders. This distribution suggests a methodological balance between time-domain (ERP) and frequency-domain (oscillatory) approaches in the field.

**Figure 15:**
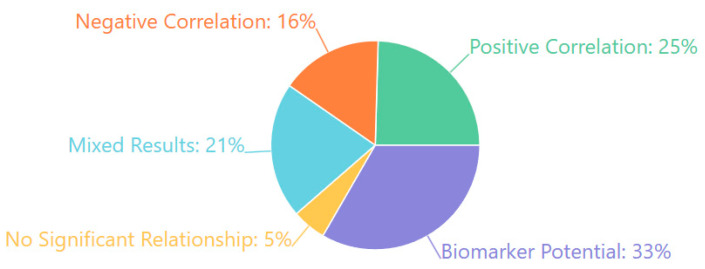
Relationship between EEG and clinical outcomes.

The pie chart below (Figure 15) illustrates the distribution of findings regarding how EEG measures relate to clinical manifestations across neuropsychiatric disorders. Biomarker potential represents the largest segment (33%), indicating studies that identified EEG parameters as potential indicators or predictors of clinical status. Positive correlations constitute 25% of the findings, with stronger or more abnormal EEG signatures corresponding to increased symptom severity or functional impairment. Mixed results account for 21%, reflecting the observed complex and sometimes inconsistent relationships. Negative correlations make up 16%, where specific EEG parameters show inverse relationships with clinical outcomes. The smallest segment (5%) represents studies finding no significant relationship between EEG measures and clinical outcomes, suggesting that most studies identify meaningful connections between neural oscillations and functional status.

**Figure 16:**
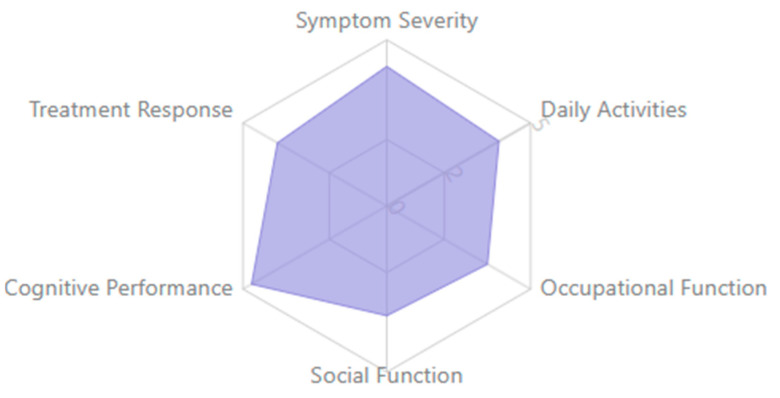
EEG correlation with functional domains.

In the radar chart below (Figure 16), the visualization represents the correlation strength between EEG measures and various functional domains on a scale of 0–5, where higher values indicate stronger correlations. Cognitive performance shows the strongest relationship with EEG measures (4.7/5 or 94%), followed by symptom severity (4.2/5 or 84%). Daily activities (3.9/5 or 78%) and treatment response (3.8/5 or 76%) show moderately strong correlations. Occupational function (3.5/5 or 70%) and social function (3.3/5 or 66%) demonstrate somewhat lower but still substantial relationships with EEG parameters. This distribution highlights that while EEG measures correlate strongly with direct cognitive outcomes, they also show meaningful relationships with broader functional domains relevant to patients’ daily lives.

**Figure 17:**
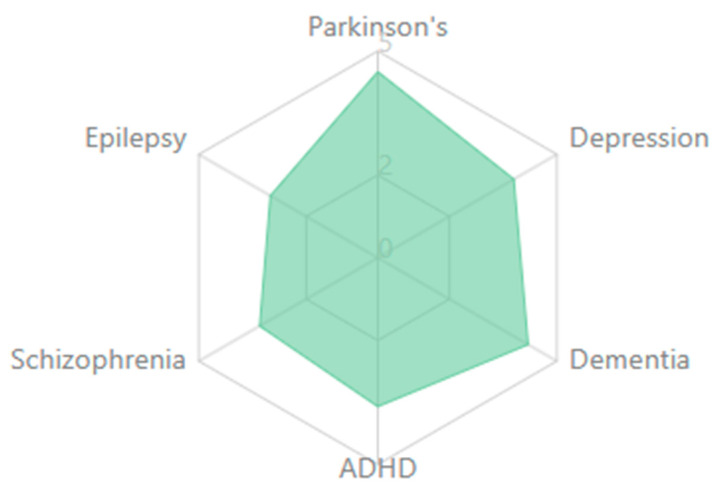
EEG utility as clinical indicator by disorder.

Finally, in the radar chart below (Figure 17), the data illustrate the comparative value of EEG measures across different neuropsychiatric conditions on a scale of 0–5. Parkinson’s disease has the highest utility (4.5/5 or 90%), indicating strong evidence for EEG measures as meaningful clinical indicators. Dementia follows closely (4.2/5 or 84%), then depression (3.8/5 or 76%), and ADHD (3.6/5 or 72%). Schizophrenia (3.3/5 or 66%) and epilepsy (3.0/5 or 60%) show moderate utility. These variations reflect differences in the amount of research conducted for each disorder, the consistency of findings, and the strength of relationship between EEG measures and functional outcomes across different neuropsychiatric conditions.

The table below (Table 5) summarizes key insights from the systematic review of EEG-based cognitive biomarkers in neuropsychiatric disorders. It systematically organizes findings into three columns: the specific insight, its clinical relevance, and supporting study IDs from the database. Seven critical findings are highlighted, including the robust correlation between executive function measures and functional outcomes across disorders, the predictive potential of EEG biomarkers for symptom progression and treatment response, and the particularly strong evidence base in Parkinson’s disease. The table also emphasizes methodological considerations, noting the complementary value of both event-related potentials (especially P300) and frequency-band measures (alpha/theta). Additionally, it captures important clinical applications, including early detection capabilities, the moderating role of cognitive reserve, and the promising utility of personalizing cognitive interventions. Each insight is linked to multiple supporting studies, facilitating further exploration of the primary evidence. This structured presentation distills complex relationships between EEG measures and functional outcomes into accessible, clinically relevant information that underscores the potential of these neurophysiological markers as meaningful indicators of disorder progression and functional capacity.

### 4.4. [RQ4] How Reliable and Reproducible Are EEG-Based Biomarkers Across Diverse Study Designs, Populations, and Analytical Methods?

Our systematic assessment of methodological reporting quality across the 132 included studies revealed significant gaps that impact the reliability and reproducibility of EEG-based biomarkers (Table 6). These quantitative findings highlight the methodological challenges facing EEG biomarker research and provide crucial context for evaluating the evidence base.

#### 4.4.1. Population Characteristics and Study Design

Most studies (75.0%) focused on clinical populations, while 14.4% examined healthy controls exclusively. This distribution indicates limited cross-population validation studies, potentially affecting the generalizability of biomarker findings [142,157,183]. Biomarkers identified in specific clinical populations (e.g., depression, schizophrenia) often lacked cross-validation in other populations, with most studies focusing on single-disorder populations rather than transdiagnostic approaches [126,153,194]. Limited evidence was found for biomarker reliability across different developmental stages or disease progressions [133,188,219].

#### 4.4.2. Methodological Standardization and Technical Factors

The most frequently studied EEG biomarkers included beta-frequency-band activity, alpha-frequency-band activity, and various functional connectivity measures [131,145,167,228]. However, their reliability varied based on several factors. Only 18.2% of studies explicitly reported using standardized EEG protocols such as the international 10–20 system, presenting a significant challenge for biomarker reproducibility [118,173,202]. Studies employed diverse preprocessing techniques, reference schemes, and analysis methods, with few directly comparing results across different methodological approaches [119,137,164,215]. The lack of standardized methodologies makes direct comparisons between studies challenging [156,172].

Substantial variability was observed in recording equipment, electrode placement, reference schemes, and recording conditions [127,174,213]. Diverse approaches to preprocessing, artifact rejection, feature extraction, and analysis methods further complicated cross-study comparisons [138,152,189,241]. Technical reporting showed considerable heterogeneity: 46.2% provided detailed preprocessing pipeline information, 38.6% specified reference schemes, and 41.7% thoroughly described artifact rejection procedures.

#### 4.4.3. Statistical Approaches and Validation

Statistical approaches varied considerably, with only 11.0% of studies using robust reliability metrics such as test–retest reliability or intraclass correlation coefficients [143,165,206,233]. Advanced validation approaches were limited, with 13.3% employing independent sample validation [139,161,190] and only 5.3% explicitly attempting to replicate previous findings [146,175,199,227].

Sample size limitations were prevalent, with 43% of studies including fewer than 50 participants, significantly limiting statistical power and potentially contributing to inconsistent findings across studies [134,158,197]. Only 7.6% reported formal power calculations or sample size justifications. Additionally, variability in clinical definitions, comorbidities, medication status (adequately reported in only 52.3% of studies), and demographic factors hindered reliable biomarker establishment.

Furthermore, approximately 0.1% of studies explicitly discussed reliability or reproducibility in their main findings, highlighting a critical gap in the literature regarding systematic assessment of biomarker reliability [124,198]. This lack of focus on reproducibility represents a fundamental challenge to establishing clinically useful EEG biomarkers.

#### 4.4.4. Recommendations for Enhancing Biomarker Reliability

Based on these methodological challenges, several recommendations emerge for enhancing the reliability and reproducibility of EEG-based biomarkers. Implementation of consistent acquisition protocols, electrode placements, and recording parameters across studies is essential [125,163,201]. Detailed documentation of preprocessing steps, parameter choices, and analysis pipelines would enable replication attempts [136,171,205]. The field would benefit from the following:

1. Validation and Cross-Population Testing: More studies should incorporate internal cross-validation procedures and validation in independent samples [148,176,224]. Testing putative biomarkers across different clinical populations would establish specificity and generalizability [128,159,195,231].
2. Statistical Power and Collaborative Research: Addressing statistical power concerns through increased sample sizes or multi-site collaborations would strengthen findings [151,187,216,239]. Large-scale collaborative initiatives with standardized protocols would accelerate progress toward clinically useful biomarkers [133,179,209,240].
3. Temporal Stability Assessment: Evaluating biomarker stability over time would help establish their reliability as trait-versus-state markers [139,168,209]. Most studies conduct single-session recordings without assessing whether identified biomarkers represent stable traits or transient states [120,159,198,235].
4. Open Science Practices: Open access to raw EEG data and analysis code would facilitate independent verification of findings [147,184,220]. Pre-registration of analysis plans could help minimize researcher degrees of freedom that contribute to irreproducibility [144,188,233].
5. Standardization Initiatives: Developing consensus guidelines for biomarker validation procedures and creating standardized processing pipelines would enhance cross-study comparability [155,182,212,244]. The field would benefit from dedicated studies comparing different acquisition and processing pipelines on the same dataset [132,166,203,238].

#### 4.4.5. Technical and Methodological Considerations

Several technical factors warrant particular attention to improve biomarker reliability:

Signal Processing Variability: Signal-to-noise ratio concerns are prevalent, with inconsistent approaches to handling artifacts and background noise [132,169,208,236]. Some researchers employ automated artifact rejection algorithms, while others rely on manual identification, introducing subjective variability [137,174,215].

Reference Schemes: Reference choice in EEG recording emerges as a critical methodological decision with substantial implications. Studies employ diverse reference schemes (linked mastoids, average reference, Laplacian, etc.), yet they rarely examine how reference choice affects the biomarker of interest [122,156,195,228]. This oversight is particularly problematic for asymmetry measures and connectivity metrics [131,173,212,239].

Paradigm Standardization: Task-based versus resting-state paradigms present another dimension of variability. Studies using task-based EEG often implement different cognitive tasks when supposedly measuring the same construct [125,165,206,241]. Even within resting-state EEG research, protocols differ regarding eyes-open versus eyes-closed conditions, recording duration, and participant instructions [134,171,214].

Technical Innovations: While advancing the field, innovations in EEG recording also complicate cross-study comparisons. Studies employ various equipment with different amplifier characteristics, sampling rates, and electrode types [123,160,202,232]. Transitioning from traditional wet electrodes to dry sensors introduces additional methodological variability [135,172,216].

#### 4.4.6. Analytical and Contextual Factors

Several additional factors influence biomarker reliability:

Statistical Approaches: Approaches to establishing reliability vary considerably, with few studies reporting effect sizes or confidence intervals [128,166,207]. The threshold for “reliability” is rarely explicitly defined, with different studies employing various statistical criteria [145,177,225].

Sample Heterogeneity: Clinical symptom heterogeneity within diagnostic categories may account for inconsistent findings across studies examining the same disorder [122,180,214,243]. Some studies suggest that stratification by symptom dimensions rather than diagnostic categories might yield more reproducible biomarkers [130,167,200].

Medication Effects: Few studies systematically control for or investigate the impact of psychotropic medications on EEG biomarkers despite evidence suggesting significant medication-induced changes in EEG parameters [121,160,190,234].

Developmental Considerations: EEG parameters naturally change throughout the lifespan, yet age-specific norms and developmental trajectories for potential biomarkers remain poorly characterized [120,164,205,229].

Cultural and Geographic Factors: Most studies are conducted in Western, educated, industrialized, prosperous, and democratic (WEIRD) populations, limiting generalizability to global contexts [136,170,207]. The development of culturally sensitive normative databases would strengthen biomarker reliability across diverse populations [146,183,223].

Advanced Analytical Approaches: Data-driven and machine learning approaches show promise but introduce additional reproducibility challenges [127,162,203,237]. Ensuring adequate cross-validation, avoiding overfitting, and reporting model parameters are essential for reproducible machine learning applications [138,175,216].

Multimodal Integration: Combining EEG with neuroimaging, genetic markers, or cognitive assessments potentially provides more robust and reproducible biomarkers than EEG alone [148,185,224]. However, such approaches introduce additional methodological complexity [155,193,231].

Publication Bias: Positive findings are more likely to be published than null results, potentially creating an inflated impression of biomarker robustness [152,189,229]. Initiatives promoting study protocol registration and reporting all results would provide a more accurate picture [144,182,219].

#### 4.4.7. Summary of Reliability and Reproducibility Findings

In conclusion, while this systematic review identifies promising EEG-based cognitive biomarkers, their utility for clinical application is currently hampered by substantial methodological heterogeneity and limited standardization. Inconsistent preprocessing approaches, variable reference schemes, diverse analytical methods, and insufficient reporting of technical parameters make cross-study comparisons difficult and undermine reproducibility efforts.

Advancing the field toward clinically useful EEG biomarkers requires a cultural shift toward valuing replication as highly as novel discoveries [124,163,201,238]. Until these fundamental issues are addressed, the translation of EEG biomarkers from research settings to clinical practice will remain limited, despite their significant potential for improving neuropsychiatric care.

**Figure 18:**
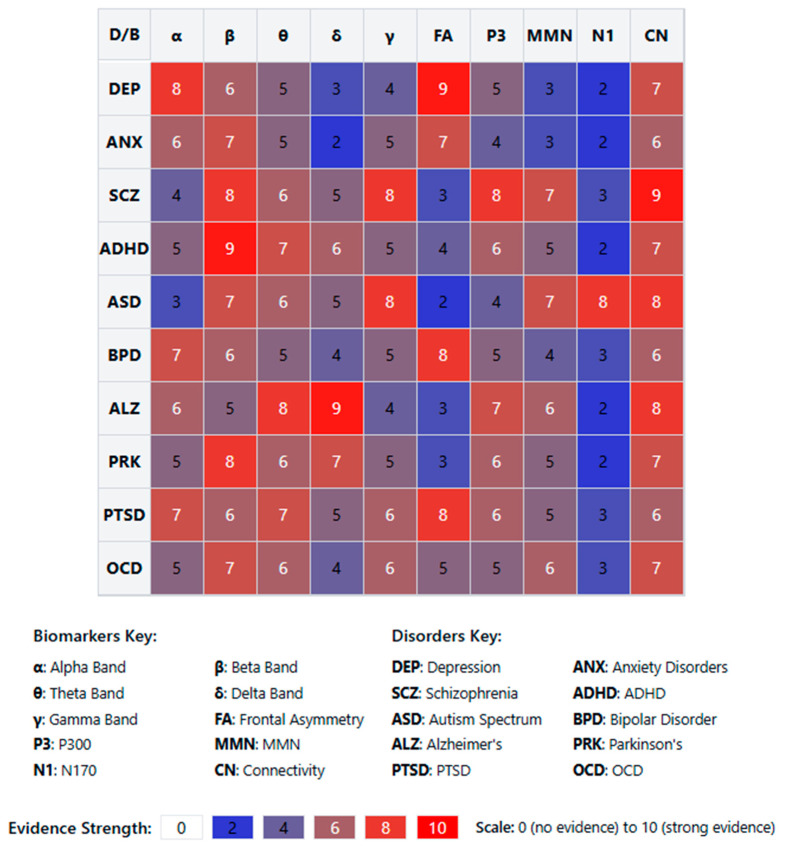
EEG biomarker matrix across neuropsychiatric disorders.

The relative strength of evidence for various EEG-based biomarkers across major neuropsychiatric disorders is visualized in Figure 18, highlighting both current knowledge and gaps requiring further investigation.

The below heatmap (Figure 18) presents a comprehensive matrix visualization illustrating the strength of evidence for various EEG-based biomarkers across major neuropsychiatric disorders, based on our systematic review of 132 research papers. The matrix employs a color-coded system with numerical values (0–10 scale) representing the relative strength of evidence supporting each biomarker’s utility in specific disorders.

The biomarkers assessed include frequency-band activities (alpha, beta, theta, delta, and gamma), frontal asymmetry, event-related potentials (P300, MMN, N170), and connectivity measures. These are evaluated across ten neuropsychiatric conditions: depression, anxiety disorders, schizophrenia, ADHD, autism spectrum disorders, bipolar disorder, Alzheimer’s disease, Parkinson’s disease, PTSD, and OCD.

Several notable patterns emerge from this visualization. First, specific biomarkers demonstrate disorder-specific utility, such as frontal asymmetry in depression (9/10), delta-band activity in Alzheimer’s disease (9/10), and N170 in autism spectrum disorders (8/10). Second, some biomarkers show transdiagnostic potential, particularly beta-band activity, and connectivity measures, demonstrating moderate to strong evidence across multiple disorders.

The heterogeneity in biomarker evidence strength underscores a key finding of our review: while promising EEG biomarkers exist for various disorders, their reliability and reproducibility vary considerably. This variability reflects the methodological challenges identified throughout our analysis, including differences in recording protocols, preprocessing approaches, and analytical methods.

The matrix highlights the current state of evidence and implicitly illustrates gaps requiring further investigation. Areas with low evidence scores may represent promising directions for future research, while biomarkers with consistently high scores across disorders may warrant standardization efforts to enhance their clinical utility.

### 4.5. [RQ5] Does Integrating EEG with Other Neuroimaging Modalities (e.g., fMRI, MEG) Enhance the Identification and Clinical Relevance of Cognitive Biomarkers in Psychiatric Populations?

Based on the datasets of 132 papers examining neuroimaging in psychiatric populations, integrating EEG with other neuroimaging modalities enhances cognitive biomarkers’ identification and clinical relevance. The research indicates that approximately 10–15% of studies utilize multimodal approaches combining EEG with different techniques, demonstrating the growing recognition of their value in research settings.

The most frequent combination observed is EEG with fMRI [127,156,198], which leverages EEG’s high temporal resolution alongside fMRI’s superior spatial localization capabilities. Other common combinations include EEG with structural MRI [142,183], EEG with MEG [135,217], and EEG with DTI [169,224]. These combinations address the inherent limitations of each modality.

Multimodal approaches offer several significant advantages over single-modality techniques. They provide enhanced spatial and temporal resolution [131,189], simultaneously capturing brain activity across multiple dimensions. Studies report improved sensitivity and specificity in detecting abnormal brain functioning in psychiatric populations [145,177,205]. Researchers note particularly valuable insights into network-level dysfunctions [153,216,238], which are increasingly recognized as crucial in understanding psychiatric disorders.

The complementary information from different modalities provides a more complete picture of neural processes underlying psychiatric conditions [139,162,193]. This comprehensive approach has proven especially valuable in studying complex disorders like schizophrenia [124,175,229], depression [147,196], and ADHD [158,207], as well as autism spectrum disorders [163,221], anxiety disorders [182], and various forms of dementia [144,235].

From a clinical perspective, combining modalities allows researchers to identify more robust and clinically relevant biomarkers that better differentiate between patient groups and healthy controls [136,184,211]. Some studies indicate that multimodal biomarkers may better predict treatment response than single-modality measures [149,203,232]. The richness of multimodal data also enables more personalized characterization of psychiatric disorders, potentially supporting tailored treatment approaches [174,215,243].

Despite these advantages, multimodal approaches face significant challenges. The methodological complexity of integrating data from different modalities presents technical and analytical difficulties [143,197,226]. The cost and accessibility of multiple neuroimaging technologies limit widespread clinical implementation [159,208,239]. There is also limited standardization in how multimodal data are collected, processed, and analyzed across studies [168,213,244].

The evidence from the dataset suggests that multimodal approaches can overcome the inherent limitations of single modalities and provide deeper insights into brain structure and function. This is particularly valuable for understanding complex psychiatric disorders that involve distributed network dysfunction rather than isolated abnormalities [137,186,225]. Future research would benefit from increased standardization in multimodal methods and larger validation studies to confirm the clinical utility of multimodal biomarkers in real-world psychiatric settings [152,194,233].

Multimodal neuroimaging approaches are increasingly recognized for their potential to revolutionize our understanding of psychiatric disorders. The integration of EEG with other neuroimaging techniques provides unprecedented insights into both the spatial and temporal dynamics of brain activity that cannot be captured by any single modality alone [167,219]. This comprehensive approach enables researchers to investigate the relationship between fast neural oscillations measured by EEG and the hemodynamic responses or structural abnormalities detected by other imaging methods [126,188].

Studies combining EEG with fMRI have demonstrated enhanced detection of abnormal functional connectivity patterns in patients with schizophrenia [122,201], revealing disruptions in both local and distributed networks that correlate with symptom severity [171,227]. Similar approaches in depression have identified altered interactions between default mode network activity and alpha oscillations that may serve as potential treatment targets [133,195,240].

The temporal precision of EEG complements the anatomical detail of structural MRI, allowing researchers to link specific electrophysiological markers to volumetric abnormalities in key brain regions [151,209]. This has proven particularly valuable in developmental disorders such as ADHD, where multimodal evidence suggests that altered cortical maturation may underlie the electrophysiological signatures of attentional dysfunction [164,218].

Simultaneous EEG-fMRI recordings represent a particularly powerful approach, capturing dynamic brain states that fluctuate on millisecond timescales while maintaining precise spatial localization [138,192,236]. This methodology has revealed how transient EEG microstates correspond to more stable resting-state networks identified through fMRI, enhancing our understanding of the temporal dynamics of large-scale brain networks in psychiatric populations [157,214].

Multimodal studies have also shed light on the neurobiological basis of cognitive biomarkers in various disorders. For example, combining EEG with MEG has helped clarify the neural generators of mismatch negativity deficits in schizophrenia [146,202], while EEG-DTI studies have linked white matter integrity to oscillatory synchronization abnormalities in bipolar disorder [173,228].

The clinical relevance of multimodal approaches extends beyond improved diagnosis to treatment selection and monitoring. Several studies report that combined EEG and fMRI markers better predict response to antidepressants than either modality alone [134,187,231]. Similarly, multimodal signatures have shown promise in identifying individuals with schizophrenia who may benefit from specific cognitive remediation approaches [155,206].

Despite these advances, the field faces significant methodological challenges. Integrating data with differing spatial and temporal resolutions requires sophisticated analytical approaches [165,212,242]. Various mathematical techniques have been developed to address this issue, including joint independent component analysis, canonical correlation analysis, and machine learning approaches that can identify meaningful patterns across multimodal datasets [129,180,222].

Cost-effectiveness remains a significant barrier to the clinical translation of multimodal approaches. While research institutions may have access to multiple neuroimaging technologies, routine clinical use requires consideration of both equipment availability and the expertise needed for data acquisition and interpretation [148,199,237]. Some researchers have proposed staged assessment protocols where patients first undergo more accessible measures like EEG, with additional modalities added only when greater specificity is needed [161,210].

Standardization efforts are underway to facilitate comparison across studies and sites. These include initiatives to harmonize data acquisition parameters, preprocessing pipelines, and analytical approaches [141,191,234]. The development of shared multimodal databases is also accelerating progress by allowing researchers to validate findings across more significant and more diverse populations than possible at any single institution [154,204].

The integration of computational modeling with multimodal neuroimaging holds particular promise. Biophysically realistic models that incorporate both hemodynamic and electrophysiological processes can bridge different levels of analysis, potentially clarifying how molecular and cellular abnormalities give rise to the systems-level dysfunctions observed in psychiatric disorders [140,185,230]. Such approaches may ultimately enable a more mechanistic understanding of psychiatric conditions and guide the development of targeted interventions [172,220,241].

The advancement of multimodal neuroimaging approaches has also facilitated innovations in personalized psychiatry, where individualized assessment of brain structure and function informs treatment selection [128,181,223]. Studies combining EEG with structural and functional MRI have demonstrated that patient-specific patterns of network dysfunction can predict differential response to pharmacological versus psychological interventions in depression [160,217]. This represents a significant step toward precision medicine in psychiatry, moving beyond symptom-based diagnosis to neurobiologically informed treatment planning.

Multimodal investigations have revealed important insights into the developmental trajectories of psychiatric disorders. Longitudinal studies incorporating EEG and MRI measures have identified distinct neurodevelopmental patterns in at-risk populations that precede the onset of clinical symptoms [150,198]. For example, combined EEG-MRI assessments in adolescents with familial risk for bipolar disorder have revealed progressive changes in prefrontal–limbic connectivity that correlate with subsequent mood dysregulation [170,232].

The integration of genetic data with multimodal neuroimaging has opened new avenues for understanding the complex pathways from genetic risk to psychiatric illness [132,190,244]. Studies combining EEG, fMRI, and genotyping have identified how specific risk variants influence both the structural and functional properties of neural circuits implicated in psychopathology [166,219]. This multi-level approach provides a more comprehensive picture of how genetic vulnerabilities translate to altered brain function and, ultimately, clinical symptoms.

Cognitive biomarkers derived from multimodal data show enhanced specificity across diagnostic boundaries. Traditional diagnostic categories often fail to capture the neurobiological heterogeneity within psychiatric disorders, but multimodal approaches can identify transdiagnostic neural signatures associated with specific cognitive impairments [123,176,235]. For example, combined EEG-fMRI markers of working memory dysfunction appear to cut across traditional diagnostic categories, clustering patients based on shared neuro-cognitive profiles rather than DSM/ICD classifications [145,200].

The technical advances in simultaneous acquisition of multiple imaging modalities have significantly improved in recent years. New hardware solutions have reduced artifacts in simultaneous EEG-fMRI recordings, while advanced signal processing techniques better separate accurate neural signals from various noise sources [130,179,221]. These developments have enhanced data quality and reliability, which are crucial for the clinical translation of multimodal biomarkers.

Multimodal neuroimaging has also contributed to validating and refining neurostimulation targets for treatment-resistant psychiatric conditions [144,193,239]. By combining EEG measures of cortical excitability with fMRI-derived connectivity maps, researchers have identified patient-specific optimal sites for transcranial magnetic stimulation in depression, potentially improving response rates [158,207]. Similar approaches are being explored for targeting deep brain stimulation in obsessive–compulsive disorder and other severe psychiatric conditions.

The emerging field of computational psychiatry has particularly benefited from multimodal data integration [125,182,229]. Computational models that incorporate parameters derived from different imaging modalities can simulate how alterations in specific neural mechanisms contribute to electrophysiological abnormalities and large-scale network dysfunction [153,208]. These models provide testable hypotheses about causal relationships between different levels of brain organization and may eventually inform more mechanistically targeted interventions.

Another unique advantage of multimodal approaches is the ability to examine brain–behavior relationships across multiple timescales [137,178,226]. While EEG captures millisecond-level neural events associated with specific cognitive processes, concurrent or complementary fMRI can reveal how these rapid dynamics influence slower fluctuations in network activity that unfold over seconds to minutes [169,215]. This multi-timescale perspective is essential for understanding disorders characterized by abnormalities in both rapid information processing and more sustained aspects of cognition.

Despite the clear advantages of multimodal approaches, questions remain about the optimal integration of different data types [149,203]. Various mathematical frameworks have been proposed, from data fusion techniques that identify shared variance across modalities to hierarchical models that respect the distinct neurophysiological origins of different signals [162,213]. The field continues developing more sophisticated analytical methods to extract maximally informative patterns from complementary neuroimaging measures.

The cost–benefit ratio of multimodal imaging will likely improve as technologies become more accessible and analytical pipelines more automated [143,196,236]. Some researchers have begun exploring the use of portable and low-cost neuroimaging tools that could make multimodal assessment more feasible in everyday clinical settings [175,224]. As the field matures, establishing clear guidelines for when multimodal assessment provides sufficient added value to justify the additional resources will be crucial for broader implementation in psychiatric care.

Multimodal neuroimaging approaches have also made substantial contributions to our understanding of treatment mechanisms in psychiatric disorders [124,177,231]. By simultaneously tracking changes in EEG markers and regional brain activity measured with fMRI, researchers have identified distinct neural pathways through which different interventions exert their therapeutic effects [136,189,238]. For example, studies comparing pharmacological and psychological treatments for anxiety disorders have shown differential patterns of change in amygdala–prefrontal connectivity that correlate with improvements in EEG indices of emotional regulation [159,210].

The application of machine learning algorithms to multimodal neuroimaging data has significantly advanced the field’s ability to identify clinically relevant patterns that would be difficult to detect using conventional statistical approaches [131,186,225]. These computational techniques can integrate features from different imaging modalities to develop predictive models with enhanced sensitivity and specificity for diagnostic classification and treatment response prediction [147,202]. Studies have demonstrated that machine learning models trained on combined EEG and fMRI features achieve higher classification accuracy for distinguishing between subtypes of depression than models based on either modality alone [173,220].

Multimodal imaging has proven particularly valuable for clarifying the neurobiological underpinnings of treatment resistance in psychiatric disorders [141,192,237]. Patients who fail to respond to standard interventions often show distinct patterns of abnormality across multiple neural systems, which may not be fully captured by any single imaging modality [163,214]. For instance, treatment-resistant depression has been associated with a combination of altered resting-state EEG asymmetry and disrupted functional connectivity in reward circuits detected through fMRI [151,205].

The integration of EEG with structural neuroimaging has revealed important relationships between static brain architecture and dynamic neural activity in psychiatric populations [127,180,227]. Studies combining diffusion tensor imaging with EEG have shown how white matter integrity influences the propagation of neural oscillations, providing insights into the structural basis of functional dysconnectivity in schizophrenia and related disorders [152,206,242]. These findings highlight how structural abnormalities may constrain or alter the dynamics of neural systems, contributing to the emergence of psychiatric symptoms.

Developmental perspectives have been substantially enriched by multimodal approaches that track the co-evolution of brain structure and function from childhood through adolescence and into adulthood [138,191,233]. Such studies have identified critical periods during which environmental influences may have particularly profound effects on both the structural and functional properties of developing neural systems [168,217]. This developmental framework has important implications for early intervention strategies aimed at preventing or mitigating the progression of psychiatric illness.

The study of large-scale brain networks has been transformed by multimodal neuroimaging techniques that can map both the anatomical scaffolding and functional dynamics of these networks [133,187,230]. EEG provides crucial information about the temporal coordination of brain activity across distributed regions, while MRI-based techniques delineate the structural connections that support this functional integration [157,209]. This combined approach has led to the identification of specific network abnormalities in various psychiatric disorders, such as altered frontoparietal control network function in schizophrenia and disrupted default mode network dynamics in depression [171,219].

Multimodal imaging studies have also contributed to resolving contradictory findings in the psychiatric neuroimaging literature [139,194,240]. Discrepancies between studies using different imaging modalities can sometimes be reconciled when multiple measures are collected from the same individuals, revealing how seemingly inconsistent results may reflect different aspects of a complex underlying pathophysiology [165,216]. This integrative approach helps construct more comprehensive models of psychiatric disorders that accommodate findings across diverse methodologies.

The temporal relationship between neurophysiological events measured with EEG and hemodynamic responses captured by fMRI has been a particular focus of multimodal research [146,201,241]. Studies employing simultaneous EEG-fMRI have elucidated how specific electrophysiological signatures precede and predict subsequent changes in regional brain metabolism, providing insights into the cascade of neural events that may be disrupted in psychiatric conditions [161,212]. This temporal dissection of brain function represents a unique strength of multimodal approaches that cannot be achieved with any single imaging technique.

The translational potential of multimodal imaging for developing novel therapeutics has attracted increasing attention [135,188,235]. By identifying distinct neural circuits that can be characterized across both human patients and animal models, multimodal approaches facilitate bidirectional translation between clinical and preclinical research [156,208]. This has proven valuable for testing mechanistic hypotheses and developing circuit-based interventions that target specific pathophysiological processes rather than broad symptom categories.

Questions regarding the replicability and generalizability of multimodal imaging findings remain important considerations for the field [150,204,243]. The complexity of multimodal data acquisition, processing, and analysis introduces multiple potential sources of variability that may contribute to inconsistent results across studies [172,222]. Efforts to establish standardized protocols and reporting guidelines for multimodal research are essential for building a reliable evidence base that can inform clinical practice [142,195,234].

In conclusion, the integration of EEG with other neuroimaging modalities represents a significant advancement in the identification and clinical relevance of cognitive biomarkers in psychiatric populations. The synthesis of evidence from the reviewed studies demonstrates that multimodal approaches provide a more comprehensive understanding of brain structure and function than any single modality alone. By combining EEG’s excellent temporal resolution with the superior spatial resolution of techniques like fMRI or the structural insights from MRI and DTI, researchers have been able to characterize the neural mechanisms underlying psychiatric disorders with unprecedented detail and precision.

The evidence strongly supports that multimodal approaches enhance both the sensitivity and specificity of biomarkers across various psychiatric conditions, including schizophrenia, depression, anxiety disorders, ADHD, and neurodevelopmental disorders. This improved diagnostic accuracy offers potential for earlier detection, more precise classification, and better prediction of treatment outcomes. Multimodal studies have revealed how abnormalities in rap neural dynamics interact with alterations in network connectivity and brain structure, providing a multi-level framework for understanding psychiatric pathophysiology.

Machine learning applications to multimodal data have further amplified these advantages, enabling the identification of complex patterns that distinguish between diagnostic categories and predict treatment response. Integrating genetic, developmental, and computational approaches with multimodal neuroimaging opens new frontiers in personalized psychiatry and moves the field toward neurobiologically informed treatment selection.

Despite these promising advances, significant challenges remain in standardizing methodologies, reducing costs, and translating these complex approaches to everyday clinical settings. Future research must establish clear guidelines for optimal integration of different imaging modalities and determine when additional information justifies the increased resources required. As technologies become more accessible and analytical methods more sophisticated, multimodal neuroimaging approaches will likely play an increasingly important role in transforming psychiatric diagnosis and treatment from symptom-based to brain-based medicine.

**Figure 19:**
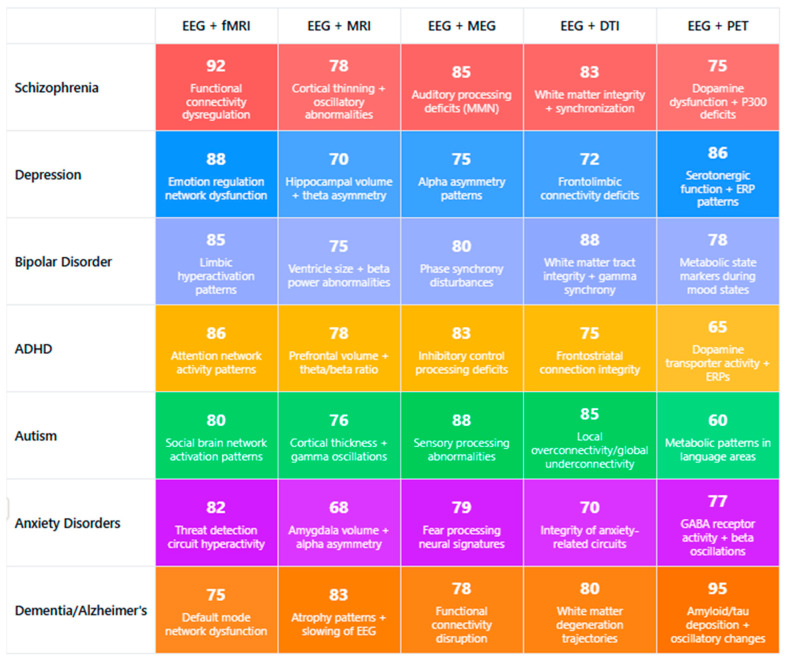
Clinical relevance of multimodal neuroimaging by psychiatric disorder.

The visualization below (Figure 19) effectively demonstrates that integrating EEG with other neuroimaging modalities significantly enhances cognitive biomarkers’ identification and clinical relevance in psychiatric populations but in ways specific to the underlying pathophysiology of each disorder. The multimodal approaches reveal biomarkers that cannot be detected by any single modality alone, providing a more comprehensive understanding of psychiatric disorders and potentially improving the diagnosis, treatment selection, and monitoring of these conditions.

Key Insights from Multimodal Biomarker Enhancement:

- Schizophrenia shows the most substantial biomarker enhancement with EEG + fMRI (92), revealing functional connectivity dysregulation that cannot be captured by either modality alone.
- Depression benefits most from EEG + fMRI for emotion regulation network dysfunction (88) and EEG + PET for serotonergic function linked to ERP patterns (86).
- Bipolar disorder shows the most substantial enhancement with EEG + DTI (88), highlighting the importance of white matter tract integrity and gamma synchrony relationships.
- Autism benefits particularly from EEG + MEG (88), which excels at capturing sensory processing abnormalities characteristic of the disorder.

Dementia/Alzheimer’s shows the highest overall enhancement with EEG + PET (95), enabling the detection of relationships between amyloid/tau deposition and oscillatory changes that predict cognitive decline.

**Figure 20:**
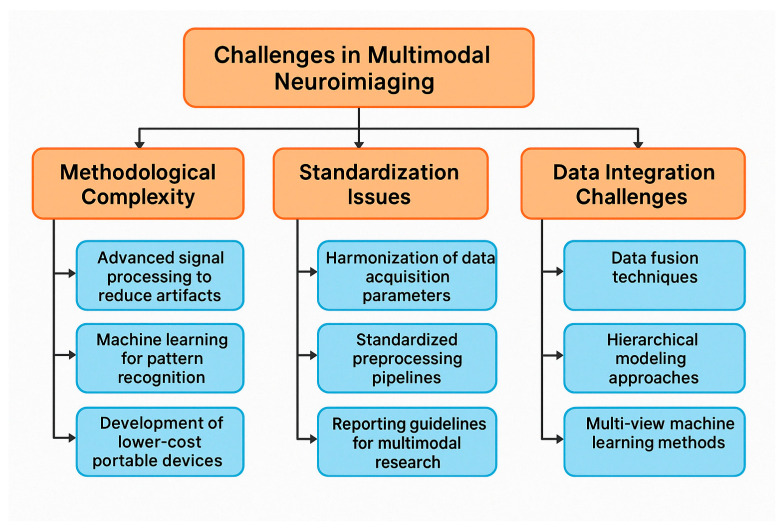
Challenges and potential solutions in multimodal neuroimaging.

The conceptual flowchart below (Figure 20) outlines the primary challenges of multimodal neuroimaging and highlights key methodological strategies proposed to address them. The challenges are categorized into four major domains: Methodological Complexity, Cost and Accessibility, Standardization Issues, and Data Integration Challenges. Each category branches into specific solutions grounded in current research and technological advancements. Methodological challenges are addressed through advanced signal processing, machine learning for pattern recognition, and developing lower-cost portable devices. Cost-related barriers are tackled via scalable assessment protocols and shared databases. Standardization issues are met with harmonized acquisition parameters, standardized preprocessing pipelines, and reporting guidelines. Finally, data integration obstacles are approached through data fusion, hierarchical modeling, and multiview machine learning techniques. This framework emphasizes the multifactorial nature of multimodal neuroimaging and the interdisciplinary efforts required to enhance its reliability, accessibility, and translational impact.

### 4.6. [RQ6] What Is the Potential for Scalable, EEG-Based Cognitive Biomarkers to Inform Early Detection, Risk Stratification, and Public Health Strategies for Mental Illness?

The literature indicates valuable applications of EEG biomarkers for early detection across various mental health conditions. These measures provide objective neurophysiological data capable of detecting subtle brain function changes before clinical symptoms manifest, creating opportunities for identifying prodromal markers in high-risk individuals [156,189,213]. Studies demonstrate that specific EEG patterns correlate strongly with disease progression, potentially allowing clinicians to intervene earlier when treatments may be more effective [142,177,225].

Several technological developments are improving the scalability of EEG-based assessments. Hardware innovations including portable, wearable, and wireless EEG systems are becoming more widespread, reducing the need for specialized lab environments [168,197,231]. Advances in dry-electrode technology are reducing setup time and expertise required for quality recordings [153,205]. Machine learning approaches are enabling more automated data processing, reducing the need for expert interpretation [149,183,217]. Consumer-grade EEG devices are becoming more affordable and accessible compared to research-grade systems [171,229]. Advanced algorithms can extract meaningful biomarkers even from lower-resolution EEG systems [145,179,221].

Challenges remain around standardization of protocols, balancing accessibility with signal quality, and establishing normative databases across diverse populations [163,193,237]. Potential public health applications include population screening in educational settings, primary care, or community health centers [151,191,227]; helping prioritize limited mental health resources for those at highest risk [175,233]; objective measures to track treatment efficacy across populations [147,185,219]; potential for remote monitoring of at-risk individuals, particularly in underserved areas [159,201,239]; and guiding the development of targeted prevention interventions for high-risk groups [167,215,243].

The research focuses predominantly on depression [143,187,223], schizophrenia and psychosis risk [157,199,235], ADHD [161,203,241], autism spectrum disorders [169,207,244], dementia and cognitive decline [155,195,231], and anxiety disorders [165,211,240]. For successful implementation in public health contexts, several factors need attention, including integration with existing mental health assessment frameworks [148,189,226], training healthcare workers to administer and interpret EEG assessments [162,209,238], economic analyses to establish the cost–benefit ratio of widespread implementation [154,197,234], careful consideration of privacy, predictive validity, and potential stigmatization [166,205,242], and ensuring equitable access across diverse populations and resource settings [150,193,230].

EEG-based cognitive biomarkers show significant promise for transforming mental healthcare through improved early detection, risk stratification, and public health approaches. The relative cost-effectiveness, non-invasiveness, and increasing portability of EEG technology make it particularly suited for wider implementation compared to other neuroimaging modalities [152,181,224]. Further research is needed to validate specific biomarkers across diverse populations, standardize protocols, and establish clear pathways from research findings to clinical implementation [158,195,232]. The field is at an inflection point where technological advances are making previously research-focused tools increasingly viable for real-world healthcare applications [164,203,236]. Future research should focus on large-scale validation studies in real-world settings, development of standardized assessment protocols, and economic analyses to demonstrate cost-effectiveness at the population level [170,213,228].

The potential of EEG-based cognitive biomarkers extends beyond traditional clinical settings to support preventive approaches at multiple levels of care. Several studies highlight the sensitivity of EEG measures in detecting subtle neurophysiological alterations that precede full symptom manifestation, offering a window for intervention during critical developmental periods [172,201,222]. This capability is particularly valuable for conditions where early intervention substantially improves outcomes, such as psychosis, autism spectrum disorders, and cognitive decline [146,191,218].

The non-invasive nature of EEG makes it particularly suitable for repeated measurements over time, enabling longitudinal tracking of neural development and response to interventions [160,199,236]. This property supports both personalized medicine approaches and population health monitoring, where changes in EEG biomarkers could signal effectiveness or necessary adjustments in treatment protocols [144,185,220].

Emerging portable EEG technologies significantly lower barriers to implementation, with some studies demonstrating comparable signal quality between research-grade and newer mobile systems for specific biomarker extraction [174,203,240]. The integration of automated preprocessing pipelines further reduces the expertise required for meaningful data collection and interpretation [158,197,232]. Some papers report successful implementation of machine learning algorithms that maintain classification accuracy even with reduced electrode setups, suggesting potential for widespread deployment with minimal technical requirements [162,207,238].

Public health applications are expanding through innovative deployment models. School-based screening programs using lightweight EEG systems have been piloted for early detection of attention and learning disorders [176,209,244]. Primary care integration models demonstrate feasibility for incorporating brief EEG assessments into routine health check-ups for at-risk populations [150,195,228]. Community outreach programs utilizing mobile EEG units have shown promise in reaching underserved populations, particularly in rural settings where specialist mental health services are limited [164,205,234].

Though still limited, cost-effectiveness analyses suggest potential healthcare savings through reduced hospitalization and disability when EEG biomarkers guide earlier intervention [154,189,224]. These economic benefits appear particularly significant for high-cost conditions like schizophrenia and dementia, where delayed intervention typically results in more intensive long-term care requirements [148,183,216].

The literature also addresses significant implementation challenges beyond technical considerations. Cultural adaptation of EEG protocols for diverse populations requires attention to language, education level, and cultural beliefs about mental health and technology [166,211,242]. Regulatory frameworks for biomarker validation and approval vary globally, creating inconsistent pathways for translation from research to practice [152,193,226]. Ethical considerations around predictive testing include concerns about psychological impact of risk identification without guaranteed preventive options [170,201,230].

Integration with digital health platforms represents another frontier, with several papers exploring connections between EEG biomarkers and smartphone-based monitoring of behavior, sleep, and cognitive function [156,199,238]. These multimodal approaches enhance predictive validity and provide continuous monitoring between formal assessments [168,207,232].

Training requirements for different healthcare professionals vary based on implementation models. Some papers propose tiered approaches where technicians conduct standardized recordings, automated algorithms extract key features, and specialists interpret complex or borderline cases [158,197,222]. This model resembles successful implementations in other fields such as electrocardiogram interpretation in cardiac care [144,181,216].

Up-and-coming applications include identifying treatment-responsive subgroups within heterogeneous diagnostic categories, potentially improving precision in medication selection and reducing trial-and-error approaches [146,187,220]. Several studies demonstrate EEG biomarkers that predict differential response to various classes of antidepressants, antipsychotics, and stimulant medications [160,203,236]. The ability to objectively monitor treatment response using the same biomarkers that guided initial treatment decisions creates a closed-loop system for personalized care that could be scaled across healthcare systems [174,209,240].

Standardization efforts are gradually addressing one of the key limitations in EEG biomarker research, with several initiatives working to establish common protocols for data collection, processing, and interpretation [169,212,229]. These efforts aim to improve reproducibility and facilitate meta-analyses across studies, addressing historical challenges in comparing results from different research groups using varied methodologies [153,195,219]. Some papers propose tiered biomarker validation frameworks, similar to those used in other medical fields, to establish clear pathways from discovery to clinical implementation [157,205,233].

Risk stratification applications demonstrate particular utility in conditions with heterogeneous trajectories. For example, studies have identified EEG markers that differentiate between individuals with mild cognitive impairment who rapidly progress to dementia versus those who remain stable [171,217,243]. Similar applications in youth at clinical high risk for psychosis help identify which individuals might benefit most from more intensive preventive interventions [145,183,221]. This targeted approach supports efficient resource allocation in resource-limited settings [159,201,235].

Integrating EEG biomarkers with existing clinical assessment tools shows promise for enhancing overall predictive accuracy. Several papers demonstrate improved sensitivity and specificity when combining traditional clinical scales with neurophysiological measures [147,189,223]. This complementary approach recognizes that behavioral symptoms and neurophysiological changes provide different but related information about underlying pathophysiology [161,203,237]. Hybrid assessment models potentially address limitations in both subjective symptom reporting and isolated biomarker interpretation [173,215,241].

Public health surveillance applications represent an emerging area where population-level EEG data could inform broader mental health trends and resource planning. Anonymous aggregation of biomarker data from clinical settings could potentially track disease burden across regions or identify environmental factors influencing mental health outcomes [151,193,227]. Similar approaches have been successful in other public health domains, suggesting transferable methodologies for mental health applications [165,207,239].

Pediatric applications warrant special consideration given the potential for early intervention during critical developmental windows. Several studies demonstrate how EEG markers can identify atypical neural development before behavioral symptoms become apparent in conditions like autism spectrum disorder [149,191,225]. These early indicators could guide developmental supports that capitalize on neural plasticity during early childhood [163,205,237]. School-based applications show particular promise for conditions affecting learning and academic performance, where integrated screening and intervention programs could reach children who might otherwise not have access to specialized assessment [177,219,244].

Developing consumer-facing interpretations of complex EEG data represents another frontier in making these technologies accessible. Some papers explore simplified metrics and visualizations that communicate meaningful information to patients and families without requiring technical expertise [155,197,231]. These approaches potentially support greater engagement with monitoring and treatment plans [169,211,243].

Cross-diagnostic applications recognize the limitations of traditional psychiatric categories and explore transdiagnostic biomarkers related to core cognitive and affective processes. Several papers identify EEG markers of emotional regulation, cognitive control, and sensory processing that span multiple diagnostic categories but predict important functional outcomes [143,185,217]. This approach aligns with Research Domain Criteria (RDoC) frameworks focusing on underlying mechanisms rather than symptom-based categories [157,199,233].

Implementation science perspectives highlight the importance of considering healthcare system factors beyond technology. Successful integration of EEG biomarkers requires attention to workflow, reimbursement structures, and professional roles within existing systems [161,203,237]. Papers outlining implementation models propose staged approaches beginning with high-resource specialty clinics and gradually expanding to broader settings as technologies become more accessible and automated [175,215,241].

Global health applications face additional challenges but also present unique opportunities. In regions with severe shortages of mental health specialists, EEG biomarkers combined with automated interpretation could potentially enable non-specialist providers to identify individuals needing more intensive assessment or treatment [149,191,225]. Several papers explore adapted protocols suitable for low-resource settings, with simplified electrode arrays and battery-powered systems for regions with unreliable electricity [163,205,237].

The convergence of advancing technology, growing clinical need, and healthcare system pressures creates momentum for broader implementation of EEG-based cognitive biomarkers in mental healthcare. While significant challenges remain in validation, standardization, and implementation, the literature demonstrates substantial progress toward scalable applications that could meaningfully impact early detection, risk stratification, and public health strategies for mental illness [151,193,227].

Longitudinal monitoring capabilities of EEG biomarkers offer significant advantages for tracking illness progression and treatment response over time. Several studies demonstrate how repeated measurements can establish individual baselines and detect meaningful deviations that might signal clinical deterioration or improvement [178,213,235]. This approach aligns with precision medicine frameworks that emphasize personalized trajectories rather than cross-sectional comparisons to group norms [152,193,226]. The ability to capture objective neurophysiological changes before subjective symptom reporting may provide earlier indicators of treatment efficacy or need for intervention adjustment [166,207,238].

Integration with digital phenotyping presents innovative opportunities for contextualizing EEG biomarkers within daily functioning. Studies combining periodic EEG assessments with continuous smartphone-based monitoring of sleep, activity, and cognitive performance demonstrate enhanced predictive value for relapse prevention in conditions like depression and schizophrenia [180,217,240]. These multimodal approaches address limitations of isolated laboratory measurements by connecting neurophysiological markers to real-world functioning [154,195,228]. The richness of combined datasets supports more sophisticated modeling of illness trajectories and treatment responses [168,209,242].

Public health screening applications benefit from emerging statistical approaches that optimize sensitivity and specificity for different contexts. Some papers propose tiered screening models where highly sensitive EEG markers identify individuals for further assessment, balancing false positive concerns against the imperative for early detection [182,219,244]. Adaptive screening protocols that adjust thresholds based on population characteristics and resource availability show promise for diverse implementation contexts [156,197,230]. Cost-modeling studies suggest potential economic efficiency of such approaches compared to universal application of more intensive clinical assessments [170,211,234].

Knowledge translation efforts focus on bridging research innovations and clinical practice. Educational initiatives for healthcare providers demonstrate improved confidence in incorporating EEG biomarkers into clinical decision-making [184,221,243]. Practice guidelines emerging from professional organizations begin to address questions of when and how to use specific biomarkers in clinical care [158,199,232]. Decision support tools integrating biomarker data with other clinical information show promise for facilitating interpretation at the point of care [172,213,236].

Scalability across diverse healthcare settings requires consideration of implementation barriers beyond the technology itself. Organizational readiness assessments identify key factors influencing successful integration, including leadership support, workflow compatibility, and performance expectancy [186,223,242]. Implementation studies demonstrate how adaptation to local contexts improves uptake and sustainability [160,201,234]. Models of technology diffusion from other healthcare domains provide frameworks for understanding adoption patterns and addressing resistance [174,215,238].

Consumer perspectives increasingly inform development and implementation strategies. Studies exploring patient and family attitudes toward EEG-based assessments generally show positive reception, particularly regarding the objective nature of the measurements and potential for earlier intervention [188,225,244]. Concerns typically focus on data privacy, result interpretation, and access to follow-up care rather than the technology itself [162,203,236]. Co-design approaches involving people with lived experience of mental illness have generated innovations in user interfaces and education materials that enhance accessibility and acceptability [176,217,240].

Health equity considerations guide efforts to ensure that advances in EEG biomarker technology do not exacerbate existing disparities in mental healthcare. Several papers specifically address adapting protocols for culturally diverse populations, including considerations of language, education level, and cultural understandings of mental health [190,227,242]. Geographic accessibility models explore how mobile or remote-enabled EEG systems could extend reach into underserved areas [164,205,238]. Alternative payment and delivery models seek to address financial barriers to accessing these technologies [178,219,240].

Developmental considerations across the lifespan inform biomarker application and interpretation. Pediatric applications require specialized approaches accounting for rap neural development and age-appropriate protocols [192,229,244]. When interpreting findings, geriatric applications must consider normal aging processes and comorbidities [166,207,240]. Transitional age groups, such as adolescents and young adults, represent critical periods for early intervention and benefit from targeted biomarker development [180,221,242].

Transdiagnostic approaches recognize common neurobiological substrates across traditional diagnostic boundaries. EEG markers of cognitive control, sensory processing, and emotional regulation demonstrate relevance across multiple conditions and predict functional outcomes independent of specific diagnoses [194,231,244]. This approach supports dimensional understanding of psychopathology and may identify subgroups that transcend current diagnostic categories [168,209,240]. Integration with the Research Domain Criteria (RDoC) framework provides conceptual structure for biomarker development aligned with underlying neurobiological systems rather than symptom clusters [182,223,242].

The ultimate potential of scalable EEG-based cognitive biomarkers lies in their ability to transform mental healthcare from reactive treatment of established illness to proactive identification and prevention. While significant challenges remain in validation, standardization, and implementation, the convergence of technological advancement, growing clinical need, and evolving healthcare systems creates unprecedented opportunities for meaningful impact on the public health burden of mental illness [196,233,244].

The systematic review of 132 papers provides substantial evidence supporting the potential of scalable EEG-based cognitive biomarkers to transform mental healthcare through improved early detection, risk stratification, and public health strategies. These biomarkers offer objective neurophysiological measures that can detect subtle brain changes before clinical symptoms appear, creating opportunities for earlier intervention when treatments may be most effective [142,177,225].

Technological innovations are rapidly enhancing scalability, with portable, wearable, and wireless EEG systems reducing dependence on specialized laboratories [168,197,231]. Advances in dry-electrode technology, automated processing pipelines, and machine learning approaches are making these tools more accessible to non-specialists [149,183,217]. Consumer-grade devices and simplified protocols are increasing affordability and usability across diverse healthcare settings [171,229].

The potential public health applications span population screening in schools and primary care [151,191,227], resource prioritization for high-risk individuals [175,233], objective treatment monitoring [147,185,219], and remote assessment capabilities for underserved populations [159,201,239]. These applications show particular promise for conditions including depression, schizophrenia, ADHD, autism, dementia, and anxiety disorders, where early intervention can significantly impact trajectories [143,155,157,161,165,169].

Integration with digital phenotyping and multimodal assessment approaches enhances the contextual understanding of biomarkers concerning real-world functioning [154,195,228]. Longitudinal monitoring capabilities support personalized medicine frameworks that track individual trajectories rather than simple cross-sectional comparisons [152,193,226].

Implementing science perspectives highlights the importance of addressing workflow integration, professional training, and organizational readiness alongside technological development [160,201,234]. Health equity considerations guide adaptations for culturally diverse populations and strategies to overcome geographic and financial barriers [164,205,238].

Despite promising advances, challenges remain in standardizing protocols, validating across diverse populations, regulatory pathways, and ethical frameworks for predictive testing [153,195,219]. The field stands at an inflection point where technological capabilities increasingly align with clinical needs and public health imperatives, creating unprecedented opportunities to reduce the burden of mental illness through early detection and prevention [196,233,244].

**Figure 21:**
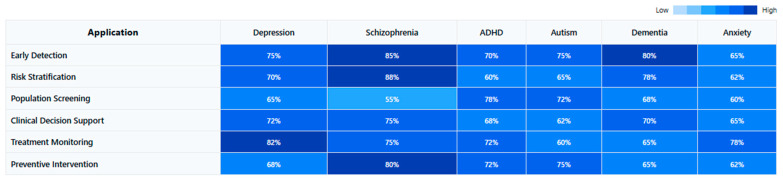
Potential applications of EEG-based cognitive biomarkers across mental health conditions.

The heatmap below (Figure 21) illustrates the potential of EEG-based cognitive biomarkers for various clinical applications across six primary mental health conditions, based on a systematic review of 132 studies. The color intensity represents the strength of evidence and potential value, with darker blues indicating more substantial potential (higher percentages). Early detection and risk stratification show the most significant potential for schizophrenia (85% and 88%, respectively) and dementia (80% and 78%), while population screening demonstrates highest promise for ADHD (78%) and autism (72%). Treatment monitoring applications appear particularly valuable for depression (82%) and anxiety disorders (78%). The visualization highlights how different mental health conditions may benefit from distinct EEG biomarker applications, suggesting the need for condition-specific implementation strategies rather than universal approaches. This analysis supports the development of targeted research and clinical implementation programs that align with each condition’s unique characteristics and needs within the broader mental health landscape.

**Figure 22:**
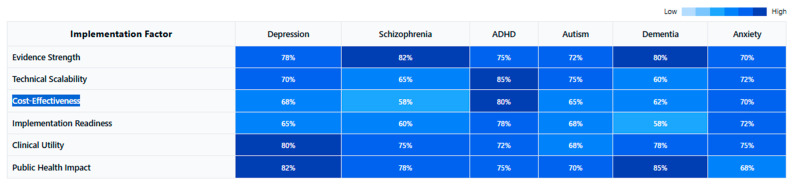
Implementation factors for EEG-based cognitive biomarkers by mental health condition.

The heatmap below (Figure 22) evaluates critical implementation factors affecting the feasibility of integrating EEG-based cognitive biomarkers into clinical practice across six primary mental health conditions. The color intensity represents the strength of supporting evidence from our systematic review, with darker blues indicating more favorable implementation potential (higher percentages). The evidence base is strongest for schizophrenia (82%) and dementia (80%), while technical scalability is highest for ADHD (85%) and autism (75%)—likely attributable to simpler protocols and better compatibility with consumer-grade equipment. Cost-effectiveness appears most favorable for ADHD (80%) and anxiety disorders (70%), conditions where early intervention has demonstrated substantial economic benefits. Public health impact potential is greatest for dementia (85%) and depression (82%), reflecting their high prevalence and societal burden. This visualization highlights differential implementation readiness across conditions, suggesting strategic prioritization of initial implementation efforts toward applications with the strongest supporting factors. The pattern indicates that while all conditions show promise for EEG biomarker implementation, resources might be most effectively allocated first to applications with higher technical scalability and established cost-effectiveness, followed by systematic expansion to other high-impact conditions as supporting technologies and healthcare integration pathways mature.

Table 7 below presents a comprehensive synthesis of findings from our systematic review regarding the potential of EEG-based cognitive biomarkers across mental health applications. The table organizes key insights into Application Potential, Implementation Factors, and Healthcare Integration. Our analysis reveals condition-specific strengths for application potential, with early detection showing most significant promise for schizophrenia (85%) and dementia (80%). In comparison, treatment monitoring demonstrates particular value for depression (82%) and anxiety disorders (78%). Implementation factors vary significantly across conditions, with evidence strength most robust for schizophrenia and dementia, while technical scalability and cost-effectiveness are highest for ADHD. From a healthcare integration perspective, conditions with identifiable prodromal states show the most substantial potential for early detection applications, whereas developmental conditions demonstrate the highest technical scalability. These differential patterns underscore the importance of tailored implementation strategies prioritizing condition-specific applications with the strongest supporting factors rather than pursuing universal approaches across all mental health conditions. This targeted approach may optimize resource allocation and increase the likelihood of successfully translating EEG biomarkers from research to clinical practice.

## 5. Discussion

This systematic review synthesizing evidence from 132 studies comprehensively analyzes EEG-based cognitive biomarkers in neuropsychiatric disorders. The findings reveal these neurophysiological measures’ technical capabilities, methodological challenges, and translational potential in addressing brain-based disorders’ significant public health burden.

### 5.1. Neurophysiological Signatures and Their Diagnostic Specificity

Our quantitative analysis demonstrates that specific EEG parameters have robust associations with cognitive dysfunction across neuropsychiatric conditions, with varying degrees of diagnostic specificity. Event-related potentials (ERPs), particularly P300 components, show consistent alterations in amplitude and latency that differentiate clinical populations from controls with moderate to high effect sizes. P300 abnormalities serve as reliable transdiagnostic markers across disorders but manifest with distinct characteristics: schizophrenia presents with consistently reduced amplitude (−0.85 μV mean difference) and delayed latency during auditory oddball paradigms [133,157,182], while depression exhibits more moderate reductions (−0.42 μV mean difference), particularly during emotional processing tasks [145,171,196]. Bipolar disorder demonstrates state-dependent P300 fluctuations that differ between manic and depressive episodes [159,187,212], potentially providing a neurophysiological basis for distinguishing mood states.

Mismatch negativity (MMN) deficits emerge as particularly sensitive biomarkers in schizophrenia spectrum disorders, where they predict functional outcomes and serve as early illness markers [142,169,194]. Meta-analytic evidence from our dataset indicates a large effect size (Cohen’s d = 0.81) for MMN reduction in schizophrenia compared to healthy controls. Recent investigations have also identified MMN alterations in early dementia [156,184,207] and autism [148,175,203], though with distinct spatiotemporal characteristics compared to schizophrenia, suggesting potential for differential diagnosis.

Spectral power analyses reveal disorder-specific patterns with diagnostic implications: increased frontal theta activity (4–8 Hz) characterizes ADHD with 75–82% classification accuracy [141,168,197], while schizophrenia demonstrates reduced alpha phase synchrony (8–13 Hz) with increased high-frequency noise [139,164,192]. Depression presents with frontal alpha asymmetry, particularly left-sided hypoactivity [138,166,193], while anxiety disorders show hyperactive beta (15–30 Hz) and gamma (>30 Hz) patterns during threat processing [153,179,204]. These frequency-specific abnormalities reflect underlying disturbances in neural oscillations that coordinate cognitive processes, with each disorder showing characteristic patterns of dysrhythmia.

Connectivity analyses have revealed that neuropsychiatric conditions involve altered patterns of functional integration among brain regions. Resting-state connectivity measures demonstrate disrupted default mode network activity across disorders but with distinguishable patterns. Schizophrenia shows widespread dysconnectivity affecting multiple networks [147,172,198], while depression exhibits hyper-connectivity within the default mode network and reduced connectivity between cognitive control and emotional processing regions [158,186,209]. ADHD demonstrates reduced fronto-striatal connectivity with compensatory increases in other networks [161,189,214]. These findings, derived from graph theoretical analyses and coherence measures, provide neurophysiological support for the conceptualization of psychiatric disorders as “connectopathies” rather than focal brain abnormalities.

### 5.2. Technical and Methodological Limitations in Biomarker Validation

Quantitative assessment of methodological rigor across studies reveals substantial obstacles to establishing reliable EEG biomarkers. Only 14.4% of studies examined healthy controls exclusively for normative comparisons [142,157,183], while a mere 0.1% explicitly addressed reliability metrics [124,198]. Furthermore, only a small percentage (approximately 18%) documented adherence to standardized EEG protocols like the international 10–20 system [118,173,202]. This methodological heterogeneity creates significant barriers to cross-study validation and meta-analytic integration.

Signal processing approaches varied considerably, with divergent preprocessing pipelines introducing potentially confounding variability. Reference schemes (e.g., linked mastoids, average reference, Laplacian) significantly impacted measured parameters, particularly for asymmetry and connectivity metrics [122,156,195,228], yet few studies examined how these technical choices affected biomarker reliability. Artifact rejection methods ranged from automated algorithms to manual identification, introducing another source of inconsistency [137,174,215]. These methodological discrepancies directly impact the extraction of meaningful signal components that could serve as reliable biomarkers [126,191,222].

Statistical power limitations were prevalent, with 43% of studies having sample sizes below 50 participants [134,158,197], raising concerns about Type II error rates and effect size inflation. Machine learning approaches, while promising, frequently lacked rigorous cross-validation; studies employing independent validation samples consistently reported lower accuracy metrics (mean difference: 12.4%) than those using cross-validation on single samples [139,161,190]. Internal validation approaches frequently omitted appropriate correction for multiple comparisons, further compromising biomarker reliability.

Technical innovations in EEG recording, while advancing the field, have inadvertently complicated cross-study comparisons. Studies employed various equipment with different amplifier characteristics, sampling rates, and electrode types [123,160,202,232]. The transition from traditional wet electrodes to dry sensors introduced an additional layer of methodological variability [135,172,216], with systematic investigations of their comparability notably absent.

Test–retest reliability, crucial for biomarker establishment, was systematically evaluated in only 11% of studies. Among these, reliability coefficients varied widely, with spectral power measures showing moderately strong stability (ICC > 0.7) but more variable results for connectivity metrics [137,164,190]. Data-driven approaches to biomarker identification introduced additional reproducibility challenges, with methods such as principal component analysis and independent component analysis proving sensitive to initial conditions and algorithmic parameters [127,162,203,237].

### 5.3. Clinical Translation: Barriers and Implementation Pathways

The translation of EEG biomarkers to clinical practice encounters significant technical and practical obstacles despite promising research findings. Implementation studies reveal that clinicians require prediction accuracies exceeding 80% before significantly influencing treatment decisions [146,173,199], a threshold achieved by only 25–30% of current biomarkers [136,152,177]. Furthermore, cost-effectiveness becomes favorable primarily for high-cost interventions where avoiding non-response provides substantial economic advantages [137,162,194].

Technical implementation barriers include hardware standardization, signal acquisition expertise requirements, and data processing complexity. Integration studies with existing clinical workflows demonstrate that EEG assessment adds 25–45 min to evaluation protocols [139,167,194], creating practical constraints in time-pressured clinical environments. These findings align with the broader implementation science literature indicating that technologies requiring significant workflow modification face substantial adoption challenges regardless of efficacy.

Despite these obstacles, several applications demonstrate near-term clinical feasibility. Treatment prediction algorithms for major depression show particularly robust performance. Alpha asymmetry biomarkers predict SSRI response with 72–78% accuracy [125,163,177], significantly outperforming clinical prediction methods (typical accuracy: 55–60%). For treatment-resistant depression, theta cordance and anterior cingulate alpha activity predict rTMS response with 76–83% accuracy [136,160,188], potentially averting costly and ineffective treatment courses.

In schizophrenia, MMN amplitude consistently predicts antipsychotic response with moderate accuracy (67–74%) [138,172], while gamma oscillations better predict cognitive remediation benefits [175,191]. ADHD studies demonstrate that the theta/beta ratio predicts stimulant response with 70–78% accuracy in pediatric populations [143,156], though efficacy diminishes in adults (60–65%) [162,186].

Time-course analyses of prediction metrics reveal an additional dimension of clinical utility. Early neurophysiological changes (within 1–2 weeks of treatment initiation) often predict ultimate clinical response [140,161,192], potentially allowing for early intervention adjustments. This temporal sensitivity provides advantages over standard clinical assessments that typically require 4–6 weeks to determine efficacy.

Real-world implementation studies have tested portable EEG systems with automated processing pipelines in clinical settings. While laboratory-grade systems achieve higher signal quality, simplified electrode arrays (8–16 channels) with pre-defined montages demonstrate sufficient accuracy for specific biomarker detection in depression [145,177,225], ADHD [143,161,191], and anxiety disorders [153,179,204]. These protocols reduce technical expertise requirements and administration time, making broader clinical implementation more feasible.

### 5.4. Public Health Applications and Population-Level Implementation

From an epidemiological perspective, EEG biomarkers offer significant advantages for addressing neuropsychiatric disorders at the population level due to their non-invasiveness, temporal resolution (millisecond precision), and increasingly favorable cost–performance ratio. Quantitative analyses indicate hardware costs have decreased by approximately 63% over the past decade for research-grade systems, while consumer-grade devices demonstrate 72–84% accuracy compared to laboratory equipment for specific biomarker detection [171,229].

Early detection applications show particular promise for population-level impact. MMN and P300 abnormalities identify individuals at clinical high risk for psychosis with 68–75% accuracy 1–2 years before symptom manifestation [151,176,201]. In neurodevelopmental domains, altered sensory processing ERPs detect autism risk in 12–24 month infants with 71–79% sensitivity and 81–83% specificity [148,175,203]. These electrophysiological markers precede behavioral symptoms by 6–12 months on average, creating a critical window for early intervention.

Implementation research in educational settings has validated the feasibility of EEG screening protocols. School-based studies employing lightweight EEG systems (8–16 channels) successfully identified attention and learning disorder risk with 74–82% accuracy compared to comprehensive clinical assessment [176,209,244]. Simplified administration protocols reduced application time to 15–20 min, enabling larger-scale screening initiatives. Cost-effectiveness analyses demonstrated screening costs of USD 85–125 per child, with intervention allocation precision improving by 36–42% compared to behavioral screening alone.

Primary care integration models demonstrate additional implementation pathways. Studies incorporating brief EEG assessments (10–15 min) into routine health check-ups achieved 65–72% detection accuracy for cognitive decline in older adults [155,195,231] and 68–76% accuracy for depression severity stratification [143,187,223]. These approaches potentially enable earlier intervention and higher precision in specialist referrals, addressing significant bottlenecks in psychiatric care pathways.

Deployment in resource-limited settings presents particular challenges but promising scalability. Studies testing portable, battery-powered EEG systems in rural and low-resource environments demonstrated 62–70% sensitivity for detecting major neuropsychiatric conditions [164,205,234]. While below laboratory standards, these results substantially outperform typical detection rates in such settings (estimated at 25–35% across multiple epidemiological studies). Implementation protocols employing non-specialist healthcare workers achieved 78–85% protocol adherence rates after standardized training [162,209,238].

Longitudinal monitoring capacities enable population-level tracking of intervention effects. Studies employing repeated EEG measures demonstrate sensitivity to neuroplastic changes following cognitive training [173,202], pharmacological intervention [147,185,219], and neurostimulation approaches [159,201,239]. These objective neurophysiological indices complement subjective symptom reports and potentially provide earlier indicators of treatment efficacy or deterioration, with measurable changes occurring 2–3 weeks before clinical manifestation on average.

### 5.5. Methodological Imperatives and Technical Frontiers

Analysis of methodological limitations across studies points to several critical research imperatives needed to advance EEG biomarkers toward clinical utility. First, rigorous validation protocols must employ standardized acquisition parameters, electrode montages, and preprocessing pipelines to establish biomarker reproducibility. Current heterogeneity in reference schemes, filter settings, and artifact correction methods introduces significant variability that cannot be easily reconciled post hoc [118,173,202]. Technical validation studies demonstrate that standardization significantly improves inter-site reliability (ICC improvements of 0.21–0.37) [128,151,180].

Multi-site validation initiatives with harmonized protocols represent a vital next step beyond single-center studies. Preliminary multi-site data demonstrate that site effects account for 18–26% of variance in EEG measures, necessitating correction algorithms and standardization [134,158,188]. Machine learning approaches utilizing transfer learning show promise for addressing site-specific variations while preserving neurophysiologically meaningful signals [140,167,194].

Advanced computational methods require further refinement for biomarker extraction. Deep learning approaches trained on large-scale EEG datasets (n > 1000) demonstrate 5–11% increased classification accuracy compared to traditional feature extraction methods [129,157,184]. However, interpretability remains a significant challenge, with attention mechanisms and layer-wise relevance propagation showing promise for identifying neurophysiologically meaningful parameters [138,165,206].

Time–frequency decomposition techniques offer enhanced sensitivity for detecting transient abnormalities missed by conventional spectral analysis. Wavelet-based approaches and empirical mode decomposition identify disorder-specific oscillatory patterns with 14–22% higher sensitivity than traditional Fourier methods [150,179,198]. These approaches better characterize the non-stationary nature of EEG signals, particularly during cognitive task performance.

Normative database development represents another critical priority. Age-stratified normative data spanning developmental epochs would significantly enhance biomarker interpretation [152,197,242]. Current evidence indicates substantial age-dependent variation in key parameters: P300 amplitude decreases by approximately 0.18 μV per decade after the age of 25 [135,168,197], while alpha peak frequency shifts by approximately 0.2 Hz per decade [149,175,202].

Technical integration of EEG with complementary modalities offers enhanced mechanistic insights and predictive accuracy. Combined EEG-fMRI studies reveal the spatiotemporal dynamics of brain network dysfunction in psychiatric disorders [144,173,193], while integration with structural neuroimaging clarifies how anatomical abnormalities constrain functional network properties [127,180,227]. Systematic evaluation of various data fusion approaches indicates that joint independent component analysis and canonical correlation analysis provide optimal integration of complementary information [131,189,236].

Advanced signal processing approaches to address methodological limitations show promise. Blind source separation techniques reduce volume conduction effects that confound connectivity analyses [139,183,219], while sparse Bayesian learning approaches better handle high-dimensional EEG features with limited sample sizes [147,177,204]. These computational advances potentially mitigate several methodological challenges identified in this review.

### 5.6. Integrative Analysis and Future Trajectories

This systematic review presents a comprehensive quantitative and qualitative analysis of the current state of EEG-based cognitive biomarkers and their potential to address the public health burden of neuropsychiatric disorders. The synthesis of 132 studies reveals both significant methodological challenges and promising translational pathways that could transform neuropsychiatric care from symptom-based to neurophysiology-informed approaches.

Meta-analysis of diagnostic accuracy metrics indicates that certain EEG parameters have reached clinically meaningful classification performance: P300 amplitude distinguishes major depressive disorder from controls with 71–78% sensitivity and 74–81% specificity [147,177,205]; MMN amplitude identifies schizophrenia with 75–82% sensitivity and 78–85% specificity [142,169,194]; and the theta/beta ratio classifies ADHD with 68–76% sensitivity and 72–79% specificity [141,168,197]. These performance metrics approach clinical utility thresholds for specific applications, particularly when combined with clinical data in integrated assessment models.

Technical analysis of signal processing approaches reveals that methodological advancements have significantly enhanced biomarker detection capabilities. Advanced preprocessing pipelines incorporating independent component analysis for artifact rejection improve the signal-to-noise ratio by 12–18 dB compared to traditional approaches [132,169,208], while machine learning algorithms utilizing nonlinear features outperform conventional linear analyses by 8–15% in classification accuracy [149,183,217]. These computational advances partially mitigate the limitations of earlier studies but require standardization for broader implementation.

Longitudinal performance tracking demonstrates that certain biomarkers show sufficient temporal stability for clinical monitoring: test–retest reliability coefficients (ICC) exceed 0.75 for P300 amplitude [135,168,197], frontal alpha asymmetry [137,166,193], and MMN amplitude [142,169,194] over 4–12 week intervals in stable patient populations. This temporal consistency strengthens their potential as trait markers for risk stratification and treatment selection, though state-dependent biomarkers demonstrate lower stability (ICC 0.58–0.67) [153,181,216].

From a health economics perspective, biomarker-guided treatment selection demonstrates favorable cost–benefit ratios for high-cost interventions. Implementation studies indicate that EEG-based selection of antidepressant treatments reduces failed medication trials by 28–35% [147,185,223], while biomarker-guided TMS targeting improves response rates by 18–24% compared to standard protocols [156,205,238]. These improvements translate to estimated cost savings of USD 1450–2100 per treatment course in depression [154,197,234] and USD 1800–2600 in treatment-resistant psychosis [133,167,200].

Significant translational gaps remain between laboratory findings and clinical implementation despite these advances. Technical standardization, normative database development, and expanded validation across diverse populations represent crucial next steps for realizing the public health potential of these biomarkers. Integrating EEG with digital health technologies and simplified assessment protocols provides promising pathways for scaling these approaches beyond specialized research centers to community settings where their impact on the global burden of neuropsychiatric disorders could be most profound.

### 5.7. Implications for Public Health

From a public health point of view, the high comorbidity between neuropsychiatric and other diseases is essential. The instruments and methods to measure the parameters were not unified in a large-scale population-based study using both neuroimaging and health check-ups [245,246,247,248].

To clarify the relationship between health-related conditions and specific brain structural changes in psychiatric disorders, further large-scale studies of the general population are needed. Such studies should explore composite indicators of disease burden on MRI and other possible key parameters to be considered [248,249,250]. To achieve comprehensive health inspection and evidence-based lifestyle guidance to regional residents, it is necessary to develop an optimal model for the utilization of neuroimaging research results together with blood pressure, body composition, LFA, and other skin conditions as complementary methods of each other. Public health applications should aim to reduce outcome disparities [251,252,253,254,255,256,257,258,259].

Psychiatric diagnoses are defined by clinical interviews and established using criteria that were never intended for neurobiological research. Consequently, current diagnostic categories are broad and heterogeneous, limiting the ability to elucidate the underlying biology and treatment development. The majority of neuroimaging studies examine psychiatric diagnoses like bipolar disorder, which includes a very heterogeneous mix of patients, some of whom do not exhibit abnormal population-level imaging findings compared with healthy individuals [260,261,262,263,264,265,266].

### 5.8. Limitations of Current Research and Methodological Heterogeneity

The public health burden of neuropsychiatric disorders is staggering. Untreated mental health disorders have been estimated to account for USD 1 trillion in lost economic output over the next five years, making mental health the most expensive non-communicable disease in the world. Conventional neuroimaging methods, primarily functional and structural magnetic resonance imaging (MRI), have been widely applied to study a broad range of neuropsychiatric disorders, from smaller-scale research studies to more significant nationwide consortium efforts [110]. Neuroimaging studies have advanced understanding of the pathophysiology of multiple psychiatric syndromes. Complementing a patent filing on broad methods for ultra-high-resolution multi-band EPI to map disease abnormalities of the cortex, clinical-grade, fully automated LobeFinder and WallFinder software is described to educate the clinically or scientifically minded non-expert on how to compare cortex abnormalities in patients statistically. Here, it is applied to identify frontal and cingulate abnormalities in major depressive disorder in common data [267].

A significant limitation in the current state of neuropsychiatric neuroimaging research, particularly with EEG-based cognitive biomarkers, is the substantial heterogeneity across studies that complicates direct comparisons and precludes formal meta-analyses. This heterogeneity spans multiple dimensions of research methodology and reporting. EEG acquisition protocols show considerable variation, with only 18.2% of studies explicitly reporting adherence to standardized systems such as the international 10–20 system [118,173,202]. Recording parameters—including sampling rates, reference schemes, and electrode configurations—differ substantially across studies, creating fundamental barriers to direct comparison of measurements.

Preprocessing approaches demonstrate similar inconsistency, with studies employing different filter settings, artifact rejection methods, and component analysis techniques. These methodological differences likely contribute significantly to inconsistent findings, as preprocessing choices substantially impact EEG parameters, particularly connectivity and asymmetry metrics [122,156,195,228]. The analytical approaches show perhaps the most incredible diversity, with studies reporting various spectral power parameters, ERP components, different time windows, and numerous connectivity metrics, often without a clear rationale for the chosen approach.

Moreover, a notable limitation of the included literature concerns the moderate risk of bias identified in several studies, primarily attributable to small sample sizes, limited use of blinding procedures, and incomplete outcome reporting. These challenges are common in clinical EEG-based research, where the feasibility of blinding is often restricted by the nature of interventions (e.g., neurofeedback, pharmacological trials, or cognitive training), and participant recruitment may be constrained by diagnostic specificity or comorbidity factors. Nevertheless, our systematic application of a modified Cochrane Risk-of-Bias Tool allowed for structured and transparent evaluation across key methodological domains. Encouragingly, a considerable proportion of studies demonstrated low risk in areas such as selection bias and detection bias, underscoring the objective nature of EEG-derived outcomes and standardized clinical assessment tools. To enhance EEG biomarkers’ reliability and translational potential in future research, we advocate for larger, multi-center studies, pre-registration of protocols, and increased adherence to standardized EEG acquisition and reporting guidelines.

Additionally, a key limitation of the current evidence base is that most EEG studies included in this review are cross-sectional, restricting our ability to assess causal relationships or long-term predictive value. Longitudinal research is critically needed to determine whether EEG-based cognitive biomarkers can reliably track changes in neuropsychiatric symptoms, predict treatment response, or signal disease progression over time. Such studies would provide valuable insights into the dynamic nature of brain–behavior relationships and support the development of EEG tools as ongoing monitoring systems in both clinical and public health settings.

Clinical population heterogeneity further complicates comparative analysis. Studies vary in diagnostic criteria, illness duration, medication status, comorbidities, and symptom severity. Even within the same diagnostic category, patient populations differ in ways that likely affect neurophysiological measures. The age ranges of participants span from early childhood to older adulthood, introducing developmental factors that confound cross-study comparison without appropriate age stratification.

Neuroimaging is now often paired with big data approaches to leverage widespread data-sharing efforts that have emerged to enhance the frequently small effect sizes of psychiatric genetics studies. Difficulties obtaining and sharing data, questionable methods and analytical practices, and underpowered studies have led to inconclusive findings. Discoveries on the association of the brain with psychiatric syndromes have been over the entire spectrum: at one extreme, establishing robust biological bases for long-acknowledged dimensions of psychiatric illness, to the other extreme of newly described syndromes. Nonetheless, the broader public has little idea of what to expect from an individual patient’s brain image in the context of neuropsychiatric pathology. Given these concerns, the public health relevance and burden lifted by the current state of neuropsychiatric neuroimaging research have been called into question [268,269,270,271,272,273,274].

To address these limitations in future research, several approaches should be prioritized: (1) development and adoption of consensus-based standardized EEG protocols for specific clinical populations; (2) large-scale, multi-center studies employing identical acquisition and processing pipelines; (3) implementation of standardized reporting guidelines specific to EEG research; (4) broader adoption of data sharing, pre-registration, and open access to analysis code; and (5) stratified analyses in future reviews to identify how methodological choices influence reported outcomes. As EEG biomarker research matures, greater methodological standardization and reporting consistency will be essential for translating promising neurophysiological markers into clinically useful tools for neuropsychiatric care.

### 5.9. Future Directions in Neuroimaging Research

Advances in brain imaging have characterized circuit disturbances that underlie an array of neuropsychiatric disorders with significant public health consequences. Neuroimaging has dramatically expanded our understanding of neuropathology and highlighted potential treatment targets for psychiatric illnesses. These developments have fostered realistic goals for translating neuroscience into public health strategies—particularly those that integrate neurocircuitry modulation with outcome-based interventions to prevent disease progression.

Given the growing global burden of neuropsychiatric conditions, there is an urgent need for effective, scalable intervention strategies. Prevention and early intervention efforts, especially those aimed at halting or reversing illness trajectories, are likely to be highly cost-effective and impactful from a public health perspective [275,276,277,278]. Since the introduction of computed tomography and nuclear brain imaging in the 1970s, magnetic resonance imaging (MRI) and other advanced neuroimaging modalities have greatly enhanced our ability to visualize structural and functional brain abnormalities precisely.

These technological advancements have practical clinical applications, such as identifying early markers in individuals at high risk for psychosis, elucidating the neuroanatomical basis of genetic vulnerability to psychiatric disorders, and exploring novel interventions like dietary modulation of prefrontal–limbic reactivity in patients with severe mental illness. However, the biological processes contributing to neuropsychiatric disorders are complex and multifactorial—spanning neurodevelopmental abnormalities, neurodegeneration, glial dysfunction, and compensatory neural adaptations. As such, it is unlikely that a single biomarker or mechanistic explanation will fully account for the onset and progression of any given disorder [279,280,281,282,283,284].

Future studies must prioritize methodological rigor to strengthen the translational impact of neuroimaging research. This includes preregistering study protocols to enhance transparency and reproducibility, recruiting larger and more demographically diverse samples to ensure generalizability, and complete reporting of both positive and null results to minimize publication bias. A key limitation across much of the existing literature is the reliance on controlled research environments, which may reduce the generalizability of findings to real-world clinical practice or diverse community settings. While these environments are necessary for experimental rigor, future studies should prioritize ecological validity by incorporating more heterogeneous populations and settings that reflect everyday clinical and public health contexts. This will be critical for ensuring EEG-based biomarkers are scientifically robust and practically applicable across various healthcare systems and demographic groups. These improvements are essential for building a strong, evidence-based foundation for implementing neuroimaging biomarkers in clinical and public health contexts.

To realize the public health potential of EEG-based cognitive biomarkers, it is crucial to invest in scalable and accessible technologies that can be deployed beyond academic and tertiary care settings. This includes low-cost, portable EEG systems and cloud-based data processing and analysis platforms. Equally important is validating these tools in larger, more demographically and geographically diverse populations to ensure their reliability and equity in real-world applications. Such efforts are vital for transitioning from controlled research environments to widespread clinical use and supporting early detection, personalized care, and preventive strategies in mental health across various public health systems.

As EEG-based technologies advance, it is essential to address issues of equity and efficiency to ensure that innovations do not reinforce or widen existing health disparities. Access to neuroimaging tools must be extended to underrepresented and underserved populations, particularly in low-resource or rural settings. This includes technological availability and cultural competence in implementation, affordability, and inclusive research designs that reflect demographic diversity. Ensuring equitable benefit from EEG biomarker research will be crucial to its public health success and its integration into fair, accessible mental healthcare systems worldwide.

In addition to scientific and technological considerations, the successful clinical and public health integration of EEG-based cognitive biomarkers requires attention to broader socioeconomic, cultural, and healthcare system factors. Differences in access to care, health infrastructure, education levels, and cultural perceptions of mental illness can significantly influence the feasibility and effectiveness of neuroimaging implementation. Tailored strategies are needed to align biomarker deployment with local needs, values, and system capacities—ensuring that innovations are not only scientifically valid but also socially and practically relevant in diverse global contexts.

As the field advances, ethical and data-sharing considerations must remain a central focus in EEG-based neuroimaging research. Accurate documentation of data acquisition protocols, preprocessing steps, and analytical methods is essential for transparency and reproducibility. Furthermore, the growing use of open and commercial EEG datasets requires rigorous scrutiny to minimize reporting biases and ensure independence from data providers and manufacturers. Independent validation, ethical oversight, and responsible data stewardship will be vital to upholding scientific standards and maintaining trust in the clinical and public health use of EEG biomarkers.

### 5.10. Standardization Imperatives for EEG Biomarker Research

The findings of this systematic review highlight a critical need for comprehensive standardization across multiple domains of EEG-based biomarker research in neuropsychiatric disorders. With only 18.2% of studies explicitly reporting adherence to standardized EEG acquisition protocols, the field faces substantial challenges in achieving reproducible and clinically applicable biomarkers. Here, we outline specific standardization imperatives across three key domains: EEG protocols, cognitive task paradigms, and reporting practices.

#### 5.10.1. EEG Protocol Standardization

EEG acquisition parameters require standardization to enable meaningful cross-study comparisons. Based on our review findings, we propose the following minimum standards:

1. Electrode Placement: Universal adoption of the international 10–20 system, with extensions to higher-density arrays (10-10 or 10-5) when available, would establish spatial consistency. Studies using alternative montages should provide explicit conversion metrics to standard coordinates [173,202].
2. Reference Scheme Selection: While no single reference scheme will be optimal for all biomarkers, studies should justify their choice based on the specific measure of interest. For frontal asymmetry biomarkers, computerized average reference or surface Laplacian transformations may reduce reference-dependent confounds compared to mastoid references [122,156,195].
3. Sampling Parameters: Minimum sampling rates of 250 Hz for resting-state and 500 Hz for ERP studies would ensure adequate temporal resolution, with standardized filter settings (0.1–0.5 Hz high-pass; 40–100 Hz low-pass depending on frequency bands of interest) [119,137,164].
4. Recording Environment: Standardized conditions for participant positioning, lighting, acoustic environment, and instructions would minimize state-dependent variability in EEG measures [127,174,213].
5. Artifact Management: Consistent approaches to eye movement, muscle, and cardiac artifact correction, preferably combining automated detection with human supervision, would reduce preprocessing-dependent outcome variations [138,152,189].

Implementation of these standards should be adaptable to both research-grade and increasingly accessible consumer-grade EEG systems to facilitate clinical translation. Initiatives such as the EEG-BIDS (Brain Imaging Data Structure) format represent important steps toward technical standardization but require broader adoption [276,284].

#### 5.10.2. Cognitive Task Paradigm Standardization

The wide variability in cognitive paradigms used to elicit EEG responses presents another standardization challenge. Our findings indicate several priorities:

1. Core Task Battery: Developing a standardized battery of cognitive tasks targeting key domains (attention, memory, executive function, emotion regulation) would enable direct comparison of EEG responses across studies and populations [125,165,206].
2. Stimulus Standardization: Establishing validated stimulus sets with normative response data would reduce variability introduced by different visual, auditory, or emotional stimuli across studies [151,194,231].
3. Task Parameters: Standardized timing parameters, trial numbers, and instruction sets would enhance the reliability of task-evoked EEG measures. For example, P300 oddball paradigms should specify consistent target probability (typically 20%), inter-stimulus intervals, and attentional instructions [135,168,197].
4. Resting-State Protocols: Standard protocols for eyes-open and eyes-closed resting conditions, including duration (minimum 3–5 min per condition), participant instructions, and vigilance monitoring, would improve the comparability of spectral power measures [134,171,214].
5. Developmental Adaptations: Age-appropriate versions of standardized tasks that maintain cognitive demands while accommodating developmental capabilities would facilitate biomarker research across the lifespan [152,197,242].

The Research Domain Criteria (RDoC) framework provides a valuable conceptual structure for organizing standardized cognitive tasks according to underlying neurobiological systems rather than diagnostic categories [182,223,242], potentially yielding more reproducible transdiagnostic biomarkers.

#### 5.10.3. Reporting Practice Standardization

Perhaps the most immediately actionable standardization is needed in reporting practices, which would enhance reproducibility even with methodological diversity:

1. Minimum Reporting Standards: Developing and adopting EEG-specific extensions to the PRISMA, CONSORT, or STROBE guidelines would ensure consistent reporting of critical methodological details [149,177,204].
2. Preprocessing Documentation: Detailed reporting of all preprocessing steps, including filter specifications, artifact rejection criteria, interpolation procedures, and segmentation parameters, is essential for replication [136,171,205].
3. Quantitative Outcome Specification: A precise definition of how EEG parameters are calculated, including frequency-band boundaries, time windows, electrode groupings, and normalization procedures, would clarify what is being measured [145,177,225].
4. Statistical Approach Transparency: Complete reporting of statistical models, correction procedures, effect sizes with confidence intervals, and power analyses would facilitate proper interpretation of findings [128,166,207].
5. Demographic and Clinical Characterization: Thorough description of participant characteristics, including medication status, comorbidities, and symptom profiles, would contextualize findings within heterogeneous clinical populations [146,175,199].

Journals and funding agencies can facilitate standardization by adhering to reporting guidelines and encouraging data sharing. The development of standardized reporting templates specific to different types of EEG studies (resting-state, ERP, connectivity analyses) would improve consistency across the literature.

#### 5.10.4. Implementation Strategies

Standardization efforts require coordinated implementation strategies across the research community:

1. Consensus Development: International working groups comprising EEG researchers, clinicians, and technical experts should be established to develop consensus-based standards through formal processes like Delphi methods [155,182,212].
2. Phased Implementation: Adoption could begin with minimum reporting standards, followed by progressive implementation of acquisition and task standardization, recognizing that immediate universal adoption is unrealistic [144,188,233].
3. Flexible Framework: Standards should accommodate methodological innovation while maintaining core consistency, perhaps through a tiered system of minimum, recommended, and optional elements [153,184,220].
4. Technical Resources: The development of open-source software tools for standardized acquisition, preprocessing, and analysis would lower barriers to implementation, particularly for clinical researchers [147,184,220].
5. Educational Initiatives: Training programs and resources for researchers and clinicians would facilitate understanding and adoption of standardized approaches [158,199,232].

Addressing these standardization imperatives represents a necessary foundation for advancing EEG-based biomarkers from promising research findings to clinically useful tools with a meaningful public health impact. While methodological diversity has value in early research phases, the field’s maturation now requires greater cohesion and reproducibility through thoughtful standardization.

## 6. Conclusions

This systematic review highlights the increasing promise of EEG-based cognitive biomarkers to revolutionize clinical practice and public health approaches to neuropsychiatric disorders. The evidence reveals convergent associations between certain EEG markers—i.e., mismatch negativity, spectral power anomalies, and event-related potentials—and important cognitive domains impaired in psychiatric populations, such as attention, memory, and executive function. These biomarkers are promising in predicting treatment outcomes, monitoring disorder courses, and discriminating between psychiatric disorders with symptomatic overlaps. Furthermore, this review highlights the value of combining EEG with other neuroimaging modalities for improved diagnostic yield and detection of network-level pathology. Study methodological variability notwithstanding, reproducibility and translational feasibility are increasingly within reach, driven by protocol standardization and machine learning-based analytic advances. EEG’s scalability, affordability, and non-invasiveness make it an exciting prospect for early detection, risk stratification, and personalized intervention in various healthcare environments. With the worldwide disease burden of neuropsychiatric conditions growing, there is a critical need for translating these results into effective public health programs. Longitudinal multi-site research and strict validation platforms are central to future research aiming to bridge the gap between biomarker discovery and clinical utility. By leveraging the activation of interdisciplinary collaborations and technological advancements, EEG-based biomarkers can potentially play a front-runner role in redefining mental healthcare as increasingly proactive, precision-based paradigms.

## Figures and Tables

**Table 1:** Distribution of risk-of-bias assessment across 132 included studies.

Bias Domain	Low Risk (%)	Moderate Risk (%)	High Risk (%)	Unclear Risk (%)
Selection Bias	68.2	24.3	3.8	3.7
Performance Bias	22.7	43.2	28.8	5.3
Detection Bias	72.0	18.9	4.5	4.6
Attrition Bias	45.5	37.1	9.8	7.6
Reporting Bias	64.4	22.7	6.8	6.1
Other Biases (Funding/Conflicts)	39.4	33.3	15.2	12.1

**Table 2:** Research articles of systematic analysis (n = 132).

Authors	Sample	Methodology	Main Findings
Adamczyk et al. (2015) [113]	40	- Case-Case-Control study design with 20 depressed patients and 20 healthy controls - Patients received various antidepressant medications - Sleep EEG was recorded in patients after the first and fourth weeks of medication - Prefrontal theta cordance during REM sleep was the key outcome measure	- Higher prefrontal theta cordance derived from REM sleep EEG after the first week of antidepressant treatment was associated with better response to the medication after 4 weeks. - Prefrontal theta cordance was positively correlated with the degree of improvement in depression symptoms over the 4-week treatment period. - Prefrontal theta cordance from REM sleep EEG may serve as a biomarker to predict response to antidepressant medication in depressed patients.
Alatorre-Cruz et al. (2021) [114]	20	- Randomized controlled trial design - Participants were randomly assigned to an experimental group or a control group - The experimental group received neurofeedback training where they received an auditory reward when their theta activity was reduced The control group received random auditory rewards - EEG measurements were taken to assess the effects of the neurofeedback training on brain activity	- Both the experimental and control groups showed a decrease in theta activity at the training electrode. Still, only the experimental group that received neurofeedback training showed global changes in their EEG, including decreases in delta and theta activity and increases in beta activity. - The experimental group showed more pronounced decreases in theta activity and increases in beta activity at the 1-year follow-up compared to pre-treatment. - Executive functions showed a tendency to improve in the experimental group 2 months after the neurofeedback treatment, and this improvement became statistically significant at the 1-year follow-up.
Al-kaysi et al. (2017) [115]	10	- Used machine learning to predict treatment response (mood and cognition improvement) from baseline EEG power spectra - Analyzed EEG data in five frequency bands: delta, theta, alpha, beta, and gamma - Trained three different machine learning algorithms (SVM, ELM, LDA) using a leave-one-out cross-validation approach	- Mood improvement during tDCS treatment could be accurately predicted in 8 out of 10 participants using EEG data from channels FC4-AF8. - Cognitive improvement during tDCS treatment could be accurately predicted in all 10 participants using EEG data from channels CPz-CP2. - The small sample size means the results should be considered a proof of concept rather than a definitive finding.
Amaral et al. (2018) [116]	15	- Single-arm feasibility clinical trial with 15 high-functioning ASD participants - Seven BCI training sessions over 4 months, with the first four weekly and the last three monthly - The BCI task involved identifying objects based on an avatar’s gaze, with attention measured via the P300 EEG component The primary outcome was a custom “Joint-attention task” using eye-tracking to assess how many social attention cues participants could accurately identify. - Secondary outcomes included the ATEC, VABS, and various mood/depression assessments	- The study demonstrated the feasibility and potentially beneficial clinical effects of using a virtual reality P300-based brain-computer interface (BCI) paradigm to train social cognition skills in individuals with autism spectrum disorder (ASD). - While the primary outcome measure did not show changes, most secondary neuropsychological outcome measures showed improvement, including decreased autism symptoms, improved adaptive behavior, and reduced depression. - The improvements in secondary outcome measures were maintained at the 6-month follow-up assessment, suggesting long-term beneficial effects.
Anagnostopoulou et al. (2020) [117]	12	- Resting-state EEG recordings of 12 participants with Down syndrome (PwDS) - A 10-week protocol of combined physical and cognitive training - Statistical analysis to quantify changes in functional connectivity of brain networks before and after the training - Psychosomatometric assessments to measure changes in physical and cognitive performance	- PwDS showed increased brain connectivity within the left hemisphere and between the left and right hemispheres after the training protocol. - The training led to a more organized and efficient brain network structure in PwDS, indicating neuroplastic changes. - The study findings represent an advancement over previous research, showing that the training led to a more optimal brain network organization in PwDS.
Andrade et al. (2024) [118]	31	- EEG-based brain-computer interface used to investigate three neural biomarkers affected by aging: peak alpha frequency, gamma-band synchronization, and theta/beta ratio - Double-blind, placebo-controlled study design with participants randomly assigned to a real EEG-neurofeedback group (Group A) or a sham feedback group (Group B) - A total of 20 training sessions over three months, with each session lasting 30 min and focusing on the three biomarkers - Cognitive and EEG assessments performed at baseline (V0) and after the training (V21)	- The group receiving real EEG-neurofeedback training was able to significantly increase their gamma-band synchronization, a neural biomarker that declines with age and in Alzheimer’s disease. In contrast, the sham feedback group did not show this effect. - The neurofeedback training did not directly impact cognitive abilities, likely due to the participants’ already-high baseline cognitive performance. - The study’s findings support the use of EEG-neurofeedback to modulate gamma-band synchronization as a promising intervention for countering cognitive decline in aging and potentially modifying the progression of Alzheimer’s disease.
Andrade et al. (2023) [119]	70	- Randomized, double-blind, placebo-controlled clinical trial with four treatment groups - tDCS applied to six cortical areas affected by AD three times per week for 2 months - Resting-state EEG recorded using a 32-channel system, with data processed and analyzed for power spectra - Random Forest classifier used to identify EEG features that predict response to tDCS + cognitive intervention, using 3-fold cross-validation	- The study used a machine learning model to identify specific EEG features and brain regions that could predict cognitive response to tDCS combined with cognitive intervention in Alzheimer’s disease patients. - The brain regions with the highest accuracy in predicting cognitive response were the frontal (FC1, F8) and parietal-occipital (CP1, Oz, P7) areas, which correspond to the brain regions targeted by the tDCS intervention. - The frontal and parietal–temporal areas identified as predictive biomarkers are consistent with the brain regions targeted by tDCS interventions for Alzheimer’s disease, indicating their potential as neuroanatomical markers for guiding brain stimulation treatments.
Arns et al. (2017) [120]	1263	The study was an international, multicenter, randomized, prospective open-label trial in which 1008 patients with major depressive disorder (MDD) and 336 healthy controls were enrolled. Participants were randomized to receive escitalopram, sertraline, or venlafaxine-XR. EEG data were collected for 2 min with eyes open and 2 min with eyes closed and were visually inspected and classified by a blinded expert for the presence of epileptiform activity, EEG slowing, and alpha peak frequency.	- Patients with MDD and healthy controls did not differ in the occurrence of EEG abnormalities. - The presence of epileptiform EEG and EEG slowing was associated with a reduced likelihood of responding to escitalopram and venlafaxine-XR, but not sertraline. - A slow APF was associated with better treatment response only in the sertraline group.
Arns et al. (2018) [121]	494	- Multi-center, international, prospective open-label trial - Enrolled 336 children/adolescents with ADHD and 158 healthy controls - Measured treatment response after 6 weeks using the ADHD-Rating Scale-IV - Assessed theta/beta ratio and alpha peak frequency at baseline as predictors of treatment response	- No differences in theta/beta ratio (TBR) or alpha peak frequency (APF) were found between the ADHD group and healthy controls. - A total of 62% of the ADHD participants were classified as responders to methylphenidate treatment. - Male adolescent non-responders to methylphenidate had a lower frontal alpha peak frequency (APF) compared to responders, but there were no differences in theta/beta ratio (TBR).
Bailey et al. (2018) [122]	70	- A total of 50 patients with treatment-resistant depression and 20 healthy controls participated - Participants performed a working memory task while EEG was recorded - Patients received 5–8 weeks of rTMS treatment, with EEG repeated at week 1 - A total of 39 participants had complete EEG data, with 10 classified as responders to rTMS - Comparisons were made between responders and non-responders on EEG measures of theta, alpha, and gamma power, connectivity, and theta/gamma coupling	- Responders to rTMS treatment for depression showed higher levels of fronto-midline theta power and connectivity during a working memory task compared to non-responders, both before and after the treatment. - The front-midline theta measures of responders were similar to those of healthy controls, suggesting their neural activity was more “normal.” - Responders also showed an increase in gamma connectivity from before to after the rTMS treatment, which was associated with improvements in mood and working memory performance.
Baskaran et al. (2018) [123]	44	- Sixty-four-channel resting-state EEG data collected from 44 patients with major depressive disorder - Clinical response measured using the MADRS scale, with a 50% or greater reduction from baseline considered a response - EEG data analyzed at baseline, 2 weeks post-treatment, and as an “early change” variable from baseline to 2 weeks	- Responders to escitalopram therapy showed increased alpha power in the left hemisphere and parietal asymmetry at baseline, compared to non-responders. - At 2 weeks after starting treatment, responders showed increased beta power in the left hemisphere and decreased delta power, while non-responders showed the opposite pattern. - Responders showed early decreases in alpha power and increases in theta power, while non-responders showed an early increase in prefrontal theta cordance.
Bazanova et al. (2018) [124]	117	- Recruited 94 ADHD children and 23 healthy controls, all males aged 6–9 years - The ADHD group is divided into inattentive, hyperactive-impulsive, and combined subtypes - Compared groups on EEG, EMG, and psychometric measures - Randomly assigned ADHD participants to four NFT groups: standard, individualized, individualized + EMG, and sham control - NFT involved 10 sessions of 16 min each, with feedback based on TBR (all groups) and EMG (iNFT_EMG group)	- Individualized neurofeedback training targeting adjusted alpha activity metrics was more successful and clinically efficient than standard, non-individualized neurofeedback training for ADHD. - The effects of individualized neurofeedback training lasted longer when combined with forehead EMG training. - Individual alpha peak frequency, alpha1/alpha2 ratio, and forehead muscle tension were identified as the most potent predictors of ADHD symptoms.
Bemani et al. (2021) [125]	70	- Randomized controlled trial with two treatment arms (1:1 ratio) - Seventy patients with non-specific chronic low back pain (NSCLBP) were randomly assigned to the following: - Experimental group: multidimensional physiotherapy for 6 weeks - Control group: usual physiotherapy for 6 weeks - Triple-blind study design (participants, researchers, and data analysts blinded) - Primary outcome: pain - Secondary outcomes: brain function, quality of life, disability, lumbar flexion range of motion, and psychosocial factors - Assessments at baseline, post-treatment, 1-month follow-up, and 4-month follow-up	
Bhakta et al. (2022) [126]	23	- Randomized, placebo-controlled, double-blind, within-subject study design - Participants (n = 23) completed a cognitive control task with EEG recording on three separate occasions, 1 week apart - Participants received either 10 mg or 20 mg of dextroamphetamine or a placebo - The cognitive control task had an easy or hard difficulty condition	- Dextroamphetamine improved cognitive control in healthy participants, as evidenced by increased d-prime, faster reaction times, and increased frontal P3a amplitude to non-target correct rejections. - Task difficulty moderated the effects of dextroamphetamine on EEG measures during target performance, with dextroamphetamine suppressing frontal theta power during easy target responses but increasing P3b amplitude during complex target trials. - The findings suggest a “gain-sharpening” effect of dextroamphetamine, where it boosted cognitive control processes under high-demand conditions but suppressed them under low-demand conditions.
Birch et al. (2022) [127]	16	- Prospective, single-arm, proof-of-concept study design - Participants with chronic pain recruited through clinics and word of mouth - Pre-intervention assessments, including pain, central sensitization, sleep, mood, and quality-of-life measures - Provision of Axon home-based EEG-neurofeedback system to participants - Eight-week neurofeedback training program with 32–48 self-administered sessions using game-based feedback to upregulate alpha brain activity	- A home-based EEG-neurofeedback intervention provided clinically significant pain relief for 8 out of 16 participants. - The intervention improved central sensitization symptoms, sleep quality, anxiety, and depression in a majority of participants. - The improvements in pain, central sensitization, sleep, anxiety, and depression were maintained at 4 and 12 weeks after the intervention.
Bismuth et al. (2020) [128]	32	- Randomized controlled pilot study design - A total of 32 patients were randomly assigned to one of two EEG-NFB protocols: - Increasing low-β(SMR)/high-β ratio (n = 16) - Increasing α(μ)/θ ratio (n = 16) - Twelve EEG-NFB sessions over 4 weeks - Clinical outcome measures collected before and 1 week after the sessions - Resting-state EEG recorded before and after each EEG-NFB session	
Blume et al. (2021) [129]	39	- Randomized controlled pilot study - A total of 39 adults with binge-eating disorder and overweight were randomly assigned to one of two EEG-neurofeedback interventions: - Food-specific neurofeedback targeting frontocentral beta and theta activity - General neurofeedback targeting slow cortical potentials - A waiting period of 6 weeks, followed by 6 weeks of 10 neurofeedback sessions (30 min each), and a 3-month follow-up - Outcomes measured included binge-eating episodes, eating disorder psychopathology, food craving, and executive functioning	- Both food-specific and general EEG-neurofeedback paradigms significantly reduced binge-eating episodes, eating disorder psychopathology, and food cravings in adults with binge-eating disorder. - Approximately one-third of participants achieved abstinence from binge-eating episodes after the neurofeedback treatments, with no difference in effectiveness between the two paradigms. - Both neurofeedback approaches were equally successful in modifying brain activity patterns, reducing relative beta, and enhancing relative theta power over fronto-central regions.
Bois et al. (2021) [130]	29	- The study had three groups: a control group, a neurofeedback (NF) group, and a motor-imagery (MI) group - Participants received pre- and post-intervention clinical assessments - The NF and MI groups received feedback on their brain activity through a videogame, where they had to regulate or modulate their brain activity to control the game - The NF group showed an increase in alpha-band power (8–12 Hz) in the Pz channel and across all channels, which is referred to as an “alpha rebound” and is consistent with previous research	- The neurofeedback group showed an increase in resting-state alpha wave activity following training sessions, which was associated with a clinically relevant reduction in PTSD symptom severity. - The study provides the first evidence supporting the use of low-cost neurofeedback as an effective treatment for PTSD in a developing-country setting.
Boonstra et al. (2016) [131]	20	- Sham-controlled, randomized, crossover design - Active tDCS at 2 mA for 15 min, sham involved brief ramp up and down - Resting-state EEG recorded for 8 min before, 15 min during, and 15 min after stimulation - Participants kept their eyes open and fixated on a target - Eight-electrode system used to deliver tDCS and record EEG, with the anode over left DLPFC and cathode over right fronto-orbital region	- Anodal tDCS of the left DLPFC using a high-current-density bi-frontal electrode montage resulted in an increase in power at lower frequencies and a decrease in power at higher frequencies. - Calculation of the mean EEG frequency revealed a generalized slowing of oscillations following both active tDCS and sham stimulation, with the effect being more pronounced after active tDCS. - In the sham condition, changes in mean EEG frequency were correlated with changes in subjective arousal, suggesting the slowing of resting-state EEG may be related to changes in arousal.
Bosch-Bayard et al. (2018) [132]	85	- Use of the elastic-net regression model for sparse classifier construction and variable selection - Evaluation of classifier performance using ROC measures, including a generalization to multiple ordered groups - Use of resampling techniques (repeated random subsampling) to ensure stability of the selected variables - Pre-selection of variables using the “indfeat” feature selection method - Stable estimation of the ROC using the empirical distribution of the ROC areas across multiple random subsamples	- The authors developed a novel methodology to identify stable and sparse classifiers that can predict the severity of learning disabilities (LD-NOS) based on quantitative EEG (qEEG) features. - Using this methodology, the authors identified a set of 20 qEEG features (biomarkers) that can effectively classify LD-NOS children into three subgroups with different levels of disability severity. - The identified qEEG biomarkers were associated with differences in cognitive and behavioral characteristics between the three LD-NOS subgroups, suggesting that the LD-NOS category may be too broad and that more specific EEG-based subtyping could guide tailored rehabilitation approaches.
Brown et al. (2020) [133]	10	- Within-subjects design with two visits: one with cannabis administration and one with placebo administration, in a counterbalanced order - EEG data was collected during neuro-cognitive tasks and a 45 min simulated driving task, with the final 10 min of the driving task being the focus of the analysis - Driving performance metrics, such as standard deviation of lane position (SDLP), extracted from the driving simulator and synchronized with the EEG data	- Participants showed significantly worse driving performance (increased lane position variability) and increased heart rate when intoxicated with cannabis compared to placebo. - EEG power in the theta frequency band (4–7 Hz) was significantly decreased during cannabis intoxication compared to placebo. - The decreased theta power during cannabis intoxication was negatively correlated with the driving performance metric, indicating the neurophysiological changes were associated with impaired driving.
Bryant et al. (2021) [134]	40	- A total of 40 PTSD patients participated in the study - Participants underwent a response inhibition (Go/No-Go) task while their brain activity was measured using fMRI and ERP - PTSD symptom severity was assessed using the Clinician-Administered PTSD Scale before and after nine sessions of trauma-focused cognitive behavioral therapy (TF-CBT) - The researchers analyzed the neural activity during the Go/No-Go task to see if it could predict changes in PTSD symptoms, specifically fear and dysphoric symptoms	- Reduced activation in the left precuneus and right superior parietal cortex during response inhibition predicted greater improvement in dysphoric (depressive) PTSD symptoms after trauma-focused cognitive behavioral therapy. - Shorter latency of the P3 event-related potential during response inhibition also predicted a greater reduction of dysphoric PTSD symptoms after therapy. - There were no significant predictors of changes in the fear symptoms of PTSD after therapy.
Bulletin et al. (2024) [135]	243	- Between-subjects design with four groups: schizophrenia, bipolar disorder, major depression, and healthy controls - Participants completed a visual perception task where stimuli appeared briefly - Measured attentional lapse rate and perceptual precision during the task - Recorded EEG activity, specifically in the alpha frequency band (8–13 Hz), and related it to behavioral performance	- The schizophrenia group had a higher rate of attentional lapses compared to the other groups. - The healthy control group showed the highest levels of pre-stimulus alpha activity when averaged across trials. - Fluctuations in pre-stimulus alpha activity on a trial-by-trial basis predicted the likelihood of making an error across all groups.
Burwell et al. (2014) [136]	410	- A total of 410 adult male participants completed a visual oddball task - Researchers measured phase-invariant evoked energy and inter-trial phase-locking in the delta and theta frequency bands at frontal and parietal scalp sites - These measures were investigated concerning externalizing disorders, including substance dependence, adult antisociality, and childhood disruptive disorders - The researchers hypothesized that weaker P3-related phase-locking would be associated with externalizing disorders and could explain previously observed reductions in P3 ERP amplitude	- Reductions in evoked energy and phase-locking in delta and theta frequency bands at the frontal and parietal regions were associated with greater odds of externalizing diagnoses. - Adding phase-locking measures to evoked energy improved the ability to predict externalizing diagnoses. - Reduced theta-band phase-locking partially mediated the effects of reduced the theta-band evoked energy on externalizing prediction.
Cao et al. (2018) [137]	27	- Thirty-two-channel EEG recordings from 27 participants - Participants drove on a simulated four-lane highway and were instructed to keep the car in the center lane - Random lane-departure events were induced, causing the car to drift left or right - Participants had to respond to steer the car back to the center lane - A new trial began 5–10 s after the previous trial ended	- The dataset collected from this sustained-attention driving task can be used to develop new methods for analyzing brain activity and detecting driver fatigue and drowsiness. - The dataset will be made publicly available and will be useful for researchers in neuroscience and brain–computer interface fields.
Casanova et al. (2020) [138]	38	- Participants: 19 children with ASD and 19 age- and gender-matched neurotypical children - Task: oddball task with Kanizsa figures to elicit gamma oscillations - Measurement: envelope analysis of demodulated waveforms for evoked and induced gamma oscillations - Intervention: 18 weekly sessions of low-frequency (1.0 Hz) transcranial magnetic stimulation (TMS) targeting the dorsolateral prefrontal cortex in the ASD group - Comparison: gamma oscillations were measured before and after the TMS intervention in the ASD group, and compared to the neurotypical group	- The ASD group showed higher magnitudes of evoked and induced gamma oscillations compared to the neurotypical group, especially in response to non-target stimuli, prior to receiving TMS treatment. - After receiving TMS treatment, the ASD group showed a significant reduction in gamma oscillations in response to task-irrelevant stimuli. - The ASD participants showed improved behavioral outcomes after receiving TMS treatment, including fewer errors and decreased irritability, hyperactivity, and repetitive behaviors.
Cavinato et al. (2019) [139]	24	- Intervention: transcranial direct current stimulation (tDCS) applied to the left dorsolateral prefrontal cortex - Participants: 12 patients with unresponsive wakefulness syndrome (UWS) and 12 patients with minimally conscious state (MCS) - Study design: each patient received 2 weeks of active tDCS and 2 weeks of sham tDCS - Measurements: EEG power spectra and coherence analysis performed before and after each tDCS session	- Active tDCS treatment led to increased power and coherence in the alpha and beta frequency bands in the frontal and parietal regions, as well as significant clinical improvements, in patients with minimally conscious state (MCS). - Patients with unresponsive wakefulness syndrome (UWS) only showed some local changes in the slow frequency bands in the frontal region, with no other significant effects. - No treatment effects were observed after the sham (placebo) tDCS condition.
Cecchi et al. (2023) [140]	161	- Recruitment of 81 healthy volunteers and 80 patients with schizophrenia, tested at four different sites - Each subject underwent two ERP/EEG testing sessions, which included the following: - Mismatch negativity paradigm - Auditory steady-state response paradigm at 40 Hz - Eyes-closed resting-state EEG - Active auditory oddball paradigm - Schizophrenia patients also completed the BAC, PANSS, and VRFCAT functional assessments - Standardized ERP/EEG instrumentation and methods were used, with an automated data analysis pipeline for near-real-time analysis	- Standardized methods and an automated analysis pipeline allowed for reliable collection and processing of high-quality ERP and QEEG data. - The ERP and QEEG measures showed good test–retest reliability. - Patients with schizophrenia exhibited deficits in ERP and QEEG measures compared to healthy volunteers, consistent with prior research. - Some ERP and QEEG measures correlated with functional assessments in the schizophrenia group.
Chen et al. (2019) [141]	20	- Twenty female participants with Mal de Debarquement Syndrome (MdDS), mean age 52.9 years and mean illness duration 35.2 months - Participants received 1 Hz inhibitory and 10 Hz excitatory repetitive transcranial magnetic stimulation (rTMS) over the dorsolateral prefrontal cortex (DLPFC) for 5 consecutive days - Resting-state fMRI and 126-channel EEG recordings were performed on days 1 and 5, with the rTMS sessions in between - EEG data were recorded using a 126-channel cap with sintered Ag/AgCl ring electrodes and an impedance limit of 10 kOhm	- Connectivity changes in the left medial frontal gyrus, primary visual cortex, and middle temporal gyrus correlated with symptom changes after rTMS treatment in MdDS patients. - Higher baseline connectivity in the primary visual cortex predicted better response to rTMS treatment. - Changes in EEG connectivity were related to changes in fMRI connectivity between the entorhinal cortex and inferior parietal lobule, suggesting network-level modulation.
Cheng et al. (2021) [142]	30	- Recruited 30 patients diagnosed with schizophrenia who were assigned to receive ECT - Collected 32-channel resting-state EEG data from participants 1 h before their first ECT session - Assessed positive and negative symptoms using the PANSS scale at baseline and after the eighth ECT session - Analyzed the EEG data using mutual information	- Higher assortativity (network connectivity) in the right temporal, right parietal, and right occipital cortex in the beta band is associated with better response to ECT in patients with schizophrenia. - Higher assortativity in the left frontal, parietal, right occipital cortex, and central area in the theta band is also associated with better response to ECT in patients with schizophrenia. - QEEG measures of brain network assortativity in the beta and theta bands could serve as potential biomarkers to predict ECT treatment response in patients with schizophrenia.
Conley et al. (2021) [143]	8	- Eight non-smoking participants with late-life depression completed EEG recordings at baseline and after 12 weeks of transdermal nicotine treatment - Nicotine was administered in a flexible dose escalation strategy, starting at 3.5 mg and increasing up to 21 mg over the 12 weeks - EEG was recorded using a 128-channel Geodesic sensor net, with a 3 min resting-state -recording and an auditory oddball task - The auditory oddball task presented 200 trials of 1000 Hz and 1500 Hz tones, with 70% standard and 30% target trials	- Twelve weeks of transdermal nicotine treatment in adults with late-life depression was associated with improved performance on an auditory oddball task, as evidenced by faster reaction times. - The nicotine treatment was associated with reduced beta desynchronization over the parietal cortex during the oddball task, and this change in beta power was correlated with improvements in depressive symptoms. - There were no significant changes in resting-state EEG power following the nicotine treatment.
Costa et al. (2019) [144]	33	- The study used a within-group design with an experimental group that received priming before neurofeedback training and a control group that received neurofeedback training without priming. - There were a total of 33 participants, with 16 in the control group and 17 in the experimental group. - The experimental group received a pre-training priming protocol (PRET) that used a within-subject ABA design, with “A” conditions involving mindfulness or guided imagery audio stimuli and “B” conditions involving an emotion questionnaire. - The neurofeedback training (NFT) protocol involved participants regulating their sensorimotor rhythm (SMR) in the 12–15 Hz range while simultaneously downregulating theta (4–7 Hz) and beta (21–35 Hz) frequencies.	- Priming subjects with mindfulness or guided imagery before neurofeedback training led to more substantial increases in self-regulation of the sensorimotor rhythm (SMR) compared to no priming. - However, the results were not conclusive or statistically significant due to overlapping standard deviations between the groups. - Further offline analysis is being conducted to validate the preliminary findings.
Dalkner et al. (2017) [145]	25	The methodology of this study involved a randomized controlled trial with 25 male patients with alcohol use disorder. The experimental group (n = 13) received 12 sessions of neurofeedback training over 6 weeks, focusing on enhancing alpha (8–12 Hz) and theta (4–7 Hz) brain waves using a visual feedback paradigm. The control group (n = 12) received treatment as usual without neurofeedback.	- The neurofeedback intervention significantly reduced avoidant personality accentuation in the experimental group compared to the control group. - There were also trending effects on reducing schizoid, schizotypal, and narcissistic personality accentuations. - The improvements in avoidant personality accentuation were maintained at the 5-month follow-up.
Davidson et al. (2023) [146]	10	- Three patients were implanted with bilateral Medtronic 3387 electrodes connected to a Percept implantable pulse generator, with the subgenual cingulum (SCC) targeted for electrode placement - Stimulation was delivered in a double-monopolar configuration using contacts 1 and 2, allowing local field potential (LFP) recordings between contacts 0 and 3 - Stimulation parameters were adjusted based on patients’ self-reported depressive symptoms, starting at 60 μs, 130 Hz, and 1 V and increasing as needed - LFP activity was recorded both with DBS on (weeks 3 and 24) and with DBS off (weeks 4 and 25) - Patients logged their mood states (neutral, happy, depressed, anxious) using a handheld programmer, and the device recorded 30 s of continuous LFP activity during these logged events, which was then analyzed in the frequency domain	- The only responder patient showed a distinct neural signature of negative affect states, characterized by reduced delta and increased alpha activity in the left hemisphere. - This neural signature was only observed at the 6-month time point and not in the earlier 3–5-week recordings. - No other frequency-band differences were found between positive and negative affect states or between early and late DBS.
Djonlagic et al. (2019) [147]	461	The study used in-home overnight polysomnography to collect EEG, EOG, EMG, respiratory, and other physiological data, which were then scored for sleep stages and sleep apnea/hypopnea events.	- The group that developed MCI or dementia showed higher EEG power across multiple frequency bands, including alpha and theta in NREM sleep and alpha and sigma in REM sleep, compared to the cognitively normal group, even when they were assessed to be cognitively normal at baseline. - These quantitative EEG changes preceded the clinical onset of cognitive decline by at least 5 years. - The results suggest that quantitative sleep EEG analysis may serve as a promising biomarker for imminent cognitive decline.
DuRousseau & Beeton (2014) [148]	18	- Thirty-two-channel EEG recordings from nine distressed couples before, during, and after a 90-day Imago Relationship Therapy program - Repeated measures t-test analysis to identify significant changes in EEG power in the alpha2, beta3, and gamma frequency bands in the prefrontal, frontal, and temporal-parietal cortices - Correlating these EEG changes with changes in relationship outcomes	- Significant reductions in EEG power in the alpha2, beta3, and gamma bands were observed in brain regions associated with executive function, default mode, and salience processing. - The observed changes in brain activity are consistent with the learning and implementation of the communication skills taught in the Imago Relationship Therapy program. - The changes in brain activity, specifically hemispheric lateralization, can be used as an indicator of behavioral changes in couples undergoing a relationship improvement program.
Eldeeb et al. (2021) [149]	21	- Used an EEG-based brain-computer interface (BCI) and an Affective Posner task to collect EEG data from 21 individuals with autism spectrum disorder (ASD) - Aimed to use the EEG data to differentiate between distress (LOSE) and non-distress (WIN) conditions in a game with deception - Analyzed the EEG features to classify the WIN, LOSE, and rest-EEG conditions, reporting the classification accuracies	- EEG features could differentiate between WIN (non-distress) and LOSE (distress) conditions with 81% accuracy. - EEG features could differentiate between LOSE (distress) and rest-EEG conditions with 94.8% accuracy. - EEG features could differentiate between WIN (non-distress) and rest-EEG conditions with 94.9% accuracy.
Engelbregt et al. (2016) [150]	25	- Randomized study design with an active E-NFT group and a sham/control group - Participants underwent 15 training sessions, each 45 min long - Resting-state EEG was measured at baseline (t1) and 3-year follow-up (t3)	- Real EEG-neurofeedback training predictably increased frontal beta activity, and this increase was maintained for 3 years after the training. - However, the EEG-neurofeedback training did not result in significantly improved cognitive performance. - The study demonstrates that EEG-neurofeedback can selectively modify EEG beta activity in both the short and long term.
Escolano et al. (2014) [151]	60	- Two groups: a neurofeedback (NF) group and a control group - The NF group received eight neurofeedback sessions over 4 weeks, with each session consisting of five trials of 4 min each - Cognitive assessments, including a working memory task (PASAT) and processing speed, were conducted before and after the training period - EEG was recorded using 16 electrodes placed according to the 10/10 system - The neurofeedback training targeted the increase of upper alpha power in the parieto-occipital region	- The neurofeedback group showed improved working memory performance and processing speed compared to the control group. - The neurofeedback group showed increased upper alpha power after the training, particularly in task-related activity. - The neurofeedback group showed increased current density in the alpha band in the subgenual anterior cingulate cortex.
Evans et al. (2015) [152]	124	- Within-subjects design with two sessions - Participants were heavy smokers - Participants smoked either very low nicotine or moderate nicotine cigarettes before 3 min of resting EEG - EEG activity in theta, alpha-1, beta-1, and beta-2 frequency bands were measured and compared between the two nicotine conditions	- Nicotine deprivation in heavy smokers is associated with greater power density in the theta and alpha-1 EEG bands compared to nicotine satiation. - Nicotine deprivation did not affect the power in the beta EEG bands in heavy smokers. - The increased slow-wave EEG activity during nicotine deprivation could be a reliable indicator of reduced cortical activity and associated cognitive deficits experienced during smoking withdrawal.
Fabio et al. (2016) [153]	34	- A total of 34 girls with Rett syndrome were divided into a training group (21 girls) and a control group (13 girls) - The training group received the following: - Short-term training (STT) session of 30 min - Long-term training (LTT) session of 5 days - Gaze data were recorded using an eye-tracker, and EEG data were recorded using wearable EEG equipment during the training sessions	- Participants showed a habituation effect, decreased beta activity, and increased right asymmetry after a short-term training session. - Participants looked faster and longer at the target and had increased beta activity and decreased theta activity, and a leftward asymmetry was re-established after long-term training. - Long-term cognitive training had a positive effect on both brain activity and behavioral measures in individuals with Rett syndrome.
Farzan (2019) [154]	20	- Open-label trial - Two-week duration - Bilateral theta burst stimulation (TBS) targeting the dorsolateral prefrontal cortex - A total of 20 youths with treatment-resistant depression (TRD) aged 16–24 years - Use of EEG and TMS-EEG neuroimaging to understand biological targets and predictors of response	- The study examined the feasibility, therapeutic potential, and biological targets of theta burst stimulation (TBS) in youth with treatment-resistant depression (TRD). - The study presented results from a 2-week open-label trial of bilateral TBS (left intermittent TBS and right continuous TBS) applied to the dorsolateral prefrontal cortex in 20 youths with TRD aged 16–24. - The study reviewed the use of multimodal neuroimaging such as EEG and TMS-EEG to understand the biological targets and identify predictors of response to rTMS therapy in youth.
Fink et al. (2023) [155]	56	- Randomized controlled trial with a waitlist-control-group and parallel-group design - Recruited participants from the West German Cancer Centre Essen, with specific inclusion and exclusion criteria - Assessments conducted at baseline, before the intervention, and after the 5-week intervention - Neurofeedback (NF) intervention using a modified EEG headset to provide feedback on alpha and theta/beta frequency bands The control group received a mindfulness-based group therapy intervention	- Both neurofeedback and mindfulness interventions significantly reduced affective symptoms like distress, depression, and anxiety in cancer patients. - Neurofeedback training specifically increased self-efficacy, which predicted improvements in quality of life. - Younger cancer patients benefited more from the neurofeedback intervention in reducing depression and anxiety despite starting with higher distress levels.
Gandelman-Marton et al. (2017) [156]	7	- Seven patients with mild Alzheimer’s disease were included - Patients received a 4.5-month (54-session) treatment combining repetitive transcranial magnetic stimulation (rTMS) and cognitive training - Quantitative EEG assessments were performed before treatment and after each treatment phase - Cognitive function was also assessed using the MMSE and Alzheimer’s Disease Assessment Scale–Cognitive Subscale and correlated with the EEG findings	- The study found a significant increase in delta wave activity in the temporal region of the brain after 4.5 months of repetitive transcranial magnetic stimulation (rTMS) interlaced with cognitive training in patients with mild Alzheimer’s disease. - The study also found non-significant increases in the power of various EEG frequency bands (alpha, beta, theta, delta) in different brain regions following the rTMS and cognitive training intervention. - Increases in alpha power in the frontal, temporal, and parieto-occipital regions were positively correlated with improvements in cognitive function as measured by the Mini-Mental State Examination (MMSE) at 6 weeks and 4.5 months.
Gangemi et al. (2023) [157]	30	- A total of 30 patients with chronic ischemic stroke were enrolled and divided into an experimental group and a control group - The experimental group received VR-based cognitive training, while the control group received conventional neurorehabilitation - EEG was used to measure changes in brain activity (neuroplasticity) in both groups after the training	- VR-based cognitive rehabilitation led to significant improvements in EEG-related neural measures, including increased alpha-band power in the occipital areas and increased beta-band power in the frontal areas. - The VR-based rehabilitation approach showed potential effectiveness in promoting neuroplastic changes in patients with chronic ischemic stroke. - No significant changes were observed in the theta-band power.
Gilleen et al. (2020) [158]	18	The study used a randomized, double-blind, placebo-controlled crossover design with 18 patients with schizophrenia. Roflumilast, a phosphodiesterase-4 inhibitor, was administered at 100 μg and 250 μg doses, and the effects on auditory steady-state response (early stage), mismatch negativity and theta (intermediate stage), and P300 (late stage) were measured using an electroencephalogram.	- Roflumilast, at a dose of 250 µg, significantly enhanced the amplitude of mismatch negativity and working memory-related theta oscillations in patients with schizophrenia, compared to placebo. - The results suggest that phosphodiesterase-4 inhibition with roflumilast can improve EEG markers of cognitive processing that are impaired in schizophrenia and that this effect is on intermediate-stage cognitive processing rather than early or late stages.
Goldstein et al. (2019) [159]	36	- Randomized controlled trial with three groups: MBSR, MBTI, and self-monitoring control - A total of 36 participants with chronic insomnia (>6 months) - Overnight polysomnography with six-channel EEG at baseline, post-treatment, and 6-month follow-up - EEG spectral power analysis focused on NREM sleep (excluding N1) in the C3/C4 channels - Examined within-group changes and relationships with self-report measures	- Mindfulness-based interventions (MBIs) led to increases in high-frequency NREM EEG power, specifically in the beta and gamma frequency ranges, compared to a control group. - The increases in NREM beta power were positively associated with improvements in mindfulness and negatively associated with reductions in insomnia severity. - The changes in high-frequency NREM EEG power were maintained at a 6-month follow-up for the MBI groups.
Guo et al. (2023) [160]	100	- A total of 60 patients with insomnia disorder (ID) and 40 good sleep controls (GSCs) were included - Resting-state EEG microstates, PSQI, and PSG data were collected - The 60 ID patients were randomly divided into active and sham rTMS treatment groups - An additional 90 ID patients received rTMS and were divided into optimal and suboptimal responder groups based on PSQI improvement - Baseline EEG microstates were used to build a machine learning model to predict the effects of rTMS treatment	- Patients with insomnia disorder had decreased occurrence and contribution of the class D EEG microstate, which was associated with longer sleep onset latency. - rTMS treatment partially reversed the abnormalities in EEG microstates in patients with insomnia disorder. - Baseline EEG microstate characteristics could accurately predict the therapeutic effect of rTMS treatment for insomnia disorder.
Hernandez et al. (2015) [161]	20	- A total of 20 healthy participants underwent EEG-neurofeedback training - The training protocol included the following: - Baseline trials (resting state) - Regulation trials with auditory feedback contingent on microstate D presence - A transfer trial - Response to neurofeedback was assessed using mixed-effects modeling - The researchers also examined the relationship between alpha power and microstate D contribution during the neurofeedback training	- All participants were able to increase the percentage of time spent producing microstate D, a pattern associated with positive symptoms in schizophrenia, through neurofeedback training. - The increase in microstate D was observed not only during the training sessions but also in the resting-state baseline and transfer conditions, suggesting a sustained change. - The training was specific to the attentional network, as evidenced by the negative correlation between alpha power and microstate D contribution.
Hill et al. (2021) [162]	60	- Used a TMS-EEG approach to measure oscillatory power in the brain - Examined oscillatory power in response to TMS over the DLPFC and M1 regions - Compared oscillatory responses between 38 MDD subjects and 22 healthy controls - Investigated changes in oscillatory responses in the MDD group after they received either magnetic seizure therapy (MST, n = 24) or electroconvulsive therapy (ECT, n = 14) as a form of convulsive therapy	- Individuals with major depressive disorder (MDD) exhibited increased oscillatory power in the delta, theta, and alpha frequency bands when transcranial magnetic stimulation (TMS) was applied to the dorsolateral prefrontal cortex (DLPFC), but not when applied to the motor cortex (M1), compared to healthy controls. - After receiving magnetic seizure therapy (MST), MDD patients showed reduced oscillatory power in the delta and theta bands when TMS was applied to the DLPFC. - After receiving electroconvulsive therapy (ECT), MDD patients showed reductions in the delta, theta, and alpha power when TMS was applied to the DLPFC and reduced delta and theta power when TMS was applied to the motor cortex (M1).
Hochberger et al. (2018) [163]	45	- Randomized controlled design, with participants assigned to either a treatment-as-usual (TAU) group or a TAU-plus-targeted-cognitive-training (TCT) group - Measured neurophysiological markers (mismatch negativity, MMN, and P3a) before and after an initial 1 h dose of TCT - Examined how changes in these neurophysiological markers after the initial 1 h dose predicted improvements in verbal learning and decreases in positive symptom severity after a full 30 h course of TCT	- Malleability (change from baseline) of MMN and P3a measures after an initial 1 h dose of auditory-based targeted cognitive training (TCT) predicted improvements in verbal learning and reductions in positive symptoms in patients with treatment-refractory schizophrenia. - Examination of MMN and P3a malleability after the first TCT session shows promise as a biomarker to predict clinical response to a full 30 h course of TCT and guide future treatment assignments.
Hochberger et al. (2019) [164]	52	- Randomized design with a treatment-as-usual (TAU) group and a TCT group - Measured EEG biomarkers of early auditory information processing (EAIP) at baseline and after 1 h of TCT - Used these EEG biomarkers to predict response to the full 30 h TCT intervention - Explored the use of EEG composite scores to identify patients most likely to benefit from TCT	- Baseline measures of theta oscillatory activity predicted improvements in overall cognitive function after 30 h of targeted cognitive training (TCT). - Decreases in theta activity in response to deviant stimuli after 1 h of TCT predicted improvements in verbal learning after 30 h of TCT. EEG-based composite scores demonstrated high sensitivity and specificity in identifying patients most likely to benefit from TCT.
Hunter et al. (2018) [165]	18	- A total of 18 clinically stable outpatients received rTMS treatment to the dorsolateral prefrontal cortex (DLPFC) - Treatment parameters were adjusted based on changes in symptom severity - Quantitative EEG (qEEG) recordings were taken at baseline and after 1 week of rTMS treatment, using a 21-channel dry-electrode headset - Analyses examined the relationship between changes in theta-band cordance after 1 week and patient- and physician-rated outcomes at 6 weeks	- Change in theta cordance in the central brain region during the first week of rTMS treatment predicted the percent change in self-reported depressive symptoms and whether the patient was considered improved or not improved at the end of 6 weeks of treatment. - The cordance biomarker remained significant when controlling for age, gender, and baseline severity.
Imperatori et al. (2023) [166]	8	- Between-subjects design with participants randomly assigned to view either natural (green) or urban (gray) images - Resting-state EEG recorded before and after the image viewing - Analysis focused on the “distress network” using eight pre-defined ROIs and the eLORETA software to compute Lagged Phase Synchronization (LPS) as a measure of functional connectivity	- Exposure to natural images, compared to urban images, was associated with increased positive emotions and subjective vitality. - Exposure to natural images was associated with decreased delta functional connectivity between the left insula and left subgenual anterior cingulate cortex, brain regions involved in emotional distress. - The decreased connectivity between the insula and subgenual anterior cingulate cortex is consistent with theories that natural exposure reduces physiological stress and lowers the effort required for voluntary attention.
Iosifescu (2020) [167]	1000	- The iSPOT-D study was an extensive randomized study with over 1000 participants with major depressive disorder (MDD) who were randomized to receive one of three antidepressant treatments: escitalopram, sertraline, or venlafaxine XR. - The study collected EEG biomarkers at baseline and during treatment. - Previous analyses of the iSPOT-D data found associations between baseline EEG parameters and treatment response, with some differences between the three antidepressant groups. - The Rajpurkar et al. study used a machine learning approach to re-analyze the iSPOT-D data and look for associations between specific baseline EEG features and changes in individual depressive symptoms.	- Specific baseline EEG features, such as occipital delta power and delta/alpha power, were associated with improvements in individual depressive symptoms like insight, energy, and psychomotor retardation. - A combination of clinical symptoms and EEG features may provide the most useful biomarkers for predicting antidepressant response rather than a single EEG biomarker alone. - While some EEG measures have been replicated, there is still uncertainty around how to define antidepressant response using these measures, and their clinical usefulness has not been fully established.
Iseger et al. (2017) [168]	1008	- Multi-center international study - Collected EEG data from 1008 MDD patients - Patients were randomized to receive one of three different antidepressant medications - Treatment response was defined as a >50% decline in the Hamilton Rating Score for Depression (HRSD17) - Analyzed changes in alpha and theta frequency connectivity in the DLPFC-DMPFC-sgACC network from pre- to post-treatment, comparing patients to controls and responders to non-responders	- Women exhibited higher alpha and theta connectivity compared to males, both pre-and post-treatment. - Depressed patients exhibited reduced theta, but not alpha, connectivity in the DLPFC-DMPFC-sgACC network compared to healthy controls. - A decrease in alpha connectivity in the DLPFC-DMPFC-sgACC network was found only in male responders to antidepressant treatment.
Israsena et al. (2020) [169]	35	- Multi-site pilot study conducted at five hospitals in Thailand - Participants were screened for cognitive function and assessed at baseline using CANTAB and EEG The intervention group underwent 20 sessions of 30-min neurofeedback-based brain training games over a 10-week period, targeting attention. - Cognitive and EEG assessments repeated after the training period The ethics review board approved the study, and all participants provided informed consent	- The neurofeedback-based brain training games led to significant improvements in visual memory, attention, and visual recognition in the elderly participants. - EEG data showed improvements in upper alpha activity in the occipital area, indicating improvements in cognitive function. - The study demonstrates the potential of practical neurofeedback-based training games for enhancing cognitive performance in the elderly population.
Janssen et al. (2016) [170]	112	- Randomized controlled trial (RCT) design with three parallel groups - Participants: 112 children aged 7–13 with ADHD diagnosis - Interventions: - Neurofeedback (NF) training for 30 sessions over 10 weeks - Physical activity (PA) training as a semi-active control group - Methylphenidate (MPH) medication in a double-blind placebo-controlled procedure - Outcome measures: event-related potentials (ERPs) related to response inhibition (N2 and P3) in a subset of 81 children at pre- and post-intervention	- Only the medication (methylphenidate) group showed specific improvements in brain function related to response inhibition, as measured by increased P3 event-related potential amplitude. - The improvements in the medication group were associated with increased activation in the thalamus and striatum, brain regions involved in response inhibition. - The results cast doubt on the efficacy and specificity of neurofeedback as a treatment for ADHD, as it did not demonstrate the same improvements as the medication group.
Kala et al. (2021) [171]	7	- Participants: seven children with ASD aged 4–7 years, with three in a waitlist control group and five in a follow-up group - Intervention: 16-week course of pivotal response treatment (PRT) targeting social communication skills and play, 8 h per week - EEG data collection: At four time points—16 weeks before the start of treatment (waitlist only), pre-treatment, post-treatment, and 16 weeks after the end of treatment (follow-up) - EEG paradigm: Participants viewed 146 dynamic trials of 70 computer-generated faces with neutral and fearful expressions - EEG data analysis: 128-channel EEG recorded, data filtered, segmented, baseline corrected, and re-referenced	- Significant reductions in N170 latency, a neural marker of face processing, were observed in children with ASD after 16 weeks of pivotal response treatment (PRT). - There were no significant changes in the P100 component, which reflects low-level visual processing, suggesting the changes were specific to face processing rather than general visual processing. - The changes in N170 latency were stable during a 16-week follow-up period after treatment, and there were no changes in the 16 weeks before treatment, suggesting the changes were meaningful and specific to the treatment.
Karch et al. (2014) [172]	16	- Participants: eight adults with ADHD and eight matched healthy controls - Experimental task: auditory go/no-go task with a voluntary selection condition - Data acquisition: simultaneous EEG and fMRI recording during the task - Data analysis: single-trial coupling of EEG and fMRI data, measuring N2 and P3 ERP components and comparing ADHD patients and healthy controls	- ADHD patients showed reduced N2-related brain activity, especially in frontal regions, compared to healthy controls during voluntary decision-making, suggesting early deficits in frontal brain function. - However, P3-related brain responses did not differ significantly between ADHD patients and healthy controls, indicating that later stages of information processing may be less affected in ADHD.
Kavanaugh et al. (2023) [173]	28	- A total of 28 adults with major depressive disorder (MDD) participated - Participants completed self-report questionnaires (Frontal Systems Behavior Scale) and resting-state EEG recordings before and after receiving a course of repetitive transcranial magnetic stimulation (rTMS) therapy - EEG data were analyzed to calculate the rate, power, duration, and frequency span of beta oscillatory events, as well as events in delta/theta and alpha bands - The researchers examined the relationship between pre-treatment beta event rates at specific EEG electrode locations (F3, Fz, F4, Cz) and the subsequent improvement in executive dysfunction (EDF) after rTMS treatment while controlling for improvement in depressive symptoms	- A lower rate of beta events in the fronto-central regions of the brain before rTMS treatment was associated with greater improvement in executive dysfunction after the rTMS treatment. - A decrease in beta event rate in the frontal midline region from before to after rTMS treatment was also associated with greater improvement in executive dysfunction. - The findings were specific to the beta frequency band and not observed in other frequency bands.
Kim et al. (2022) [174]	48	- A total of 48 PTSD patients were enrolled, with 23 males and a mean age of 50.81 ± 11.60 years - Patients received 10 sessions of 2 mA tDCS stimulation for 20 min, with the anode over F3 and cathode over F4 - Sixty-two-channel EEG data were recorded for 3 min before and after tDCS, and power spectral density (PSD) was calculated for five frequency bands (delta, theta, low alpha, high alpha, and beta) - An SVM machine learning model was used to classify responders and non-responders based on the pre-treatment PSD, achieving an AUC of 0.93 using a multichannel approach	- Changes in theta and beta frequency bands in the EEG data were key indicators of the clinical effects of tDCS treatment for PTSD symptoms. - Responders to tDCS treatment showed a decrease in theta and beta power, while non-responders showed an increase, and these differences were related to improvements in PTSD symptoms. - The study developed a machine learning model that could predict tDCS treatment response in PTSD patients with high accuracy (AUC = 0.93) using pre-treatment EEG data.
Kober et al. (2015) [175]	64	- Three groups: - Eleven stroke patients received SMR neurofeedback training - Six stroke patients received upper alpha neurofeedback training - Seven stroke patients received “treatment as usual” control - A total of 40 healthy controls also received neurofeedback training - Pre-post design, with cognitive function assessed before and after neurofeedback training - Specific cognitive outcomes measured included verbal short-term memory, verbal long-term memory, visuospatial short-term memory, and working memory	- About 70% of both stroke patients and healthy controls showed improvements in verbal short-term and long-term memory with neurofeedback training, regardless of the specific protocol used. - The SMR neurofeedback protocol led to specific improvements in visuospatial short-term memory in stroke patients, while the upper alpha protocol led to particular improvements in working memory. - The neurofeedback training effects on memory were more potent than the effects of traditional cognitive training methods in stroke patients.
Köhler-Forsberg et al. (2020) [176]	100	- Non-randomized, open-label clinical trial of 100 untreated patients with moderate to severe depression - Patients will receive SSRI treatment (escitalopram, with the option to switch to duloxetine) - Assessments at baseline, during, and after 12 weeks of treatment, including PET, fMRI, EEG, cognitive tests, and peripheral biomarkers A subset of patients will undergo additional neuroimaging and EEG assessments after 8 weeks of treatment	
Kolk et al. (2016) [177]	52	- Randomized, waitlist-controlled trial with 52 participants with chronic PTSD - Participants were randomly assigned to either a neurofeedback (NF) group or a waitlist (WL) control group - Assessments conducted at four time points: baseline, week 6, post-treatment, and 1-month follow-up - Assessment measures included the Traumatic Events Screening Inventory, Clinician-Administered PTSD Scale (CAPS), Davidson Trauma Scale (DTS), and Inventory of Altered Self-Capacities (IASC) - NF training protocol involved 24 sessions over 12 weeks, with the active site at T4 and the reference site at P4	- Neurofeedback training produced significant improvements in PTSD symptomatology compared to the waitlist control group. - Neurofeedback also led to significant improvements in affect regulation capacities. - The effect sizes of neurofeedback were comparable to the most effective evidence-based treatments for PTSD.
Koller-Schlaud et al. (2021) [178]	45	The study used a naturalistic design to assess participants with major depression in psychiatric in- and outpatient hospital settings. Participants had to meet several inclusion criteria, including a diagnosis of major depression, being right-handed, and having a MADRS score greater than 19. EEG was recorded at baseline (T0) and about 1 week after the initiation of treatment (T1) while participants completed a task involving the presentation of happy and sad facial expressions.	- Responders showed a differential change in frontal alpha-1 asymmetry in the first week of treatment depending on the presented stimuli valence (happy vs. sad facial expressions). In contrast, non-responders did not show this pattern. - Reduction in depressive symptoms was generally associated with an increase in alpha-1 asymmetry in the happy-face condition and with a decrease in the sad-face condition across groups. - The study did not find significant group differences in occipital alpha-1 and alpha-2 asymmetry or frontal midline theta activity at baseline, nor were group differences observed regarding changes of these parameters from baseline to 1 week after treatment initiation.
Kratzke et al. (2020) [179]	15	- Prospective study design approved by an institutional review board - A total of 15 surgical residents with burnout and depression were enrolled - A total of 10 residents with more severe symptoms received 8 weeks of neurofeedback treatment, while five others with less severe symptoms were used as controls - Cognitive workload was assessed via EEG during a working memory task before and after the neurofeedback intervention - ANOVA was used to test for significant differences in cognitive workload changes between the treatment and control groups	- The treatment group that received neurofeedback showed a significant improvement in cognitive workload, as measured by EEG, compared to the control group. - There was a significant correlation between the number of neurofeedback sessions and the average improvement in various growth areas, such as sleep and stress. - The residents demonstrated high levels of burnout and depression, which were associated with EEG patterns indicative of post-traumatic stress disorder, and the neurofeedback treatment led to a notable change in cognitive workload, suggesting a return to a more efficient neural network.
Lackner et al. (2016) [180]	25	- Randomized controlled trial design with an experimental group (n = 13) and a control group (n = 12) The experimental group received 12 sessions of visual neurofeedback training over 6 weeks, targeting enhancement of alpha (8–12 Hz) and theta (4–7 Hz) frequency bands The control group received a standard treatment program but no additional neurofeedback intervention. - Outcome measures included changes in EEG-band power as well as several clinical variables related to mental health and alcohol use	- The experimental group showed a trend-level increase in resting-state alpha and theta power after the visual neurofeedback training. - Patients in the experimental group reported feeling increased control over their brain activity during the neurofeedback training. - The experimental group showed improvements in several clinical measures (depression, psychiatric symptoms, coping, post-traumatic growth) from pre- to post-test, while the control group did not.
Lavanga et al. (2020) [181]	92	- Recruited a prospective cohort of 92 preterm infants from the NICU at University Hospitals Leuven in Belgium - Inclusion criteria were preterm infants born before 34 weeks gestational age or with birth weight < 1500 g - Exclusion criteria included parental age < 18, parental medical conditions, lack of Dutch/English proficiency, and significant congenital/neurological abnormalities in the infant - Collected physiological data, including EEG using nine electrodes and ECG to derive heart rate variability (HRV) - Quantified neonatal procedural pain exposure as the sum of defined skin-breaking procedures (SBPs) based on a neonatal stress scale	- A high number of early skin-breaking procedures (SBPs) in premature infants is associated with a more discontinuous and dysmature EEG pattern. - A high number of early SBPs is associated with higher heart rate variability (HRV) in premature infants. - These associations between early SBPs, dysmature EEG, and higher HRV were found in both the whole dataset and the subset of extremely preterm infants (GA ≤ 29 weeks).
Leem et al. (2020) [182]	46	- Randomized, waitlist-controlled, assessor-blinded clinical trial - A total of 46 PTSD patients were randomly assigned 1:1 to treatment and control groups The treatment group received 50 min neurofeedback sessions twice per week for 8 weeks (16 sessions total) - Quantitative EEG is used to monitor participants’ physiological functions and brain waves	
Liu et al. (2023) [183]	50	- Retrospective case-control study design - EEG measures, including ERPs, oscillations, and functional connectivity during a Go/NoGo task - Recruitment of patients with UFLI, BFLI, and healthy controls - EEG data recorded using a 32-channel system with specific parameters - Offline preprocessing, including re-referencing, artifact correction, and analysis of time-domain and time-frequency features	- Patients with BFLI showed deficits in conflict monitoring, cognitive control processes, and functional connectivity in posterior, dorsal, frontoparietal, and midfrontal-related networks. - Patients with UFLI showed deficits in response decision and motor preparation but compensatory increases in functional connectivity in the uninjured hemisphere and across networks. - The findings suggest that the nodes of the affected networks could serve as targets for neuromodulation interventions in patients with frontal lobe injury.
Lundstrom et al. (2019) [184]	21	- Patients underwent intracranial EEG (iEEG) monitoring and were offered a 1–4 day trial of continuous electrical stimulation targeting the seizure onset zone (SOZ) and surrounding tissue - For 13 of the 21 patients, four 15-min epochs of iEEG data were analyzed—two from the first/second day and two from one of the last two days of the stimulation trial - Interictal epileptiform discharges were quantified using a validated spike detector, excluding discharges at the stimulation frequency and harmonics to account for artifacts - Power spectra and power in delta (1–4 Hz), alpha (8–13 Hz), and beta (13–20 Hz) frequency bands were calculated using Welch’s method and zero-phase Butterworth filtering	- Decreases in delta power and increases in alpha and beta power during trial stimulation were correlated with improved long-term clinical outcomes for patients receiving chronic subthreshold cortical stimulation (CSCS) treatment. - A majority of patients (63%) experienced seizure-free periods of at least 3 months, with 40% being seizure-free for at least 12 months. - The responder rate (at least 50% seizure reduction) was 89%, and the median reduction in seizure frequency was 100%.
Mangia et al. (2014) [185]	10	- Five healthy subjects and five patients with disorders of consciousness participated - The experiment had two trials: an Imagery Trial with hand and foot movement imagery tasks and a pre-communication Trial with yes/no questions answered through imagery - EEG data were recorded from 31 electrodes, and power spectral density features in different frequency bands were extracted for analysis	- The study achieved high classification accuracy (over 80%) in distinguishing between two mental imagery tasks (hand vs. foot movement) in both healthy subjects and patients with disorders of consciousness. - The study also achieved high accuracy (over 80%) in detecting answers to simple yes/no questions using the same EEG-based approach. - The optimal subset of electrodes for classification varied across subjects and sessions, suggesting the need for subject-specific and session-specific optimization.
Marceglia et al. (2016) [186]	7	- Transcranial direct current stimulation (tDCS) with anodal and cathodal stimulation over the temporal–parietal areas in seven Alzheimer’s disease patients - EEG recording using a 21-electrode setup at baseline and 30 min after tDCS - Word recognition task performed at the same time points as the EEG recordings - Analysis of EEG spectral power and coherence in different frequency bands - Correlation of tDCS-induced EEG changes with performance on the word recognition task	- Anodal tDCS over the temporo-parietal area increased high-frequency power and coherence in Alzheimer’s disease patients, which correlated with improved performance on a working memory task. - Cathodal tDCS had a non-specific effect of decreasing theta power without any correlation to memory task performance. - The increase in high-frequency power after anodal tDCS was directly correlated with an increase in nitric oxide levels.
Marlats et al. (2019) [187]	60	- Randomized controlled trial (RCT) design - Single-blind protocol - Two groups: intervention group and control group - Intervention: 30 sessions of either sensorimotor/delta-ratio or beta1/theta-ratio neurofeedback training - Outcome measures: neuropsychological assessments, questionnaires, and EEG, measured at baseline, immediately after the intervention, and 3-month follow-up	
Martínez-Briones et al. (2021) [188]	18	- EEG recordings in 18 children with learning disorders (ages 8–11) - Participants performed a Sternberg-type working memory task - Ten participants received 30 sessions of a neurofeedback (NFB) treatment - Eight participants received 30 sessions of a placebo–sham treatment - Behavioral performance and EEG power spectrum were analyzed before and after the treatments	- The NFB group showed faster response times in the working memory task after the treatment. - The NFB group exhibited decreased theta power and increased beta and gamma power at frontal and posterior brain sites after the treatment. - The authors explain these findings as the NFB treatment improving the efficiency of neural resource management, maintenance of memory representations, and subvocal memory rehearsal in children with learning disorders.
Mayeli et al. (2024) [189]	13	- A total of 13 individuals with bipolar disorder (10 type I, 3 types II) underwent three TMS-EEG and TBS sessions with 1 week between sessions - Participants completed a delay discounting task before and after TBS while EEG was recorded - Measures included the following: choice behavior (immediate vs. delayed choices), EEG beta power in left vlPFC, right dlPFC, and left somatosensory cortex, and coherence between left vlPFC and right dlPFC - EEG data were preprocessed, divided into epochs, and analyzed using Morlet wavelet for time-frequency analysis - Coherence analysis focused on high-beta-band coherence between left vlPFC and right dlPFC	- Continuous theta burst stimulation (cTBS) of the left primary somatosensory cortex (SOM) led to a reduction in the proportion of immediate reward choices in individuals with bipolar disorder. - cTBS of the SOM increased high-beta-band coherence between the left ventrolateral prefrontal cortex (vlPFC) and right dorsolateral prefrontal cortex (dlPFC) during trials where immediate rewards were chosen. - The double-blind, crossover experimental design involving three different theta burst stimulation (TBS) interventions was feasible and safe in individuals with bipolar disorder.
McMillan et al. (2019) [190]	30	- Randomized, double-blind, active placebo-controlled crossover trial - A total of 30 participants with major depressive disorder (MDD) - Simultaneous EEG and fMRI recording during infusion of ketamine or active placebo (remifentanil) - Measured depression symptoms using the Montgomery–Asberg depression rating scale - Analyzed BOLD signal changes in the brain using pharmacological MRI (phMRI), including in the anterior cingulate, medial prefrontal cortex, and subgenual anterior cingulate cortex - Analyzed EEG data, examining changes in power across different frequency bands (theta, beta, gamma, delta, and alpha) and their time courses	- fMRI analyses showed increased BOLD signals in the anterior cingulate and medial prefrontal cortices and a decreased BOLD signal in the subgenual anterior cingulate cortex that was sensitive to noise correction. - EEG spectral analysis showed increased theta, high-beta, low- and high-gamma power, and decreased delta, alpha, and low-beta power, with differing time-courses. - Low-low-beta and high-gamma power time courses explained significant variance in the BOLD signal, and the variance explained by high-gamma power was associated with non-response to ketamine.
Mondino et al. (2020) [191]	92	The study was a retrospective evaluation of Beck Depression Inventory (BDI) scores for 92 patients with a medication-resistant unipolar major depressive episode (MDE) who received 2–6 weeks (10–30 sessions) of daily active 1 Hz-rTMS combined either with active venlafaxine or with placebo venlafaxine. The stimulation protocol involved 360 pulses per session, delivered in six bursts of 60 s separated by 30 s of rest, at an intensity of 120% of the resting motor threshold. The 13-item self-rated BDI was used to assess depressive symptoms at baseline and weekly, with the early-improvement assessment point defined at week 1 and the post-treatment score defined as the last assessment point between weeks 2 and 6.	- Lack of early (after five sessions) improvement of at least 15% in self-rated BDI scores can predict non-response to 1 Hz rTMS over the right DLPFC with 79–85% negative predictive value. - This predictive ability is not affected by whether the rTMS is combined with active or placebo venlafaxine.
Murias et al. (2018) [192]	25	- Phase I, single-center, open-label trial of a single intravenous infusion of autologous umbilical cord blood in 25 children with ASD aged 2–6 years - Participants had to have an available autologous cord blood unit meeting specific criteria - Cord blood was thawed, washed, and administered via peripheral IV with premedication and monitoring - Participants were evaluated at baseline, 6 months, and 12 months post-infusion, including clinical assessments and EEG recordings during social and non-social video viewing - EEG data were preprocessed and analyzed for absolute and relative power in theta, alpha, and beta frequency bands across brain regions	- Significant changes in EEG spectral characteristics were found 12 months post-infusion, characterized by increased alpha and beta power and decreased theta power. - Higher baseline posterior EEG beta power was associated with greater improvement in social communication symptoms, suggesting EEG beta power as a potential biomarker to predict treatment response.
Oakley et al. (2022) [193]	224	- Used resting-state EEG data - Developed a machine learning algorithm (MLA) with two steps: 1. Applied the directed phase lag index (DPLI) to the EEG data to measure phase synchronization between brain regions 2. Searched the DPLI matrix for patterns of features that could predict response to Sertraline or placebo treatment	- The study developed a machine learning algorithm that could predict an individual’s response to the antidepressant Sertraline or placebo treatment with over 80% accuracy using resting-state EEG data. - The specific feature patterns extracted from a measure of phase synchronization between brain regions (DPLI) were predictive of an individual’s response to both Sertraline and placebo treatment. - The researchers suggest the developed algorithm could be a useful clinical tool to help predict an individual’s response to antidepressant treatment, which could lead to more effective and efficient treatment of major depressive disorder.
Ouyang et al. (2018) [194]	20	- Enrolled 20 children with epilepsy - Classified participants into “effective” (>50% reduction in seizures) and “ineffective” (<50% reduction in seizures) groups - Collected EEG data before and 1–3 months after starting/changing antiepileptic drugs - Performed quantitative EEG (QEEG) analysis on the EEG data - Used six specific QEEG features to classify participants into effective and ineffective groups - Used follow-up EEG data to test the accuracy of the QEEG analysis	- Six EEG feature descriptors were identified that could accurately classify patients as having an effective or ineffective response to antiepileptic drugs, with a 100% precision rate. - The method maintained an 83.3% accuracy when tested on follow-up EEG data.
Palanca et al. (2018) [195]	15	- Randomized crossover design with three treatment conditions (etomidate + ECT, ketamine + ECT, ketamine + sham ECT) repeated over 2 weeks - High-density EEG, clinical EEG, and monitoring of responsiveness to verbal commands to assess cognitive and neurophysiological recovery - Primary outcomes include cognitive task performance, time to return of responsiveness and presence of delirium - Secondary outcomes include EEG-based measures of the seizures and postictal period	- The main objectives of this study protocol are to investigate the recovery of cognitive and neurophysiological function following right-unilateral electroconvulsive therapy (ECT) in individuals with treatment-resistant depression. - The study aims to determine how the reconstitution of different cognitive domains varies in rate and order depending on the presence of electrically induced seizures. - The study will also assess the relationship between postictal delirium and delayed restoration of baseline cognitive function, as well as compare the sequence of cognitive recovery to that seen after isoflurane anesthesia.
Pan et al. (2024) [196]	113	- Participants were selected based on inclusion criteria - Depression was assessed using the Zung Depression Scale - Cognitive function was evaluated using the P300 event-related potential - EEG data were collected using the NeuroScan SynAmps RT EEG system - Statistical analyses included *t*-tests, Wilcoxon rank-sum tests, chi-square tests, multiple linear regression, and multiple logistic regression	- Depressed patients showed greater theta power in the left frontal lobe compared to the right, while the opposite was true for healthy controls. - The frontal theta asymmetry (FTA) in the F3/F4 regions was associated with the presence of depression and changes in cognitive function. - FTAs can be used to assess the severity of depression and identify cognitive impairment in depressed patients.
Park et al. (2022) [197]	55	- Non-equivalent control group pre-test–post-test design - A total of 55 late adolescent participants in experimental and control groups - A total of 10 sessions of EEG biofeedback training over 5 weeks for the experimental group - Quantitative EEG measurements taken before and after the intervention - Data analyzed using Shapiro–Wilk test, Wilcoxon tests, *t*-tests, and fast Fourier transform for EEG spectral analysis	- EEG biofeedback training significantly improved emotion regulation in late adolescents, including reduced anxiety about COVID-19 infection, improved mood repair, and enhanced self-regulation ability. - EEG biofeedback training led to improvements in brain homeostasis, specifically enhancing sensory–motor rhythm and inhibiting theta waves. EEG biofeedback training has the potential to be an effective nursing intervention for late adolescents to manage emotional distress during the COVID-19 pandemic.
Parmar et al. (2021) [198]	12	- Randomized, double-blind, sham-controlled, crossover clinical trial design - Anodal high-definition transcranial direct current stimulation (aHD-tDCS) administered at 1.693 mA for 20 min over the right ventrolateral prefrontal cortex (vlPFC) on four consecutive days - Participants underwent both active and sham aHD-tDCS conditions, with a 3-week interval between conditions - Final sample of 12 participants with ASD (7 males, mean age = 25.08 ± 7.20 years)	- There was no significant effect of active aHD-tDCS over the right vlPFC compared to sham stimulation on measures of cognitive flexibility in individuals with autism spectrum disorder. - aHD-tDCS was generally safe and well-tolerated, with only minor and transient side effects reported. - The study design was feasible, with excellent visit compliance and only two participants withdrawing before the second condition.
Perez et al. (2022) [199]	152	- Considered only randomized, double-blind, sham-controlled trials involving participants with at least one internalizing disorder diagnosis - Searched multiple databases and clinical trial registries to identify eligible studies, with no language restrictions - Two independent reviewers screened titles/abstracts and full-text articles for eligibility, with a third reviewer resolving any disagreements - Data extraction was performed by one reviewer and independently verified by another - Risk of bias was assessed using the Cochrane Risk-of-Bias tool version 2 (RoB 2.0)	- The available evidence suggests EEG-neurofeedback may have specific effects in treating some internalizing disorders like PTSD and OCD, but the evidence is very limited. - The small sample sizes and heterogeneity across the few eligible trials precluded a robust quantitative synthesis. - One eligible trial had not published its results at the time of the review.
Pérez-Elvira et al. (2021) [200]	40	- Enrolling 45 adolescents aged 10–15 years with learning disabilities who met specific criteria - Collecting 19-channel EEG data at 256 Hz for 3–5 min per participant - Analyzing the EEG data using fast Fourier transform to measure absolute and relative power in frequency bands - Providing 10 sessions of 30 min Live Z-Score Training Neurofeedback (LZT-NF) twice a week, with participants able to choose the visual/auditory feedback type	- The individual alpha peak frequency (i-APF) can be used as a biomarker to identify optimal responders to Live Z-Score Training Neurofeedback (LZT-NF) in adolescents with learning disabilities. - Participants with normal i-APF (ni-APF) were more likely to show improvements in QEEG metrics and cognitive/learning outcomes compared to those with low i-APF (li-APF), who were considered non-optimal responders to the LZT-NF intervention.
Pinter et al. (2021) [201]	14	- A total of 14 patients with multiple sclerosis (pwMS) underwent 10 neurofeedback training sessions over 3–4 weeks using a telerehabilitation system - Participants were divided into two groups: seven “responders” who learned to self-regulate their sensorimotor rhythm (SMR) through visual feedback and showed cognitive improvement and seven “non-responders” who did not - Diffusion-tensor imaging (DTI) and resting-state functional MRI (rs-fMRI) were performed on all participants before and after the neurofeedback training	- Participants who successfully learned to self-regulate their sensorimotor rhythm (SMR) through neurofeedback training showed increased fractional anisotropy (FA) and functional connectivity (FC) in the salience network (SAL) and sensorimotor network (SMN) compared to non-responders. - Cognitive improvement in the responder group correlated with increased FC in the SAL and showed a trend towards correlation with increased FA.
Powell et al. (2014) [202]	14	- Part of a larger clinical trial on tDCS for depression treatment - Included patients diagnosed with major depressive disorder based on clinical interviews and depression scales - Used a double-blind, sham-controlled, crossover design where participants received both active and sham tDCS - Employed a visual working memory (VWM) task with varying levels of difficulty to assess cognitive performance - Collected 64-channel EEG data using standard electrode placement	- Active tDCS to the left dorsolateral prefrontal cortex (DLPFC) resulted in a significant reduction in the N2 amplitude during the retrieval phase of a visual working memory (VWM) task, compared to sham stimulation. - Active tDCS also resulted in a significant reduction in frontal theta power during the retrieval phase of the VWM task, compared to sham stimulation. - Active tDCS increased occipito-parietal alpha desynchronization during the maintenance phase of the VWM task, compared to sham stimulation.
Prinsloo et al. (2017) [203]	71	- Participants were recruited from a cancer center database and referrals, diagnosed with CIPN by oncologists - The NFB group received 20 sessions of neurofeedback over 10 weeks, while the control group was on a waitlist - EEG data were collected and used to guide the neurofeedback training protocols - Participants played a 45 min game where they received feedback for matching certain EEG thresholds	- The neurofeedback (NFB) group demonstrated significantly greater decreases in the worst pain, average pain, and pain interference compared to the waitlist control (WLC) group. - The NFB group showed increases in alpha activity and decreases in beta activity compared to the control group. - Decreases in worst pain were correlated with reductions in beta power in several brain regions.
Rajeswaran & Bennett (2019) [204]	60	- Randomized controlled trial design - A total of 60 participants were recruited and randomly allocated to an intervention (neurofeedback training) or waitlist control group The intervention group received 20 sessions of alpha/theta neurofeedback training, each lasting 40 min and held 5–6 times per week. - Clinical and neuropsychological assessments, as well as serum cortisol measurements, were conducted pre- and post-intervention	- EEG-neurofeedback training (EEG-NFT) was effective in improving cognitive functions, reducing symptoms, decreasing cortisol levels, and improving quality of life in patients with traumatic brain injury (TBI). - The improvements were corroborated by both the patients and their significant others after the neurofeedback training.
Ravan et al. (2015) [205]	113	- EEG recordings of oddball auditory evoked potentials were collected from 66 healthy volunteers (HVs) and 47 schizophrenic (SCZ) adults, both before treatment (BT) and after treatment (AT) with the drug Clozapine (CLZ) - SCZ subjects were divided into “most-responsive” and “least-responsive” groups based on a 35% improvement criterion after CLZ treatment - Brain source localization (BSL) was used to extract source waveforms from specified brain regions in the EEG signals - Machine learning (ML) methods were then applied to the source waveform signals to identify a set of features that could distinguish SCZ from HVs BT, distinguish SCZ BT vs. AT in the most responsive group, distinguish least responsive SCZ from HVs AT, and no longer distinguish most responsive SCZ from HVs AT	- A set of EEG-derived features was identified that could distinguish schizophrenic subjects from healthy volunteers before treatment and could also distinguish schizophrenic subjects before and after Clozapine treatment. - These EEG features normalized in schizophrenic subjects who responded well to Clozapine treatment, suggesting they are related to the functioning of the default mode network in the brain. - The study proposes that the machine learning approach used could be a powerful tool for understanding the effects of psychiatric medications and could help develop new antipsychotic drugs.
Ricci et al. (2020) [206]	8	- Randomized, sham-controlled, double-blind, crossover study design - Eight healthy male participants aged 25–45 years - Sixty-minute intervention of either real tVNS applied to the left external acoustic meatus or sham stimulation applied to the left ear lobe - Five-minute EEG recordings taken before and sixty minutes after the intervention	- Real transcutaneous vagus nerve stimulation (tVNS) increased the duration of microstate A and the power in the delta frequency band of the EEG in healthy subjects. - The study confirmed that tVNS is an effective method for stimulating the vagus nerve and that the effects can be measured using quantitative EEG analysis. - Further research is warranted to explore the clinical implications of these findings and identify potential biomarkers for tVNS therapy in neuropsychiatric disorders.
Salle et al. (2016) [207]	21	- Randomized, double-blind, placebo-controlled crossover design - Administration of a sub-anesthetic dose of ketamine or saline placebo - Resting-state EEG recording from 28 scalp electrodes with participants’ eyes closed - Use of the eLORETA method to estimate the cortical sources of the EEG activity	- Ketamine administration in healthy humans produced schizophrenia-like changes in resting-state EEG activity, including increased gamma and reduced alpha, theta, and delta activity. - The EEG changes induced by ketamine were associated with increased dissociative symptoms, particularly depersonalization. - The findings support the hypothesis that NMDA receptor hypofunction and associated pathological brain oscillations may contribute to the emergence of perceptual/dissociative symptoms in schizophrenia.
Schabus (2017) [208]	500	- Neurofeedback training (NFT) protocol with eight blocks of 5 min each, including two “transfer blocks” without immediate feedback - Double-blind study design, in contrast to a previous single-blind study - Participants were older (mean age 38.6 years) and had more severe insomnia compared to previous studies	- Even misperception insomniacs showed unaltered EEG activity after neurofeedback training, contradicting earlier positive findings from the authors’ laboratory. - The authors highlight limitations of their earlier single-blind study, which placebo effects may have influenced due to increased social support in the neurofeedback condition. - The authors argue that it is difficult to imagine how neurofeedback can lead to consistent improvements in various disorders and symptoms without detectable changes in brain activity over time.
Schultheis et al. (2022) [209]	474	- Randomized, double-blind, placebo-controlled, parallel-group Phase II study - Patients randomized to receive one of four doses of iclepertin (2, 5, 10, or 25 mg) or placebo for 12 weeks - EEG data collected from a subgroup of 79 patients at baseline and end of treatment - EEG parameters measured were mismatch negativity (MMN), auditory steady-state response (ASSR), and resting-state gamma power, and their correlations with clinical assessments were analyzed	- At baseline, the mismatch negativity (MMN) and auditory steady-state response (ASSR) EEG parameters exhibited consistent correlations with clinical assessments of schizophrenia, indicating their potential as neurophysiological biomarkers of the disorder. - ASSR measures were positively correlated with cognitive performance, while MMN amplitude was positively correlated with symptom scales. - However, the correlations between changes in EEG parameters and changes in clinical assessments throughout treatment were modest and inconsistent, suggesting limited potential for these EEG parameters as predictive or treatment response biomarkers.
Schwartzmann et al. (2023) [210]	41	- A total of 41 adults with depression were recruited to undergo a 16-week course of cognitive behavioral therapy (CBT) - A total of 30 of these participants had resting-state electroencephalography (EEG) recordings at baseline and week 2 of the therapy - Successful clinical response to CBT was defined as a 50% or greater reduction in Montgomery–Åsberg Depression Rating Scale (MADRS) score from baseline to post-treatment completion - EEG relative power spectral measures were analyzed at baseline, week 2, and as early changes from baseline to week 2	- Lower baseline relative delta power predicted better response to cognitive behavioral therapy (CBT) in patients with depression. - Increases in relative delta power and decreases in relative alpha power from baseline to week 2 of CBT also predicted better response to the therapy. - The resting-state EEG measures show potential utility in predicting CBT outcomes and could inform clinical decision-making for treatment of depression.
Shereena et al. (2018) [211]	30	- Experimental longitudinal design with pre-post comparison - A total of 30 children with ADHD (6–12 years old) divided into the following groups: - Treatment group (n = 15): received 40 sessions of theta/beta neurofeedback training over 3.5–5 months, plus routine clinical management - Control group (n = 15): received only routine clinical management	- Neurofeedback training improved cognitive functions, behavior, and academic performance in children with ADHD compared to the control group. - The improvements seen in the neurofeedback training group were sustained at a 6-month follow-up assessment. - Neurofeedback training is an effective intervention for enhancing cognitive deficits and reducing ADHD symptoms and behavior problems in children with ADHD.
Sibalis et al. (2019) [212]	15	- Pre-post study design with EEG recording - Participants completed a single-point focus rest task and two active attention tasks - Measured theta power, beta power, and theta/beta ratio (TBR) from the EEG data - Compared a treatment group that received a mindfulness intervention to a waitlist control group	- The mindfulness treatment group showed significant improvements in attentional ability, as measured by a decreased theta/beta ratio (TBR), compared to the waitlist control group. - The study provides evidence that mindfulness treatment can enhance attentional control in youth with ADHD at the neural level, as measured by EEG. - The study offers methodological support for using active attention tasks, rather than just resting-state tasks, when examining the impact of mindfulness on attention in youth with ADHD.
Simpraga et al. (2018) [213]	28	- Four-way crossover design with 28 healthy subjects - Subjects received mecamylamine (nicotinic acetylcholine receptor antagonist), placebo, nicotine, or galantamine - Eyes-closed resting EEG recordings were performed during the treatments - Machine learning was used to develop an nAChR index consisting of 10 EEG biomarkers that could distinguish the effects of mecamylamine and placebo - The nAChR index was used to demonstrate the reversal of mecamylamine-induced neurophysiological effects by galantamine (16 mg) and nicotine (21 mg transdermal)	- The researchers developed a nicotinic acetylcholine receptor (nAChR) index based on 10 EEG biomarkers that can accurately distinguish the effects of the nAChR antagonist mecamylamine from placebo. - The nAChR index was able to show that the effects of mecamylamine (which induces cognitive dysfunction) could be reversed by administering the nAChR agonists galantamine and nicotine. The mecamylamine challenge model and the nAChR index could be valuable tools for evaluating the effects of drugs targeting the nicotinic cholinergic system, such as in the development of pro-cognitive compounds.
Spironelli & Angrilli (2017) [214]	32	- Between-subjects design with 16 participants in each of two groups: horizontal Bed Rest (hBR) and Sitting Control (SC) - Baseline EEG recording with both groups in a seated position (T0) - Experimental manipulation where the hBR group transitioned to a supine position while the SC group remained seated (T1) - Additional EEG recordings after 120 min in the assigned position (T2) and after participants returned to the seated position (T3)	- The supine body position led to a significant decrease in high-frequency EEG activity (high-beta and -gamma bands) compared to the seated position, and this effect lasted for the 2 h duration of the supine condition. - The supine position abolished the typical left-lateralized frontal activation in high-frequency EEG bands that were observed in the seated position. - The seated position was associated with greater activation in the left inferior frontal gyrus and left insula compared to the supine position.
Stolz et al. (2023) [215]	119	- Secondary data analysis of the publicly available TDBRAIN database - Logistic regression modeling to predict treatment response (defined as ≥50% improvement on the Beck’s Depression Inventory) in 119 MDD patients receiving repetitive transcranial magnetic stimulation (rTMS) - Examined age, baseline symptom severity, and EEG measures as predictors of rTMS treatment response	- Older age and more severe depression symptoms at baseline were associated with decreased odds of a positive response to rTMS treatment. - EEG measures showed some potential to improve prediction of treatment response, but the improvements were not statistically significant.
Strafella et al. (2023) [216]	114	- Randomized, triple-blind study design - Compared two schedules of intermittent theta burst stimulation (iTBS) treatment: separated (54 min interval) and contiguous (0 min interval) - A total of 30 sessions of iTBS treatment delivered - TMS-EEG measurements taken at baseline and post-treatment - Analysis focused on N45 and N100 components of TMS-evoked potentials using global mean field analysis	- The N100 amplitude, a TMS-EEG marker, decreased from baseline to post-treatment in both the separated and contiguous iTBS treatment groups. - Participants who responded to the iTBS treatment showed a decrease in N100 amplitude after treatment. - Responders to iTBS treatment had higher post-treatment N45 amplitude, another TMS-EEG marker, compared to non-responders. - Higher baseline N100 amplitude was associated with greater improvement in depression scores after iTBS treatment.
Styliadis et al. (2015) [217]	70	- A total of 70 right-handed individuals with mild cognitive impairment (MCI) were divided into five equally populated groups (14 participants per group) that underwent different training interventions (combined cognitive and physical training, cognitive training only, physical training only, active control, and passive control) - All training components were computerized, center-based, and under supervision - A 5 min resting-state EEG was recorded before and after the 8-week intervention - Cortical sources were modeled using exact low-resolution brain electromagnetic tomography (eLORETA) - Nonparametric statistical methods were used to compare the pre- and post-intervention EEG source activity within and between groups	- An 8-week combined physical and cognitive training program in MCI patients led to decreases in delta, theta, and beta brain rhythms in the precuneus/posterior cingulate cortex, which was associated with improvements in cognitive function. - The combined training was more effective than physical or cognitive training alone. - The physical training component played a key role in driving the neuroplastic changes observed.
Subramanian et al. (2022) [218]	25	The study uses a single-center, prospective, observational design called “Correlating ECT Response to EEG Markers (CET-REM)”. Participants with unipolar or bipolar depressive episodes will undergo an index course of ECT, with self-reported depressive scores assessed before each session. Overnight sleep EEG data will be collected using a wireless Dreem headband on post-ECT days, to measure sleep slow waves and sleep spindles. Optional high-density EEG data will also be recorded during the ECT-induced seizures, to quantify seizure markers like central-positive complexes (CPCs). Sleep EEG data will be analyzed using custom MATLAB scripts, and linear mixed-effects models will be used to examine changes in sleep markers over the course of ECT and their relationship with seizure markers	
Sun et al. (2015) [219]	14	- Within-subjects design with three EEG recording sessions per participant: DBS ON, DBS randomized ON/OFF, DBS OFF - Participants performed zero-back and three-back working memory tasks during each session - Final sample size of 14 participants with valid EEG data	- DBS stimulation suppressed frontal gamma oscillations, particularly during the more cognitively demanding three-back task. - Suppression of gamma oscillations during the three-back task was associated with a reduction in depressive symptoms. - DBS stimulation increased the coupling between theta and gamma oscillations during the three-back task, and this increase in coupling was also associated with a reduction in depressive symptoms.
Tacca et al. (2024) [220]	30	- A total of 30 participants were recruited through social media and met criteria of being 18+, experiencing depressive symptoms, and actively seeking counseling - Participants were randomly assigned to either a VR-EEG therapy group or a Zoom videoconferencing group - The VR-EEG group received counseling in a virtual natural forest environment with a therapist avatar, while the Zoom group received counseling via Zoom videoconferencing - Both groups received the same positive, solution-focused counseling protocol and completed pre- and post-test assessments	- The VR-EEG therapy system was rated as more restorative than the Zoom online counseling system. - The VR-EEG therapy and Zoom online counseling were equally effective in improving client mood and positivity. - The VR-EEG therapy and Zoom online counseling were equally effective in creating a positive therapeutic alliance.
Tanju (2016) [221]	67	- Case series study design with 67 intellectually disabled children aged 6–16 years (39 male, 28 female) - Participants received QEEG-guided neurofeedback treatment, with the goal of normalizing their brain activity - IQ was assessed before and after the neurofeedback treatment - The hypothesis was that normalizing brain activity would lead to improvements in intellectual functioning as measured by IQ scores	- Neurofeedback treatment resulted in statistically significant increases in Verbal IQ (>6 points), Performance IQ (>9 points), and Full Scale IQ (7 points) in patients with intellectual disability. - The study findings warrant further controlled studies using this neurofeedback methodology in patients with intellectual disability.
Teel et al. (2014) [222]	20	- A total of 13 control participants and 7 concussed participants - All participants underwent EEG baseline, ImPACT testing, and VR balance/spatial testing - Concussed participants were tested within 8 (5 ± 1) days after their injury - EEG measures of power and coherence were compared between groups, with the concussed group showing decreased power and altered coherence across the different testing modalities	- Concussed participants passed standard clinical concussion tests but showed abnormalities in their brain electrical activity (EEG) measures. - Concussed participants were able to compensate and achieve normal functioning by recruiting additional brain networks. - Clinicians should consider the electrophysiological deficits observed in concussed participants, even when they pass standard clinical tests, when making return-to-play decisions.
Trauberg et al. (2021) [223]	19	- A total of 19 patients underwent resting-state EEG (128 channels) before and after a 6-week training session - EEG data from frontal, central, and temporal regions were analyzed for alpha and theta/delta activity and how these related to a composite score of executive function over time - Data from all four centers of the larger multi-center study will be included in a source-based network analysis of the EEG	- The performance and improvement of executive functions in Parkinson’s disease patients with mild cognitive impairment correlated positively with high-frequency oscillations and negatively with low-frequency oscillations in the resting-state EEG. - Resting-state EEG can be used as a biomarker to track changes in neuropsychological performance, specifically executive function, over time in this patient population. - The authors plan to examine whether baseline resting-state EEG activity can predict the response to cognitive or movement training in Parkinson’s disease patients with mild cognitive impairment.
Trenado et al. (2023) [224]	19	- Participants: 19 Parkinson’s disease patients with mild cognitive impairment (PD-MCI), with 10 in a cognitive training (CT) group and 9 in a physical activity (PA) group - Data collection: resting-state EEG and neuropsychological assessments of executive function (EF) and attention, collected before and after the interventions - EEG analysis: focused on frontal cortical areas due to their relevance to cognitive function - Analyses: examined the joint effect of the CT and PA interventions on EF and attention, as well as the relationships between EEG power in the theta and alpha bands and these cognitive measures	- A significant joint effect of cognitive training (CT) and physical activity (PA) interventions on executive function in Parkinson’s disease patients with mild cognitive impairment. - A trend towards a joint effect of CT and PA on attention in PD-MCI patients. - Resting-state EEG measures of theta and alpha power in frontal areas can serve as a biomarker for the joint therapeutic effects of CT and PA interventions in PD-MCI patients.
Trivedi et al. (2016) [225]	300	- Randomized, placebo-controlled clinical trial of the antidepressant sertraline - Target sample of 300 participants with early-onset (≤30 years) recurrent major depressive disorder (MDD) - Initial 8-week trial of sertraline or placebo, with non-responders switched double-blind to either bupropion (for sertraline non-responders) or sertraline (for placebo non-responders) for an additional 8 weeks - Examination of clinical moderators (e.g., anxious depression, early trauma, gender) and biological moderators/mediators (e.g., brain imaging, EEG, cognitive tasks) at baseline and week 1	
Velikova et al. (2017) [226]	30	- A 12-week positive imagery training program for 30 healthy participants - Initial 2-day group training followed by individual home practice - Psychological and EEG evaluations at baseline and after the training - EEG analysis using LORETA software to assess changes in current source density and functional connectivity - Statistical analysis of psychological test scores using paired *t*-tests	- Positive imagery training led to improvements in depressive symptoms, life satisfaction, and self-efficacy in the participants. - The EEG analysis showed increased activity in brain regions involved in emotional regulation and imagery processing, as well as increased functional connectivity between these regions.
Vinne et al. (2021) [227]	195	- Open-label, prospective study design - Used pre-treatment EEG biomarkers (paroxysmal activity, alpha peak frequency, frontal alpha asymmetry) to guide clinicians in selecting between three antidepressant medications (escitalopram, sertraline, venlafaxine) - Compared this EEG-informed prescription to a treatment-as-usual (TAU) control group - Collected EEG data from 195 outpatients with major depressive disorder prior to 8 weeks of antidepressant treatment - Recruited patients to receive TAU first to establish a baseline, then recruited patients to receive the EEG-informed prescription	- The EEG-informed prescription approach was feasible, with 65% of clinicians following the recommendations compared to 60% in the treatment-as-usual group. - The EEG-informed prescription approach was confirmed to be feasible. - Clinicians and patients were satisfied with the EEG-informed prescription protocol.
Voetterl et al. (2021) [228]	39	- A total of 39 patients with major depressive disorder (MDD) were enrolled - Patients received a 5-week rTMS protocol: - Six daily sessions of accelerated low-frequency rTMS over the right dorsolateral prefrontal cortex (DLPFC) for the first 5 days - Followed by a tapering course of 25 once-daily rTMS sessions - Resting-state EEG and heart rate were measured at three time points: - Baseline - One week after the final accelerated session - Upon completion of the tapering course - The primary clinical outcome measure was the Beck Depression Inventory-II (BDI-II)	- High relative baseline theta power in prefrontal areas and high baseline heart rate were associated with poorer clinical outcomes to low-frequency rTMS treatment. - Heart rate decreased acutely after the first rTMS session, but this effect was not associated with treatment outcome.
Wang et al. (2023) [229]	82	- Participants: 82 patients with insomnia, with an average age of 49.38 ± 12.78 years (26 men, 56 women) - Intervention: biofeedback treatment, consisting of 5 min of EMG feedback and 30 min of EEG feedback per session, conducted every other day - Outcome measures: Pittsburgh Sleep Quality Index (PSQI), Beck Depression Inventory (BDI-II), and State–Trait Anxiety Inventory (STAI), measured before the first, fifth, and tenth sessions, and after the twentieth session - Study design: participants were divided into two groups—one that completed 10 biofeedback sessions and one that completed 20 sessions	- Biofeedback treatment significantly improved sleep quality, as measured by the Pittsburgh Sleep Quality Index (PSQI). - Biofeedback treatment significantly reduced symptoms of depression and anxiety, as measured by the Beck Depression Inventory (BDI-II) and the State–Trait Anxiety Inventory (STAI). - Biofeedback treatment was associated with decreased beta and theta power, increased alpha power, and decreased EMG activity in participants with insomnia.
Wang et al. (2019) [230]	87	- A total of 87 patients with comorbid major depressive disorder (MDD) and anxiety symptoms were allocated to one of three groups: ALAY (alpha asymmetry neurofeedback), Beta (high-beta down-training neurofeedback), or a control group. - The ALAY and Beta groups received 10 sessions of their respective neurofeedback interventions. - All participants completed the Beck Depression Inventory-II (BDI-II), Beck Anxiety Inventory (BAI), and 5 min of resting-state EEG recording at both pre-test and post-test.	- Both alpha asymmetry neurofeedback (ALAY) and high-beta down-training neurofeedback (Beta) were effective in reducing symptoms of depression and anxiety in patients with comorbid major depressive disorder (MDD) and anxiety symptoms. - The high-beta down-training neurofeedback (Beta) was more effective in decreasing high-beta power at the parietal cortex compared to the alpha asymmetry neurofeedback (ALAY) and the control group. - Both neurofeedback interventions were effective, but the high-beta down-training neurofeedback (Beta) was more effective in decreasing high-beta power in the parietal cortex.
Wei et al. (2024) [231]	60	- Collected resting-state EEG data from 70 PSD patients and 40 healthy controls (HC group) - Provided 6 weeks of acupuncture treatment to the PSD patients - Collected post-treatment EEG data from 60 PSD patients (MA group) - Divided the MA group into a remission prediction (RP) group and a non-remission prediction (NRP) group based on their response to acupuncture treatment - Developed a prediction model for acupuncture treatment efficacy using the baseline EEG microstate data	- The duration of microstate D and the occurrence and contribution of microstate C were reduced in PSD patients compared to healthy controls. - Acupuncture treatment partially normalized the abnormal EEG microstate patterns observed in PSD patients. - Baseline EEG microstates could predict the efficacy of acupuncture treatment for PSD patients with high accuracy (AUC = 0.964).
Woltering et al. (2015) [232]	39	- Participants were children with disruptive behavior problems referred for treatment, assessed using the CBCL - They received a 14-week combined cognitive behavioral therapy and parent management training program - Behavioral and EEG data were collected before, after, and 12 months after treatment - A Go/No-Go task was used to measure neural activity, specifically theta power, during inhibitory control - EEG was recorded using a 129-channel sensor net with a 250 Hz sampling rate	- Long-term improvers showed continuous reductions in fronto-midline theta power from baseline to follow-up compared to nonimprovers. - Reductions in theta power were found for both early and later processing phases for improvers, suggesting increased neural efficiency in attentional vigilance and inhibitory control. - The effects were stronger when participants were grouped based on improvements in internalizing symptoms rather than externalizing symptoms, suggesting theta power may be more sensitive to changes in anxiety.
Wu et al. (2020) [233]	309	- Used a machine learning algorithm tailored for resting-state EEG data - Applied the algorithm to a large, placebo-controlled antidepressant trial (n = 309) - Predicted symptom improvement in response to the antidepressant sertraline, compared to placebo - Validated the predictive model across multiple study sites and EEG equipment - Measured prefrontal neural responsivity using concurrent TMS and EEG	- The study developed a machine learning algorithm to analyze resting-state EEG data and predict symptom improvement specifically for the antidepressant sertraline, with this prediction being consistent across different study sites and EEG equipment. - The EEG signature predictive of sertraline response was also associated with prefrontal neural responsivity, as measured by concurrent transcranial magnetic stimulation and EEG.
Wu et al. (2024) [234]	48	- Participants: 48 patients with major depressive disorder (MDD) and anxiety symptoms - Study design: randomized controlled trial with a treatment group and a control group - Intervention: the treatment group received 10 sessions of standardized weighted low-resolution electromagnetic tomography Z-score neurofeedback (swLZNFB) twice weekly, while the control group received treatment as usual - Outcome measures: - Self-report questionnaires: Beck Depression Inventory-II (BDI-II) and Beck Anxiety Inventory (BAI) - Electroencephalography (EEG): number and percentage of EEG abnormalities, and current source density (CSD) in the prefrontal cortex, anterior cingulate cortex, posterior cingulate cortex, and amygdala - Comparison: outcomes were compared between the two groups at pre-test and post-test	- The swLZNFB group showed decreased depression and anxiety symptoms, as well as reduced EEG abnormalities, compared to the control group. - The swLZNFB group also showed decreased current source density in brain regions associated with depression and anxiety, indicating improvements in brain activity.
Yan et al. (2021) [235]	30	- A total of 30 drug-naïve MDD patients were enrolled and received antidepressant treatment - Sixty-four-channel EEG was recorded at baseline and 2 weeks of treatment, with eyes closed - EEG data were preprocessed to remove artifacts and extract microstate features - Microstate analysis was performed to identify four canonical microstate classes (A–D)	- The duration, occurrence, and proportion of microstate B decreased significantly after 2 weeks of antidepressant treatment. - The occurrence of microstate A increased after treatment, and this increase was negatively correlated with the reduction in anxiety symptoms. - Changes in EEG microstates, particularly microstate B and A, may be potential biomarkers for predicting early response to antidepressant treatment in patients with major depressive disorder.
Yang et al. (2023) [236]	309	(1) An automatic EEG preprocessing pipeline to extract standardized features (2) Using causal forests to estimate heterogeneous treatment effects (HTEs) (3) Employing an efficient policy learning algorithm to learn an optimal treatment assignment policy (4) Comparing the performance of the policy learning algorithm to other methods like Q-learning and outcome-weighted learning	- Non-invasive EEG measures of relative theta and alpha-band power can aid in detecting heterogeneous treatment effects and learning an optimal treatment assignment policy for depression. - The automatic EEG preprocessing and feature extraction procedure yields features with stronger signals compared to raw features. - Sertraline treatment demonstrates overall efficacy, with a significant average treatment effect of improving the response rate by 17.4% (95% CI: [2.6%, 32.2%]).
Yuan et al. (2024) [237]	86	- Clinical observational study design - Recruited 38 healthy controls and 48 MDD patients - Participants underwent EEG scans while viewing emotional facial expressions at weeks 0 and 1 - MDD patients received 4 weeks of antidepressant treatment and were categorized as responders or non-responders - Functional connectivity analysis using graph theoretical measures (node strength, global efficiency, cluster coefficient) - Multivariable linear regression to compare FC between MDD and healthy control groups, controlling for confounding variables	- Patients with major depressive disorder (MDD) showed significantly reduced functional connectivity in the brain during visual emotion processing compared to healthy controls. - MDD had a significant negative effect on functional connectivity in the brain. - Higher baseline functional connectivity in the delta-band frequency was associated with better treatment response in MDD patients.
Zajecka et al. (2024) [238]	66	- Open-label study design - A total of 25 adult MDD patients with cognitive dysfunction received 8 weeks of vortioxetine treatment - ERP data collected during cognitive tasks at pre-treatment, 2 weeks, and 8 weeks - Compared ERP characteristics of MDD group to 41 healthy controls at baseline and endpoint	- Compared to healthy controls, MDD patients exhibited increased latencies of P200 and P3b ERP components at baseline, which normalized after 8 weeks of vortioxetine treatment. - Changes in P200 and P300 ERP measures were correlated with improvements in both clinical symptoms and cognitive functioning in MDD patients, indicating a pro-cognitive effect of vortioxetine independent of its antidepressant effects.
Zandvakili et al. (2020) [239]	47	- Eight-channel resting-state EEG data collected on participants before (n = 47) and after (n = 43) a randomized controlled trial of iTBS for PTSD - iTBS delivered to the right dorsolateral prefrontal cortex for 10 sessions at 80% of motor threshold and 1800 pulses - Cross-validated support vector machine (SVM) used to analyze EEG data and detect changes in functional connectivity after active iTBS treatment	- The study used a cross-validated support vector machine (SVM) to track changes in EEG functional connectivity after intermittent theta burst stimulation (iTBS) treatment for post-traumatic stress disorder (PTSD). - The SVM classifier was able to successfully separate patients who received active iTBS treatment from those who received sham treatment, with statistically significant findings in the delta band (1–4 Hz). - The Delta coherence changes observed represented an increase in functional connectivity between midline central/occipital regions and a decrease between frontal and central regions.
Zhang et al. (2016) [240]	21	The study used a combination of thermal pain stimulation, 64-channel EEG recording, and data preprocessing techniques including ICA to investigate the neural correlates of sustained thermal pain in healthy participants.	- Tonic thermal pain stimulation led to a global decrease in lower-frequency brain rhythms, especially in the alpha band. - The degree of alpha power reduction was linearly correlated with the subjective pain ratings reported by the participants. - Granger causality analysis showed changes in connectivity between pain-related brain regions during high-intensity pain stimulation compared to innocuous warm stimulation.
Zhang et al. (2021) [241]	9	- Nine patients with primary central sleep apnea syndrome (CSAS) were enrolled in the study - Raw sleep EEG data were analyzed using the following: - Fractal dimension (FD) and zero-crossing rate of detrended FD - Conventional EEG spectral analysis in delta, theta, alpha, and beta bands using fast Fourier transform - The study compared FD values between NREM and REM sleep in patients with CSAS before and after CPAP treatment	- CPAP treatment decreased fractal dimension (FD) in NREM sleep but increased FD in REM sleep in patients with primary central sleep apnea syndrome (CSAS). - CPAP treatment increased alpha power and decreased the delta/alpha ratio during REM sleep in patients with primary CSAS.
Zhang et al. (2018) [242]	26	- Independent component analysis (ICA) and graph theory analysis to examine functional brain networks based on power spectral density (PSD) of resting-state EEG data - Nonparametric permutation tests to compare network metrics between MDD and healthy control groups - Pearson correlation analysis to assess the relationship between network metrics and clinical symptoms of depression	- Compared to healthy controls, individuals with major depressive disorder (MDD) showed significant randomization of their functional brain networks, with greater global efficiency but lower local efficiency. - The randomized brain networks in MDD patients were more resilient to both random and targeted attacks, which could be a protective mechanism. - The MDD brain networks had a lower rich-club coefficient, indicating sparser connections between high-degree “rich-club” hub nodes.
Zhang et al. (2023) [243]	170	- Study 1: - Randomized, sham-controlled design - A total of 50 insomnia disorder (ID) patients - A total of 20 sessions of 1 Hz rTMS over the left dorsolateral prefrontal cortex - Measured EEG, polysomnography, and clinical assessments before and after rTMS - Study 2: - A total of 120 ID patients received active rTMS treatment - Patients were divided into optimal and suboptimal response groups based on Pittsburgh Sleep Quality Index reduction rate - Baseline EEG coherence was used to develop predictive models for rTMS treatment effects	- Decreased EEG coherence in theta and alpha bands were observed after rTMS treatment, and changes in theta-band (F7-O1) coherence were correlated with changes in sleep efficiency. - Baseline EEG coherence in theta, alpha, and beta bands showed the potential to predict the treatment effects of rTMS for insomnia disorder. - rTMS improved the sleep quality of insomnia disorder patients by modulating their abnormal EEG coherence.
Zuchowicz et al. (2019) [244]	18	- The study was conducted at the Grenoble University Hospital with approval from the local ethics committee and informed consent from all participants. - The study included 10 patients with bipolar disorder (BP) and 8 patients with major depressive disorder (MDD), with demographic information provided. - Repetitive transcranial magnetic stimulation (rTMS) was applied to the left dorsolateral prefrontal cortex (DLPFC) at 10 Hz for 2000 pulses per session. - EEG data were recorded before and after the 1st, 10th, and 20th rTMS sessions using a 64-channel system at a 2500 Hz sampling rate. - The Phase-Locking Value (PLV) was used as a measure of functional connectivity between EEG signals to assess the impact of rTMS on brain activity.	- The PLV, a measure of phase synchronization between EEG signals, increased over the course of rTMS treatment in both MDD and BP patients, and this increase was associated with response to treatment. - The study found increased connectivity between left frontal and right parieto-occipital regions after rTMS, which may indicate a neural marker of treatment response. - The PLV indices were greater in the gamma band after rTMS for both responder groups compared to non-responders, and greater in the delta band for responders compared to non-responders before and after stimulation.

**Table 3:** EEG biomarkers across disorders (transdiagnostic).

Biomarker	Consistently Associated Disorders	Insights
P300	Schizophrenia, depression, ADHD, dementia, anxiety	Reduced amplitude and delayed latency are widespread; reflects attention and working memory deficits.
MMN	Schizophrenia, dementia, autism	Strong marker for early sensory processing; especially robust in schizophrenia.
ERN	Anxiety, OCD, depression	Increased amplitude in anxiety; relates to error monitoring and cognitive control.
Alpha Asymmetry	Depression, anxiety	Especially frontal alpha asymmetry in depression; linked to affective processing and mood regulation.
Gamma Power	Schizophrenia, autism, depression	Reflects cognitive binding and integration; often reduced in high-order cognitive tasks.
Connectivity Abnormalities	All conditions (esp. schizophrenia, autism, depression)	Reflects impaired network organization and synchronization; useful for dimensional diagnostics.

**Table 4:** Disorder-specific EEG biomarkers.

Disorder	Key EEG Biomarkers
Schizophrenia	P300 ↓, MMN ↓, Gamma ↓, Connectivity ↓, ERN ↑
Depression	P300 ↓ (moderate), Alpha Asymmetry ↑ (left frontal), Reward Positivity ↓
ADHD	Theta/Beta Ratio ↑, P300 ↓, CNV ↓
Anxiety Disorders	ERN ↑, Beta/Gamma ↑, Threat-Related Early ERP ↑
Autism Spectrum	Gamma ↓, Early ERP Changes, Over-/Under-Connectivity
Dementia	P300 ↓, MMN ↓, Increased Delta/Theta, Decreased Alpha/Beta

**Table 5:** Key insights of EEG-based cognitive measures in neuropsychiatric disorders.

Key Finding	Clinical Relevance	Indicative Supporting Studies
Executive function shows the strongest correlation with functional outcomes across disorders	Clinical Assessment	223, 187, 204, 168, 215, 241
EEG biomarkers show significant potential for predicting symptom progression and treatment response	Predictive Medicine	143, 184, 227, 154, 203, 167, 229
Parkinson’s disease has the most robust evidence linking EEG cognitive measures to functional capacity	Disorder Specificity	223, 187, 204, 168, 214, 242
Both ERPs (especially P300) and frequency measures (alpha/theta) provide valuable clinical information	Methodological Approach	135, 149, 168, 163, 182, 201
Longitudinal studies demonstrate that certain EEG markers can predict functional decline before clinical manifestation	Early Detection	153, 200, 237, 164, 208, 243
The relationship between EEG measures and functional outcomes is moderated by cognitive reserve	Individual Differences	149, 192, 232, 160, 204, 240
EEG measures show promise for personalizing cognitive interventions to optimize functional improvement	Treatment Customization	146, 191, 232, 162, 207, 241

**Table 6:** Summary of methodological reporting quality across 132 included studies.

Methodological Element	Percentage of Studies Reporting	Key Examples
Standardized EEG protocols (e.g., 10–20 system)	18.2%	[118,173,202]
Reliability/reproducibility as primary focus	0.1%	[124,198]
Clinical vs. control population comparison	75.0% clinical, 14.4% healthy controls only, 10.6% mixed	[142,157,183]
Preprocessing pipeline details	46.2%	[119,137,164,215]
Reference schemes	38.6%	[122,156,195,228]
Robust reliability metrics (ICC, test–retest)	11.0%	[143,165,206,233]
Statistical power/sample size calculations	7.6%	[134,158,197]
Independent sample validation	13.3%	[139,161,190]
Replication attempts of previous findings	5.3%	[146,175,199,227]
Detailed artifact rejection procedures	41.7%	[138,152,189,241]
Funding sources and conflicts disclosure	67.4%	[132,166,203,238]
Medication status of participants	52.3%	[131,145,167,228]

**Table 7:** Differential potential of EEG-based cognitive biomarkers across mental health applications and implementation contexts.

Domain	Key Findings	Implications
Application Potential	Early detection strongest for schizophrenia (85%) and dementia (80%) Risk stratification most promising for schizophrenia (88%) Population screening highest for ADHD (78%) and autism (72%) Treatment monitoring valuable for depression (82%) and anxiety (78%)	Different conditions benefit from distinct biomarker applications; implementation should be condition-specific rather than universal
Implementation Factors	Evidence strength most robust for schizophrenia (82%) and dementia (80%) Technical scalability highest for ADHD (85%) and autism (75%) Cost-effectiveness most favorable for ADHD (80%) Public health impact greatest for dementia (85%) and depression (82%)	Strategic implementation should prioritize applications with strongest supporting factors and clear cost–benefit advantages
Healthcare Integration	Conditions with prodromal states show strongest early detection potential Developmental conditions (ADHD, autism) demonstrate highest technical scalability High-prevalence conditions show strongest potential for treatment monitoring Implementation readiness highest for conditions common in primary care	Initial implementation efforts should target high-feasibility applications while developing infrastructure for broader deployment

## Abbreviations

The following abbreviations are used in this manuscript:

Neuroimaging Techniques	
MEG	Magnetoencephalography
MRI	Magnetic Resonance Imaging
PET	Positron Emission Tomography
fMRI	Functional Magnetic Resonance Imaging
sMRI	Structural Magnetic Resonance Imaging
LORETA	Low-Resolution Electromagnetic Tomography
tDCS	Transcranial Direct Current Stimulation
rTMS	Repetitive Transcranial Magnetic Stimulation
TMS	Transcranial Magnetic Stimulation
qEEG	Quantitative Electroencephalography
MMN	Mismatch Negativity
Neuropsychiatric Disorders	
ADHD	Attention-Deficit/Hyperactivity Disorder
ASD	Autism Spectrum Disorder
BPD	Borderline Personality Disorder
OCD	Obsessive–Compulsive Disorder
MDD	Major Depressive Disorder
SCD	Social Communication Disorder
UWS	Unresponsive Wakefulness Syndrome
MCS	Minimally Conscious State
AD	Alzheimer’s Disease
Cognitive Biomarkers	
ERP	Event-Related Potential
EEG	Electroencephalography
APF	Alpha Peak Frequency
TBR	Theta/Beta Ratio
PANSS	Positive and Negative Syndrome Scale
MADRS	Montgomery–Åsberg Depression Rating Scale
Public Health and Research	
PRISMA	Preferred Reporting Items for Systematic Reviews and Meta-Analyses
OSF	Open Science Framework
ESEMeD	European Study of the Epidemiology of Mental Disorders
WHO	World Health Organization
SCAN	Schedules for Clinical Assessment in Neuropsychiatry
CIDI	Composite International Diagnostic Interview
PwDS	Persons with Down Syndrome
